# Structure of a human replisome shows the organisation and interactions of a DNA replication machine

**DOI:** 10.15252/embj.2021108819

**Published:** 2021-10-25

**Authors:** Morgan L Jones, Yasemin Baris, Martin R G Taylor, Joseph T P Yeeles

**Affiliations:** ^1^ MRC Laboratory of Molecular Biology Cambridge UK

**Keywords:** CMG helicase, cryo‐EM, DNA replication, fork protection complex, replisome, DNA Replication, Recombination & Repair, Structural Biology

## Abstract

The human replisome is an elaborate arrangement of molecular machines responsible for accurate chromosome replication. At its heart is the CDC45‐MCM‐GINS (CMG) helicase, which, in addition to unwinding the parental DNA duplex, arranges many proteins including the leading‐strand polymerase Pol ε, together with TIMELESS‐TIPIN, CLASPIN and AND‐1 that have key and varied roles in maintaining smooth replisome progression. How these proteins are coordinated in the human replisome is poorly understood. We have determined a 3.2 Å cryo‐EM structure of a human replisome comprising CMG, Pol ε, TIMELESS‐TIPIN, CLASPIN and AND‐1 bound to replication fork DNA. The structure permits a detailed understanding of how AND‐1, TIMELESS‐TIPIN and Pol ε engage CMG, reveals how CLASPIN binds to multiple replisome components and identifies the position of the Pol ε catalytic domain. Furthermore, the intricate network of contacts contributed by MCM subunits and TIMELESS‐TIPIN with replication fork DNA suggests a mechanism for strand separation.

## Graphical abstract

Cryo‐EM structures of reconstituted human core replisomes show how TIMELESS‐TIPIN, AND‐1, Pol ε and CLASPIN engage the CMG helicase, and suggest a mechanism for DNA strand separation.

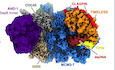

## Introduction

Molecular machines participate in all aspects of cellular function including protein synthesis, gene transcription and chromosome replication. The latter is accomplished by the coordinated activities of multiple diverse proteins functioning together as the replisome. Understanding how these activities synergise requires detailed structural knowledge of replisomes engaged with DNA. Recent advances in budding yeast replisome reconstitution and electron cryomicroscopy (cryo‐EM) have yielded unprecedented new insights into replisome structure and mechanism (Yeeles *et al*, 2015, 2017; Georgescu *et al*, 2017; Douglas *et al*, 2018; Eickhoff *et al*, 2019; Baretić *et al*, 2020; Rzechorzek *et al*, 2020). By contrast, the current picture of the human replisome is more limited and only high‐resolution structures of individual components have so far been determined. Moreover, due to the additional complexity of human cells, the human replisome contains additional factors that do not have orthologs in yeast. These include proteins involved in replisome disassembly, replisome stability and coupling DNA replication to repair, some of which are essential and/or mutated in genetic conditions (Reynolds *et al*, 2017; Bellelli & Boulton, 2021; Wu *et al*, 2021). Direct structural investigation of human replisomes is therefore critical to uncover mechanisms that underpin accurate and efficient chromosome replication in human cells.

The molecular machine that unwinds template DNA during replication, and around which the replisome is built, is the CDC45‐MCM‐GINS (CMG) helicase. CMG is a hexameric ring of related MCM2‐7 subunits stabilised by CDC45 and the tetrameric GINS complex (Costa *et al*, 2011; Yuan *et al*, 2016). Each MCM subunit has distinct N‐ and C‐terminal domains that form two tiers referred to as the N‐ and C‐tier. The C‐tier harbours the AAA+ ATPase domains that power DNA unwinding, while the N‐tier contains helical, oligonucleotide/oligosaccharide‐binding (OB) and zinc finger (ZnF) domains. CMG translocates in a N‐tier first orientation (Georgescu *et al*, 2017; Douglas *et al*, 2018), with the leading‐strand template pulled 3′–5′ through a central pore in the MCM ring. Cryo‐EM structures of drosophila CMG unwinding a model replication fork revealed several translocation states suggesting a non‐symmetric rotary mechanism for ssDNA translocation (Eickhoff *et al*, 2019). This model is supported by a 3.3 Å structure of human CMG (hsCMG) bound to single‐stranded DNA and ATP‐γ‐S (Rzechorzek *et al*, 2020).

As the leading‐strand template is pulled through the MCM central pore, the lagging‐strand template is excluded (Fu *et al*, 2011). All structural data indicate the point of template unwinding sits within a secondary N‐tier ring formed by the ZnF domains (Georgescu *et al*, 2017; Goswami *et al*, 2018; Eickhoff *et al*, 2019; Baretić *et al*, 2020; Yuan *et al*, 2020a). A recent structure of *S. cerevisiae* CMG (scCMG) bound to fork DNA visualised a network of interactions between MCM subunits and DNA that appear to block the lagging strand from entering the central pore and divert it towards a putative exit channel between the MCM3 and MCM5 ZnFs (Yuan *et al*, 2020a). We observed a similar configuration in a structure of scCMG bound to fork DNA, the fork protection complex (FPC) and Ctf4 (Baretić *et al*, 2020). Additionally, the N‐terminal hairpin (NTH) of MCM7 was positioned against the final base pair of duplex DNA, suggesting it might function as a strand separation pin.

Three replicative DNA polymerases function within the replisome. Pol α initiates synthesis and the high‐fidelity Pol δ and Pol ε perform the bulk of lagging‐ and leading‐strand replication, respectively (Nick McElhinny *et al*, 2008; Guilliam & Yeeles, 2020). Evidence from *S. cerevisiae* suggests all three polymerases are retained in the replisome for prolonged periods (Kapadia *et al*, 2020; Lewis *et al*, 2020). Although it is currently unclear how Pol α and Pol δ are localised to replication forks, Pol ε forms a stable complex with scCMG (Sengupta *et al*, 2013; Langston *et al*, 2014; Sun *et al*, 2015; Goswami *et al*, 2018). Here, the Pol ε subunits Pol2 and Dpb2 (human POLE1 and POLE2, respectively) contact Mcm2, 3, 5 and GINS (Goswami *et al*, 2018). Notably, the Pol ε catalytic domain, which is part of the Pol2 subunit, has not been visualised in the budding yeast replisome owing to its flexible tethering (Zhou *et al*, 2017; Goswami *et al*, 2018). Recently, a cryo‐EM structure of isolated budding yeast Pol ε showed Pol2 in a rigid linear conformation mediated by the Dpb3 and Dpb4 subunits (human POLE4 and POLE3, respectively) (Yuan *et al*, 2020b). It is currently unknown whether human Pol ε can adopt this configuration and whether it represents an active form of the polymerase in the replisome.

The FPC is composed of TIMELESS‐TIPIN and CLASPIN (*S. cerevisiae* Tof1‐Csm3 and Mrc1). It is essential for rapid and efficient replisome progression (Szyjka *et al*, 2005; Tourriere *et al*, 2005; Petermann *et al*, 2008; Somyajit *et al*, 2017; Yeeles *et al*, 2017) and coupling replication to other processes including sister‐chromatid cohesion (Chan *et al*, 2003; Leman *et al*, 2010; Cortone *et al*, 2018) and checkpoint activation (Kumagai & Dunphy, 2000). AND‐1 (*S. cerevisiae* Ctf4) is a trimeric scaffold protein that binds directly to CMG and functions as a hub to recruit additional proteins to the replication fork (Simon *et al*, 2014; Samora *et al*, 2016; Villa *et al*, 2016). We recently determined a high‐resolution cryo‐EM structure of the FPC and Ctf4 bound to scCMG and model replication fork DNA (Baretić *et al*, 2020). Ctf4 contacts scCMG predominantly via the β‐propeller of its SepB domain at the interface between GINS and CDC45 (Yuan *et al*, 2019; Baretić *et al*, 2020). A 6.7 Å cryo‐EM structure of hsCMG bound to AND‐1 showed this configuration is conserved but had insufficient resolution to reveal details of the interactions (Rzechorzek *et al*, 2020). The Tof1‐Csm3 heterodimer has an α‐solenoid structure and is positioned at the front of the replisome in advance of Mcm2, 6, 4 and 7 (Baretić *et al*, 2020). This positioning enables Tof1‐Csm3 to grip dsDNA before strand separation to ensure the replisome responds appropriately to protein barriers (Baretić *et al*, 2020). Tof1‐Csm3:MCM binding is mediated by two large loops inserted between helical repeats in the Tof1 α‐solenoid termed the Ω‐loop and MCM‐plugin (Baretić *et al*, 2020). These loops were not present in the crystal structure of the N‐terminal half of human TIMELESS (Holzer *et al*, 2017), and there are currently no structures of TIMELESS‐TIPIN in isolation or bound to hsCMG. While Mrc1 was present in our yeast replisome preparations, we did not recover density that could be unambiguously assigned to the protein. Cross‐linking mass spectrometry (XL‐MS) showed Mrc1 was positioned across one side of the replisome extending from Tof1, across Mcm2 and 6, towards Cdc45 (Baretić *et al*, 2020). It is yet to be determined if CLASPIN is similarly positioned in the human replisome.

Although much of the eukaryotic replisome is highly conserved, including Pol ε, TIMELESS‐TIPIN, CLASPIN and AND‐1, important structural similarities and differences between yeast and human replisomes will not be known until a high‐resolution human replisome structure is determined. Nor will there be an experimental system to directly investigate the structure of replisomes containing factors that are critical for human DNA replication but absent from yeast. To address these matters, and to establish how hsCMG coordinates replication fork DNA, Pol ε, the FPC and AND‐1, we have determined the cryo‐EM structure of the core human replisome at an overall resolution of 3.2 Å.

## Results

### Assembly of the core human replisome for cryo‐EM

**Figure 1:**
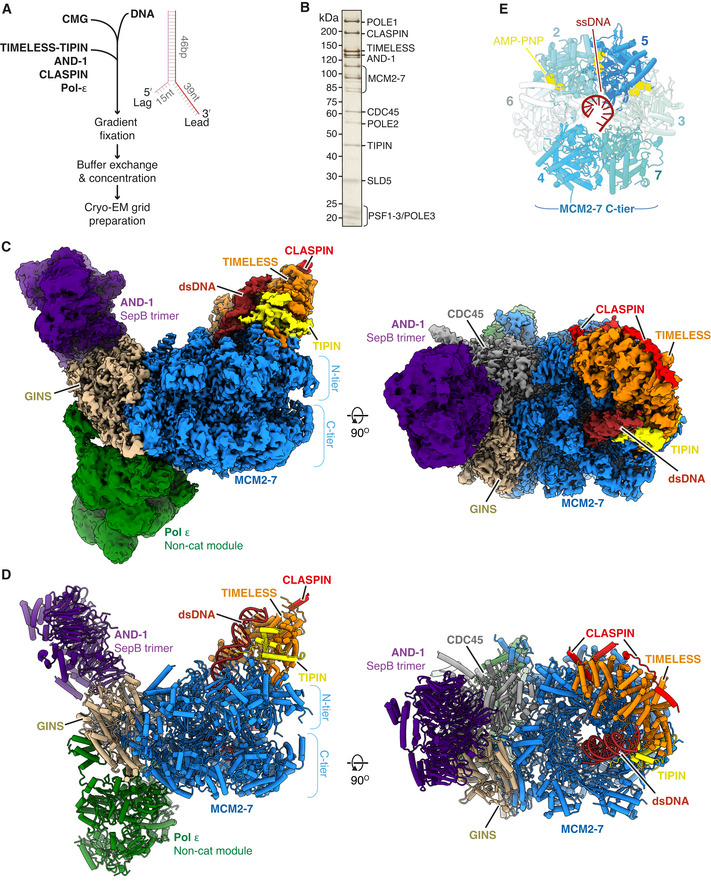
Cryo‐EM structure of the core human replisome ASchematic illustrating the in vitro reconstitution method of sample preparation for cryo‐EM experiments.BSilver‐stained SDS–PAGE of a peak fraction from a native glycerol gradient (Fraction 13, Appendix Fig S1C).C, DMultiple views of the Cryo‐EM density map (C) and the corresponding atomic model displayed as stubs and cylinders (D) for the core human replisome. The cryo‐EM density displayed in (C) is a composite map generated by combining the highest resolution regions of discrete refinements using Phenix Combine‐focussed‐maps.EEnd‐on view of the MCM2‐7 C‐tier illustrating which subunits engage ssDNA, and AMP‐PNP occupancy.

To prepare human replisomes for cryo‐EM, we utilised the method we developed for the budding yeast replisome (Baretić *et al*, 2020). Here, CMG is bound in the presence of the non‐hydrolysable ATP analogue AMP‐PNP to a replication fork consisting of a 46 bp duplex with non‐complementary 39 nt leading and 15 nt lagging single‐stranded DNA arms (Fig 1A). Additional replisome proteins are added and complexes are isolated through glycerol gradients. Prior to complex assembly, we confirmed that our preparations of hsCMG and Pol ε were proficient for DNA helicase and polymerase activity, respectively (Appendix Fig S1A and B). Considering the behaviour of their budding yeast counterparts (Baretić *et al*, 2020), we reasoned that TIMELESS‐TIPIN, CLASPIN, AND‐1 and Pol ε might stably associate with hsCMG. Indeed, Fig 1B and Appendix Fig S1C show that all four proteins co‐migrated with hsCMG in a native glycerol gradient. For cryo‐EM sample preparation, glycerol gradients were performed in the presence of glutaraldehyde and BS^3^ because mild cross‐linking improved data quality for budding yeast replisome reconstructions (Baretić *et al*, 2020) (Appendix Fig S1D). Peak gradient fractions were pooled, concentrated, buffer exchanged and applied to EM grids, and data were collected on a Titan Krios equipped with either a K2 Summit or Falcon 3 detector (Appendix Fig S1E and Table 1).

**Table 1:** Cryo‐EM data collection.

	Replisome (+) CLASPIN			
	Data collections	Data collection	Merged collections	Replisome (−) CLASPIN
	#1 and #2	#3	#1‐3	Data collection #1
Data collection and processing				
Grids	Cu R2/2 400 mesh (Quantifoil)	Cu R2/2 400 mesh (Quantifoil)		Cu R2/2 400 mesh (Quantifoil)
Surface	Continuous carbon	Continuous carbon		Continuous carbon
Freezing method	Manual plunger	Manual plunger		Manual plunger
Microscope	Titan Krios (Thermo)	Titan Krios (Thermo)		Titan Krios (Thermo)
Detector	K2 Summit (Gatan)	Falcon III (Thermo)		K2 Summit (Gatan)
GIF slit width (keV)	20	N/A		20
Number of micrographs	4,923	2,400		2,998
Voltage (kV)	300	300		300
Electron exposure (e^−^/Å^2^)	39.8	37.5		39.2
Defocus range (μm)	(−1.5) to (−3.5)	(−1.5) to (−3.5)		(−1.5) to (−3.5)
Pixel size (Å)	1.145	1.07		1.1
Symmetry	C1	C1		C1
Initial particle images (no.)	324,532	165,578		482,101
Final particle images (no.)	72,442	37,824	110,266	107,833
Map resolution (Å)	3.6	3.7	3.2	3.4
FSC threshold	0.143	0.143	0.143	0.143
Map resolution range (Å)	3.3–10	3.4–10	2.8–8	3–8

### Overall structure of the core human replisome

After data processing (Appendix Figs S1 and S2), we obtained a three‐dimensional (3D) reconstruction of the core human replisome at an overall map resolution of 3.2 Å, with good density for hsCMG, TIMELESS‐TIPIN, AND‐1 and Pol ε (Fig 1C). This, together with multi‐body refinement (Nakane *et al*, 2018) (see Materials and Methods and Appendix Figs S1 and S2 for details), enabled modelling of hsCMG, the majority of TIMELESS‐TIPIN, the trimeric SepB domains of AND‐1 and approximately half of Pol ε comprising the POLE2 subunit and the C‐terminal non‐catalytic domain of the POLE1 catalytic subunit (POLE1^nonCat^), collectively termed the Pol ε non‐cat module (Fig 1D and Table 2). We also observed five regions of well‐resolved but disconnected density that were initially challenging to identify. However, subsequent analysis—that we describe in detail in a later section of the manuscript—demonstrated that four of these regions were dependent on CLASPIN, which enabled us to build an atomic model encompassing three regions of the CLASPIN N‐terminus.

**Table 2:** Model refinement and validation statistics.

	Core replisome
Refinement	
Model resolution (Å) (FSC 0.5)	3.2
Map‐sharpening B‐factor (Å^2^)	−40
Model composition	
Non‐hydrogen atoms	67,592
Protein residues	8,367
Ligands	3 AMP‐PNP, 3 Mg^2+^, 5 Zn^2+^, SO4^2‐^
RMS deviations	
Bond lengths (Å)	0.006
Bond angles (°)	0.922
Validation	
MolProbity score	0.88
Clashscore	0.34
Poor rotamers (%)	0.77
Ramachandran plot	
Favoured (%)	96.23
Allowed (%)	3.74
Outliers (%)	0.02

**Figure EV1:**
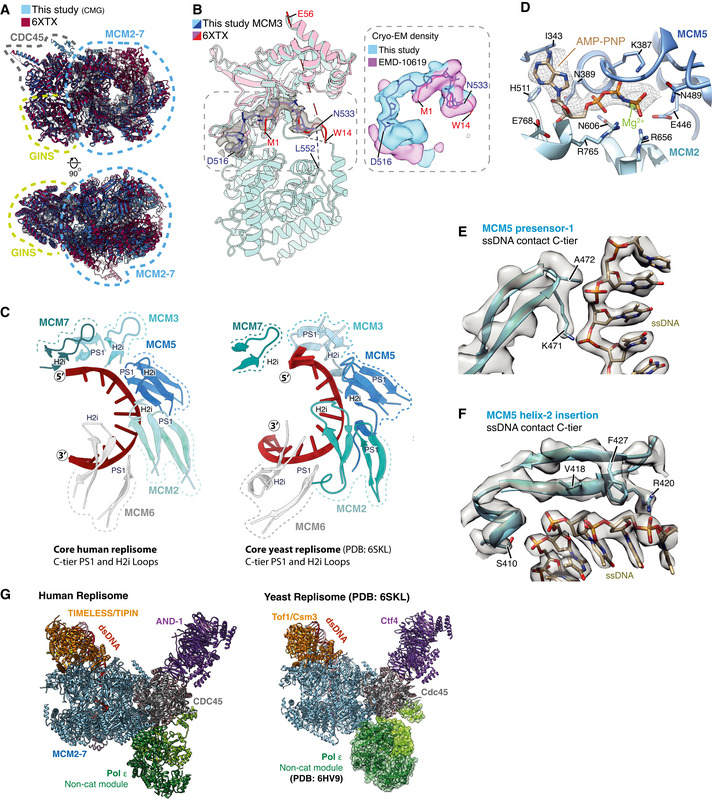
(Related to Fig 1). MCM2‐7 engagement with ssDNA in the C‐tier AComparison of the MCM N‐tier, GINS and CDC45 between the core replisome structure from this study (blue) (PDB: 7PFO) and the hsCMG:ssDNA structure (red) (PDB: 6XTX) (Rzechorzek *et al*, 2020).B(Left) Comparison of the MCM3 model from this study (blue) with the N‐tier lobe of MCM3 from the hsCMG:ssDNA structure (pink). Cryo‐EM density for residues D516‐N533 from this study is shown as a transparent grey surface. This region was assigned to the N‐terminal extension of a longer MCM3 isoform in the hsCMG:ssDNA structure (Rzechorzek *et al*, 2020). (Right) Overlay of cryo‐EM density from this study (blue) and the previous hsCMG:ssDNA map (pink). Despite the isoform used in this study not containing this N‐terminal extension, almost identical density is observed, which in our map shows clear connectivity with the MCM3 C‐terminal domain. We therefore attribute this density to MCM3 residues 524–533.CComparison of ssDNA engagement by the PS1 and H2i loops between the core human (left) and *S. cerevisiae* replisomes (right) (PDB: 6SKL) (Baretić *et al*, 2020).DDetailed view of the MCM2:5 ATPase site. Cryo‐EM density for AMP‐PNP is shown as mesh.E, FModels illustrating MCM5 engagement with ssDNA in the C‐tier. Cryo‐EM density represented as transparent grey surface with selected side chains contacting ssDNA displayed. (E) Representative density for the PS1 loop (MCM5) interacting with the phosphodiester ssDNA backbone. (F) Representative density for the H2i loop (MCM5) interacting with ssDNA in the C‐tier.GComparison of the core human (left) and *S. cerevisiae* (right) (PDB: 6SKL) replisomes. Models are coloured as in Fig 1C. For the *S. cerevisiae* replisome, the Pol ε non‐cat module has been positioned based on the structure of scCMG:Pol ε (PDB: 6HV9) (Goswami *et al*, 2018).

The conformations of the MCM N‐tier, CDC45 and GINS are very similar to those observed for isolated hsCMG (Rzechorzek *et al*, 2020) (Fig EV1A). The prior hsCMG preparation used an MCM3 isoform containing 45 additional N‐terminal amino acids (a.a.) and assigned a region of density between the MCM3 N‐tier and PSF3 (GINS subunit) to the first 14 a.a. of this extension (Rzechorzek *et al*, 2020). However, despite our MCM3 construct lacking these amino acids, we observed almost identical density in this region that we attribute to MCM3 residues 524‐533 (Fig EV1B). In contrast to the hsCMG:ssDNA:ATP‐γ‐S structure, where ssDNA is held in the C‐tier pore by MCM6, 4, 7 and 3 (Rzechorzek *et al*, 2020), we observe 11 nt of ssDNA bound on the opposite side of the pore engaging MCM3, 5, 2 and 6 (Figs 1E and EV1C). Accordingly, clear density for AMP:PNP is visible at the MCM3:5, 5:2 and 2:6 interfaces (Figs 1E and EV1D). This configuration is very similar to one of the three conformations (conformation 1) we observed for scCMG bound to fork DNA, the FPC and Ctf4 in the presence of AMP‐PNP (Baretić *et al*, 2020), and the manner in which ssDNA is coordinated by the presensor 1 (PS1) hairpins and helix 2 (H2)/helix 2 insertion (H2I) loops that protrude into the MCM central pore, is almost identical (Fig EV1C, E and F). Importantly, the conformation we observe here, and the conformation observed for the hsCMG:ssDNA:ATP‐γ‐S structure (Rzechorzek *et al*, 2020), are both similar to conformational states observed for drosophila CMG in the presence of ATP (Eickhoff *et al*, 2019), indicating that they represent distinct translocation states of CMG.

The overall architecture of the core human replisome is remarkably similar to *S. cerevisiae* (Fig EV1G). The Pol ε non‐cat module is positioned to the rear of the replisome on the C‐tier side of hsCMG beneath CDC45 and GINS, whereas the disc‐like AND‐1 trimer is bound side‐on to CDC45 and GINS on the N‐tier side of the complex (Fig 1C and D). At the front of the replisome, the parental DNA duplex extends approximately 2 turns from the central pore and is tilted towards the TIMELESS‐TIPIN heterodimer that sits on the leading edge of hsCMG. Like Mrc1 in the yeast replisome (Baretić *et al*, 2020), CLASPIN extends across one side of the human replisome where it directly contacts TIMELESS and MCM subunits (Fig 1C and D).

### AND‐1 docking in the human replisome

**Figure 2:**
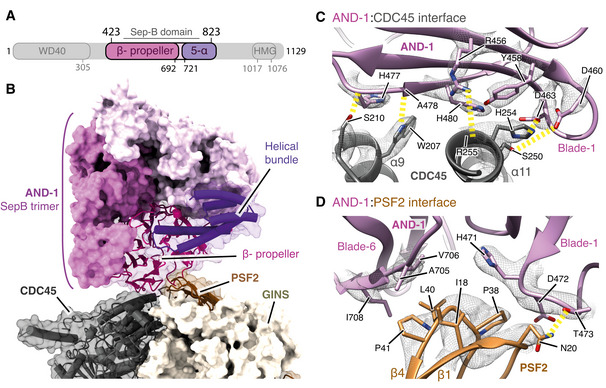
Structure of AND‐1 in the replisome Schematic for the domain architecture of AND‐1. Regions visualised in this structure are coloured, with domain boundaries demarcated by primary sequence numbering.Model of the AND‐1 SepB domain trimer bound to CMG rendered as a surface. The AND‐1 monomer mediating the interaction with PSF2 and CDC45 is displayed using both transparent surface rendering and backbone cartoon rendering.Detailed view of the AND‐1:CDC45 interface. Side chains of residues key to this interaction are displayed and annotated. The cryo‐EM density for these selected residues is displayed as a transparent mesh with yellow dashed lines indicating hydrogen‐bond formation.Detailed view of the interface between AND‐1 and PSF2 displayed as in (C).

**Figure EV2:**
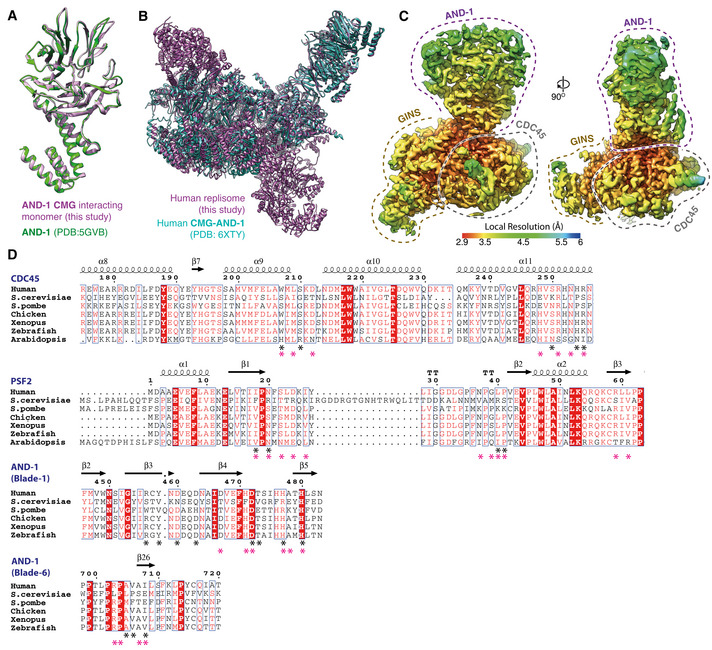
(Related to Fig 2). Structure of AND‐1 in the replisome Comparison of the AND‐1 SepB domain from the core replisome (this study, purple) with the crystal structure of the isolated protein (PDB: 5GVB, green) (Guan *et al*, 2017).Comparison of the positioning of AND‐1 in the core replisome (this study, purple) with the CMG:AND‐1 structure (PDB: 6XTY, cyan) (Rzechorzek *et al*, 2020).Multi‐body cryo‐EM map comprising AND‐1, CDC45 and GINS coloured by local resolution according to inset key.Multiple sequence alignments of residues involved in the AND‐1/CMG interface. Residues making specific AND‐1/CMG or Ctf4/CMG interface contacts are marked by a black (human) or pink (*S. cerevisiae*) asterisk. Alignments carried out using NCBI Clustal Omega (Sievers & Higgins, 2014) and visualised using ESPrit (Robert & Gouet, 2014) with the primary human sequence indicated. Uniprot ID for sequences used for CDC45 alignment: *H*. *sapiens* (O75419‐1), *S. cerevisiae* (Q08032‐1), *S. pombe* (O74113‐1), *G. gallus* (E1BYS7‐1), *X. laevis* (Q9YHZ6‐1), *D. rerio* (Q7ZU79‐1), *A. thaliana* (Q9LSG6‐1). PSF2 alignment: *H*. *sapiens* (Q03519‐1), *S. cerevisiae* (P40359‐1), *S. pombe* (O94329‐1), *G. gallus* (A0A1D5PQM4‐1), *X. laevis* (Q7ZT46‐1), *D. rerio* (Q4VBJ6‐1), *A. thaliana* (Q9C7A8‐1). AND‐1 alignment: *H*. *sapiens* (O75717‐1), *S. cerevisiae* (Q01454‐1), *S. pombe* (Q9C107‐1), *G. gallus* (P30985‐1), *X. laevis* (O13046‐1), *D. rerio* (A0A0R4J7S6‐1), *A. thaliana* (Q9ZQX6‐1).

AND‐1 comprises an N‐terminal WD40 domain, SepB domain and C‐terminal HMG‐box (Fig 2A), the latter being absent in yeast Ctf4. The SepB domain, that consists of a six‐bladed β‐propeller and C‐terminal bundle of five α‐helices, mediates AND‐1 trimerisation via the β‐propellers (Guan *et al*, 2017; Kilkenny *et al*, 2017). Similar to prior structures of the budding yeast replisome (Yuan *et al*, 2019; Baretić *et al*, 2020) and a lower resolution hsCMG:AND‐1 complex (Rzechorzek *et al*, 2020), we resolved ordered density only for a trimer of AND‐1 SepB domains, indicating the N‐terminal WD40 domains and HMG boxes are highly flexible. The SepB trimer is bound near‐perpendicular to the N‐tier face of CMG where it forms an extensive interface with CDC45 and the PSF2 subunit of GINS (Figs 1C and D, and 2B). Comparison of the SepB domain from the core human replisome with the crystal structure of the isolated domain revealed minimal structural differences (RMSD of 0.8 Å), indicating it docks onto CMG as a rigid body (Fig EV2A). AND‐1 is reported to interact directly with TIPIN (Errico *et al*, 2009). However, no cryo‐EM density is observed connecting the two proteins (Fig 1C). Furthermore, the positioning of the SepB domains is not influenced by the additional replisome proteins present in our structure relative to the hsCMG:AND‐1 complex (Fig EV2B).

Figure EV2C shows that the resolution of our cryo‐EM map at the AND‐1:CMG interface is 2.9–3.5 Å, which enabled unambiguous modelling of the majority of side‐chain rotamers in this region. The interface is formed by a single monomer of AND‐1 and buries 410 Å^2^ of PSF2 and 651 Å^2^ of CDC45 (Fig 2B). The AND‐1:CDC45 interface is mainly electrostatic and comprises blade 1 of the SepB β‐propeller, which sits across CDC45 α9 and α11 (Fig 2C). In contrast, the interface between PSF2 and AND‐1 is of a mixed electrostatic and hydrophobic nature and involves a loop connecting the β1 and β2 strands of PSF2 that projects into a cleft between blades 1 and 6 of the SepB β‐propeller (Fig 2D). The interaction involving blade 1 is primarily electrostatic, whereas the interaction involving blade 6 is largely hydrophobic (Fig 2D). While the residues involved in these interfaces are highly conserved in metazoa, there is weaker sequence conservation between *H. sapiens* and *S. cerevisiae* (Fig EV2D). However, despite the chemical nature of the interface differing between species, the spatial positioning of residues making contacts is well conserved (Fig EV2D), reflecting the similar arrangement of AND‐1 and Ctf4 in the human and budding yeast replisomes (Yuan *et al*, 2019; Baretić *et al*, 2020; Rzechorzek *et al*, 2020) (Fig EV1G).

### TIMELESS‐TIPIN structure

**Figure 3:**
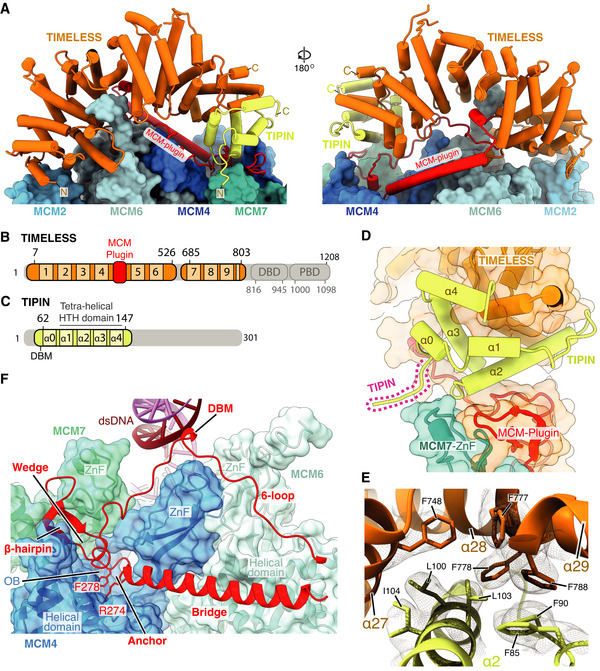
Structure of TIMELESS‐TIPIN in the human replisome Atomic model for TIMELESS‐TIPIN bound to MCM displayed using stubs and cylinders, with N‐ and C‐termini labelled. Models for the MCM2, 6, 4 and 7 subunits are displayed using surface rendering.Schematic for the domain architecture of TIMELESS. Regions of the protein visualised in this study are coloured, with domain boundaries and chain breaks demarcated with primary sequence numbering. The TIMELESS α‐solenoid helical repeats are numbered 1‐9. DBD–DNA‐binding domain, PDB–PARP1 binding domain.Schematic for the domain architecture of TIPIN displayed in the same manner as (B), with helices α0–4 indicated.Overview of TIPIN and its interfaces with TIMELESS and the MCM7 ZnF. TIPIN is displayed using cartoon cylinder rendering and TIMELESS and MCM7 are represented using both cartoon and transparent surface rendering.Detailed view of the hydrophobic interface formed between TIMELESS and α2 of TIPIN. Side chains of key residues are displayed and their primary sequence annotated. The cryo‐EM density for these selected residues is displayed as a transparent mesh.Overview of the TIMELESS MCM‐plugin interacting with the MCM2‐7 N‐tier. The MCM‐plugin is visualised using cartoon rendering and coloured red. MCM7, 4 and 6 are visualised using both cartoon and transparent surface rendering and are coloured according to subunit. The structural elements of the MCM‐plugin involved in binding to the N‐tier are labelled.

**Figure EV3:**
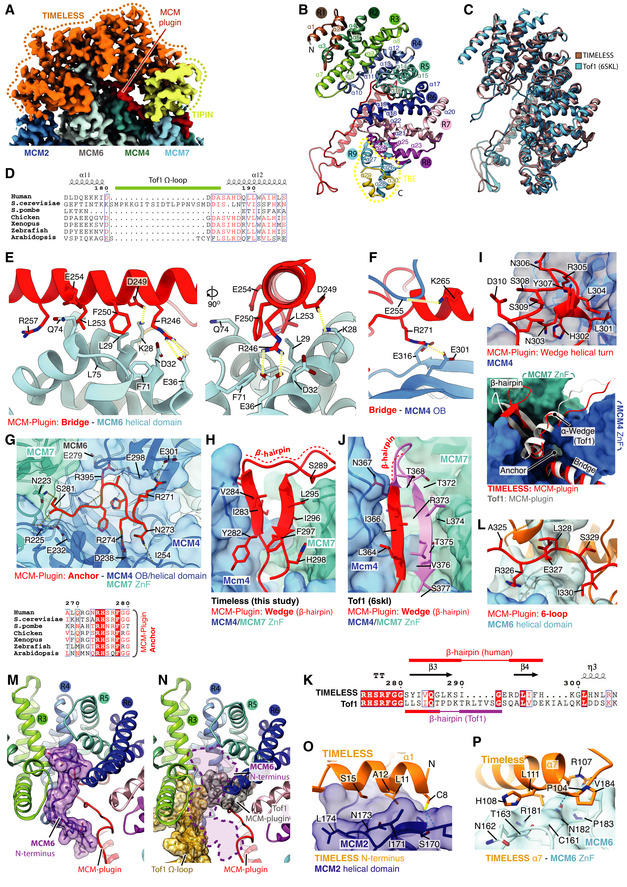
(Related to Fig 3). Structure of TIMELESS‐TIPIN ACryo‐EM density coloured according to protein chain occupancy, except for the TIMELESS “MCM‐plugin” element which is highlighted in red. For clarity DNA density is not shown.BAssignment of the TIMELESS helical repeats. Model is coloured according to helical repeat and the α helices numbered.CStructural comparison between TIMELESS from this study (brown) and yeast Tof1 (blue) (PDB: 6SKL) (Baretić *et al*, 2020).DMultiple sequence alignment region covering the Tof‐1 Ω‐loop. All alignments in the figure were carried out using NCBI Clustal Omega and visualised using ESPrit with the primary human sequence indicated. Uniprot ID for sequences used for TIMELESS alignments: *H*. *sapiens* (Q9UNS1‐1), *S. cerevisiae* (P53840‐1), *S. pombe* (Q9UUM2‐1), *G. gallus* (Q8QGQ6‐1), *X. laevis* (A0A6I8PXH0‐1), *D. rerio* (E7FGL0‐1), *A. thaliana* (A0A1P8B9S9‐1).E–J, LDetailed views of the interactions between the TIMELESS MCM‐Plugin and specific MCM subunits as indicated.G(Bottom) multiple sequence alignment covering residues that form the TIMELESS Anchor motif, alignment carried out as described in EV3D.KSequence alignment of Wedge from *H. sapiens* TIMELESS and *S. cerevisiae* Tof1 (PDB: 6SKL) (Baretić *et al*, 2020), alignment carried out as described in EV3D.MThe position of the MCM6 N‐terminus (purple), which occupies a cavity formed by helical repeats 3, 4 and 5 of TIMELESS and the MCM‐plugin. Models shown as cartoons with the MCM6 N‐terminus also displayed using transparent surface rendering.NThe cavity occupied by the MCM6 N‐terminus in the core human replisome is partially blocked by the Tof‐1 Ω‐loop and MCM‐plugin in *S. cerevisiae* (PDB: 6SKL) (Baretić *et al*, 2020). Model of TIMELESS, displayed as a cartoon, overlaid with the Tof‐1 Ω‐loop (gold) and Tof‐1 MCM‐plugin (grey) displayed as a cartoon with transparent surface rendering. The approximate position adopted by the MCM6 N‐terminus in the core human replisome is indicated with a dashed purple line.O, PDetailed views of the interactions between the TIMELESS α‐solenoid and MCM2 N‐terminal helical domain (O) and MCM6 zinc finger (P). TIMELESS model displayed using cartoon rendering with the surface of MCM2 and MCM6 displayed using transparent surface rendering.

TIMELESS‐TIPIN sits at the leading edge of the replisome in advance of MCM2, 6, 4 and 7 where it cradles the parental DNA duplex prior to strand separation (Figs 1C and D, and 3A). Well‐ordered density for approximately the first two‐thirds of TIMELESS (Fig EV3A) enabled modelling of residues 7‐803. This region adopts a right‐handed horseshoe‐shaped α‐solenoid comprising 9 helical repeats, the curvature of which mimics that of the MCM2‐7 ring (Figs 3A and B, and EV3B). In contrast, the C‐terminal ˜400 a.a. of TIMELESS, that contains DNA binding and PARP binding domains (DBD and PDB, respectively) (Lerner *et al*, 2020), is invisible and therefore not stably positioned in the core human replisome (Fig 1C). Similar behaviour was observed for the equivalent region of Tof1 in the budding yeast replisome (Baretić *et al*, 2020). TIPIN is located at the C‐terminal end of the TIMELESS α‐solenoid atop the N‐terminal domain of MCM7 (Fig 3A). We modelled ˜90 a.a. of TIPIN (residues 62–147) comprising a compact tetra‐helical helix‐turn‐helix domain (HTH) and a short DBM (Fig 3A and C). The HTH packs against the C‐terminal end of the α‐solenoid forming a hydrophobic interface involving TIPIN α‐helices 2–4 and TIMELESS α‐helices 27–29 (Fig 3B–E).

Like *S. cerevisiae* Tof1, human TIMELESS contains an MCM‐plugin (Fig 3A and F). However, in contrast to Tof1, the Ω‐loop is absent (Figs 3A and EV3C and D). The TIMELESS MCM‐plugin is ˜ 90 a.a and links helical repeats 4 and 5 (Figs 3A and EV3B). It contains four distinct structural features called the Bridge, Anchor, Wedge and MCM6‐interacting loop (6‐loop) that serve to attach the TIMELESS‐TIPIN complex to MCM (Fig 3F). The Bridge is a helix connecting the MCM6 and MCM4 N‐terminal domains. It is secured in place at one end by polar and hydrophobic contacts with the MCM6 helical domain (Fig EV3E), several of which are conserved in *S. cerevisiae*, and at the other end by the Anchor that sits in a depression on the surface of MCM4 between the OB‐fold and helical domain (Figs 3F and EV3F and G). The a.a. sequence of the Anchor is invariant between *S. cerevisiae* and human, emphasising its importance in TIMELESS‐TIPIN replisome attachment (Fig EV3G). After the Anchor, the Wedge sits between MCM4 and TIPIN and also contacts the MCM7 ZnF (Figs 3F and EV3H and I). Like *S. cerevisiae* Tof1 (Baretić *et al*, 2020), the TIMELESS Wedge is composed of a β‐hairpin and short helical turn. However, in contrast to Tof1, which contains a helix after the helical turn that contacts the MCM4 and MCM7 ZnFs, the TIMELESS Wedge has a stretch of random coil contacting these domains. Although the secondary structure and positioning of the Wedge in human TIMELESS are very similar to *S. cerevisia*e Tof1 (Baretić *et al*, 2020) (Fig EV3H and J), the a.a. sequence comprising the β‐hairpin differs significantly (Fig EV3K). This divergence in sequence but not structure underscores the importance of the Wedge β‐hairpin as a structural element in Tof1/TIMELESS proteins.

The TIMELESS MCM‐plugin then projects towards dsDNA, where it forms a DBM, and traces its way back to helical repeat 5 across the surface of MCM6, forming the 6‐loop (Figs 3F and EV3l). The architecture of Tof1 and TIMELESS differs significantly in this region because TIMELESS lacks the Ω‐loop. This enables the N‐terminus of MCM6 to extend into the core of TIMELESS, occupying a cavity formed by helical repeats 3–6 and the MCM‐plugin that is partially occupied by the Tof1 MCM‐plugin and Ω‐loop in the *S. cerevisiae* replisome (Baretić *et al*, 2020) (Figs 3A and EV3M and N). In addition, the N‐terminus of TIMELESS contacts the N‐terminal extension of MCM2 (Fig EV3O), while α7 of TIMELESS helical repeat 3 sits on top of the MCM6 ZnF (Fig EV3P). Together, these extensive and varied protein:protein interactions stably position TIMELESS‐TIPIN at the front of the human replisome.

### Pol ε structure and contacts with CMG

**Figure EV4:**
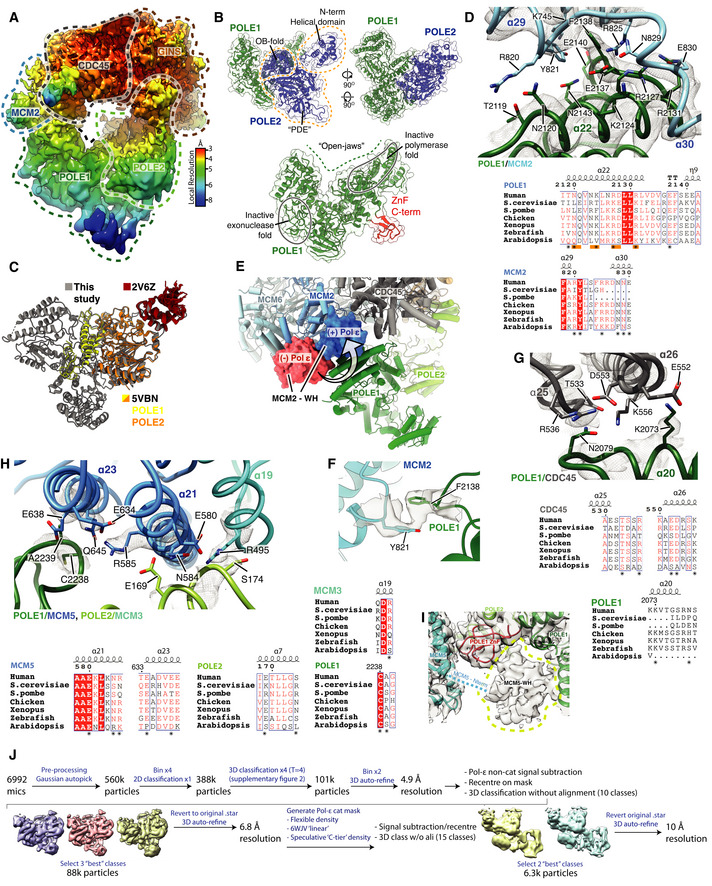
(Related to Fig 4). Structure of Pol ε Cryo‐EM map obtained using MultiBody refinement (Nakane *et al*, 2018) masking over the Pol ε non‐catalytic module, CDC45 and GINS. Map coloured by local resolution according to inset key.Model for the Pol ε^non‐cat^ domain as part of the core human replisome (other replisome components not shown). Models displayed as cartoons overlayed with transparent surface rendering. (Top) POLE1 is coloured green and POLE2 in blue. POLE2 domains are annotated and their position indicated using dashed lines. (Bottom) Model for POLE1 displaying the “wide‐open jaw” configuration. Approximate positions of the inactive polymerase and exonuclease motifs are indicated and the C‐terminal ZnF coloured red.Comparison between the structure of the Pol ε^non‐cat^ domain from this study (grey) with previous structures of POLE2 (orange) with a C‐terminal region of POLE1 (Baranovskiy *et al*, 2017) (yellow) (PDB: 5VBN) and the N‐terminal helical domain of POLE2 (red) (PDB: 2V6Z) (Nuutinen *et al*, 2008).(Top) Detailed view of the interface between POLE1 (green) and the MCM2 winged‐helix domain (blue). Atomic models visualised using cartoon rendering with selected side chains displayed and corresponding cryo‐EM density is overlaid in transparent mesh. (Bottom) Multiple sequence alignment for residues involved in the MCM2‐WH/POLE1 interface. Orange bars indicate the pattern of charge conserved residues along the same face of POLE1 α22, residues that make inter‐protein contacts are marked with an asterisk. All alignments in the figure were carried out using NCBI Clustal Omega and visualised using ESPrit with the primary human sequence indicated. Uniprot ID for sequences used for POLE1 alignment: *H*. *sapiens* (Q07864‐1), *S. cerevisiae* (P21951‐1), *S. pombe* (P87154‐1), *G. gallus* (E1C5P2‐1), *X. laevis* (A0A1L8HZP3‐1), *D. rerio* (B0V351‐1), *A. thaliana* (F4HW04‐1). MCM2 alignment: *H*. *sapiens* (P49736‐1), *S. cerevisiae* (P29469‐1), *S. pombe* (P40377‐1), *G. gallus* (F1NB20‐1), *X. laevis* (P55861‐1), *D. rerio* (A0A0R4IF65‐1), *A. thaliana* (Q9LPD9‐1).Model highlighting the repositioning of the MCM2 WH domain following Pol ε engagement with CMG. Replisome model displayed using cylinder and stub cartoon rendering. The position of the MCM2 WH domain is displayed using surface rendering in the absence of Pol ε (Rzechorzek *et al*, 2020) (red) and presence (blue).Model visualising the ring‐stacking interaction between MCM2 Y821 (blue) and POLE1 F2138 (green). Cryo‐EM density is displayed as a transparent surface.(Top) Detailed view of the interface between POLE1 and the CDC45 as shown as in (C). (Bottom) Multiple sequence alignment for residues involved in the POLE1/CDC45 interface. Residues that make inter‐protein contacts are marked with an asterisk. Uniprot ID for sequences used for POLE1 alignment is as described in (C) and Uniprot IDs used for the CDC45 alignment are as described in Fig EV2D.(Top) Detailed view of the interface involving POLE1, POLE2 and MCM5 as shown as in (C). (Bottom) Multiple sequence alignment for residues involved in the POLE1/POLE2/MCM5 interface. Residues that make inter‐protein contacts are marked with an asterisk. Uniprot ID for sequences used for POLE1 alignment are as described in panel b. Uniprot IDs used for POLE2 alignment: *H*. *sapiens* (P56282‐1), *S. cerevisiae* (P24482‐1), *S. pombe* (O94263‐1), *G. gallus* (Q5ZKQ6‐1), *X. laevis* (Q9DGB4‐1), *D. rerio* (Q8JHG8‐1), *A. thaliana* (Q500V9‐1). MCM5 alignment: *H*. *sapiens* (P33992‐1), *S. cerevisiae* (P29496‐1), *S. pombe* (P41389‐1), *G. gallus* (Q5ZKL0‐1), *X. laevis* (P55862‐1), *D. rerio* (F1QK71‐1), *A. thaliana* (O80786‐1).Low threshold cryo‐EM map, displayed as a transparent grey surface, for the complete core human replisome highlighting unmodelled density, in the same location as the MCM5 winged‐helix domain in previous *S. cerevisiae* structures, circled with a dashed yellow line.Schematic describing the processing pipeline used to obtain a cryo‐EM reconstruction containing additional ordered density for the Pol ε catalytic module.

**Figure 4:**
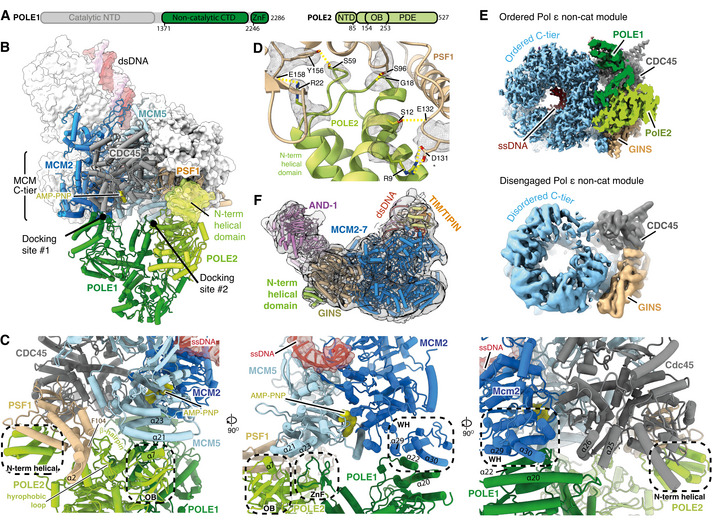
Structure and attachment of the Pol ε non‐cat module Primary structure diagram for POLE1 and POLE2. Regions of the protein visualised in this study are coloured and domain boundaries are demarcated using primary sequence numbering.Overview of the interactions between the Pol ε non‐cat module and hsCMG. POLE1, POLE2 and the hsCMG subunits with which they interact (MCM2, MCM5, PSF1 and CDC45) are coloured according to subunit and displayed using cartoon rendering. The N‐terminal helical domain of POLE2 that interacts with PSF1 is also shown as a transparent surface. The remaining hsCMG subunits are shown with light grey surface rendering and dsDNA is shown as a coloured surface. AND‐1 is not shown to aid visualisation of the interactions between the Pol ε non‐cat module and hsCMG.Expanded views of the interactions between the Pol ε non‐cat module and hsCMG.Detailed view of the interface between the POLE2 N‐terminal helical domain and PSF1. Cryo‐EM density is displayed as a grey mesh.Comparison of cryo‐EM maps where the Pol ε non‐cat module is either ordered or disengaged from the MCM C‐tier as indicated. See Fig EV4 for details of the cryo‐EM maps. Maps are coloured as in Fig 1C.Cryo‐EM map where the Pol ε non‐cat module is disengaged from the MCM C‐tier displayed as a transparent surface with the structure of the core human replisome, coloured as in Fig 1D, docked into the density. Clear density is observed for the POLE2 N‐terminal helical domain bound to PSF1.

We obtained a map of the non‐cat module of Pol ε at an average resolution of 6 Å (Fig EV4A) that enabled modelling of POLE1^nonCat^ (residues 1,371–2,280) and POLE2 (residues 1–527) (Fig 4A and B). This involved rigid‐body docking I‐TASSER homology models (see Materials and Methods, model building and refinement for details) (Yang *et al*, 2015) for both subunits followed by real‐space refinement using both Phenix (Afonine *et al*, 2018) and ISOLDE (Croll, 2018; Pettersen *et al*, 2021). In regions of the POLE1 map where the local resolution was insufficient to identify secondary structure features, primarily those most distal from hsCMG (Fig EV4A), the model was simply docked as a rigid body. Similar to yeast Pol2 (Goswami *et al*, 2018), POLE1^nonCat^ adopts a polymerase fold with a wide‐open jaw and C‐terminal zinc finger (Figs 4B and EV4B). POLE2 has a multidomain organisation with a flexibly tethered N‐terminal helical domain, followed by an OB domain and inactive calcineurin‐like phosphoesterase domain (PDE) that is responsible for the majority of the contacts between POLE2 and POLE1^nonCat^ (Figs 4A and B, and EV4B) (Baranovskiy *et al*, 2017). The conformation of the Pol ε^nonCat^ domain in the core human replisome shares a high degree of structural homology with the crystal structure of the POLE2‐POLE1_C‐term_ complex (PDB:5VBN, RMSD ‐ 1.21 Å) (Baranovskiy *et al*, 2017) and the NMR structure of the POLE2 N‐terminal helical domain (PDB:2V6Z ‐ 1.23 Å) (Fig EV4C) (Nuutinen *et al*, 2008).

We observed good local map resolution (3–5 Å) at the majority of interfaces between hsCMG and Pol ε (Fig EV4A), revealing how Pol ε is attached in the replisome. Pol ε forms two multi‐subunit interfaces with CMG, one involving MCM2 and CDC45 (docking site 1) and the other involving MCM5 and PSF1 (docking site 2) (Fig 4B). Docking site 1 is primarily composed of electrostatic contacts between POLE1 (α20 and α22) and the C‐terminal MCM2 winged‐helix (WH) (α29 and α30) (Fig 4C centre and right panels and Fig EV4D), a ring‐stacking interaction between the well‐conserved F2138 of POLE1 and invariant Y821 of MCM2 (Fig EV4F), and a small electrostatic interface between the C‐terminus of CDC45 and POLE1 α20 (Figs 4C and EV4G). To form docking site 1, the MCM2 WH is repositioned from its location in the hsCMG:ssDNA complex (Rzechorzek *et al*, 2020), where it sits at the base of the C‐tier, and the linker connecting the WH with the C‐terminal domain of MCM2 is remodelled (Fig EV4E). This is accompanied by a loss of density for the MCM6 WH, likely because the two WH domains interact in the hsCMG:ssDNA structure (Rzechorzek *et al*, 2020).

Docking site 2 consists of a number of smaller interfaces involving Pol ε, MCM5 and the GINS subunit PSF1 (Fig 4B and C left and centre panels and EV4H). Low‐resolution density is also observed between the POLE1 zinc finger and polymerase domains in an analogous position to the MCM5 WH in *S*. *cerevisiae* (Goswami *et al*, 2018) (Fig EV4I). Finally, the high resolution of our map permitted de novo building of an atomic model for the N‐terminal helical domain of POLE2 that docks into a surface exposed pocket of PSF1. This domain is connected via a flexible linker and attaches to CMG by forming an extensive network of hydrogen bonds with PSF1 (Fig 4D).

The prior structure of scCMG bound to Pol ε, fork DNA and ATP‐γ‐S indicated that only two ATPase sites, MCM2:5 and MCM5:3, were nucleotide bound (Goswami *et al*, 2018). This contrasted with structures obtained in the absence of Pol ε where the MCM6:2 ATPase site was also occupied (Georgescu *et al*, 2017), suggesting Pol ε could alter the configuration of MCM active sites (Goswami *et al*, 2018). While our structure has ssDNA bound on the opposite side of the MCM central pore compared to the structure of hsCMG:ssDNA (Rzechorzek *et al*, 2020), this could be due to the presence of any one of the four additional replisome components, or to differences in sample preparation. Therefore, to more directly examine if Pol ε might alter the configuration of the hsCMG C‐tier, we identified a 3D class lacking clear Pol ε density but containing the remaining replisome components. Strikingly, Fig 4E shows that this class displayed a loss of ssDNA engagement and considerable conformational flexibility in the C‐tier, especially for the MCM2 subunit. Upon closer inspection of the cryo‐EM map, well‐ordered density for the N‐terminal helical domain of POLE2 bound to PSF1 was visible (Fig 4F). Therefore, the changes in C‐tier configuration observed in this 3D class are not due to a lack of Pol ε, but might rather result from a failure of the Pol ε non‐cat module to correctly engage the MCM C‐tier. Nevertheless, although we cannot exclude the possibility that this configuration arose due to failed complex assembly, the data are consistent with Pol ε having the capacity to alter C‐tier configuration, which might enable Pol ε^non‐cat^ to modulate CMG helicase activity, as has been observed at protein barriers (Hizume *et al*, 2018). Notably, the data also indicate that Pol ε might retain CMG association via the POLE2 N‐terminal helical domain if the remainder of the protein were to detach from the MCM C‐tier at docking sites 1 and 2.

### Location of the Pol ε catalytic domain

**Figure 5:**
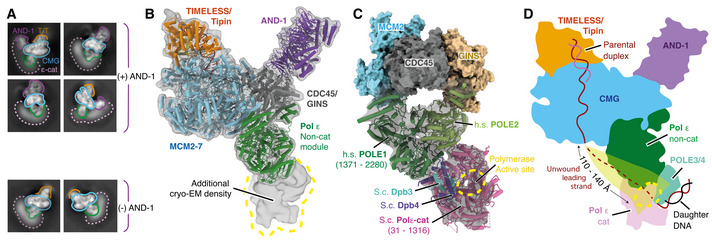
Position of the Pol ε catalytic domain in the replisome 2D classes for the core human replisome for particles containing and lacking AND‐1 density as indicated. Additional diffuse density that we attribute to the Pol ε catalytic domain is indicated (dotted pink line).Model for the core human replisome docked into a cryo‐EM map (transparent grey) displaying ordered density, continuous with the Pol ε non‐cat module, projecting away from CMG. Map obtained using extensive processing methods described in Fig EV4J.Model of the budding yeast (*S.c*.) Pol ε catalytic domain and Dpb3/4 (PDB: 6WJV) (Yuan *et al*, 2020b) rigid‐body‐docked into the unmodelled cryo‐EM density described in (B). Models for the human non‐catalytic and yeast catalytic modules are visualised using cylinders and stubs cartoon rendering with MCM2, CDC45 and GINS visualised using surface rendering.Illustration of the human replisome with Pol ε adopting the linear configuration. The putative path of the leading strand following extrusion from the C‐tier, to the POLE1 active site, is highlighted.

Although the catalytic domain of POLE1 and the small accessory subunits POLE3 and POLE4 were invisible in our highest resolution cryo‐EM map, diffuse density was observed in 2D class averages radiating in an arc from the Pol ε non‐cat module that we hypothesised was contributed by the Pol ε catalytic domain (Fig 5A). This is consistent with the flexible tethering of the Pol ε catalytic domain in the *S. cerevisiae* replisome (Zhou *et al*, 2017; Goswami *et al*, 2018). To further investigate the location of the Pol ε catalytic domain in the replisome, we used additional data processing (Fig EV4J) to recover a rare 3D class (1.4% of the total number of input particle images) with a large region of ordered density extending from the Pol ε non‐cat module (Fig 5B). Although the resolution of this density was too low for model building (12–16 Å), likely due to limited particle numbers, we could dock the structure of yeast Pol ε in the rigid linear configuration (Yuan *et al*, 2020b) into the density with good agreement (Fig 5C). Therefore, our data demonstrate that, not only can human Pol ε adopt this configuration, it can do so when incorporated into the replisome. Notably, this positions the active site of Pol ε 110–140 Å from the MCM central pore exit where the unwound leading‐strand template will emerge from the helicase (Fig 5D).

### CLASPIN binding in the human replisome

**Figure 6:**
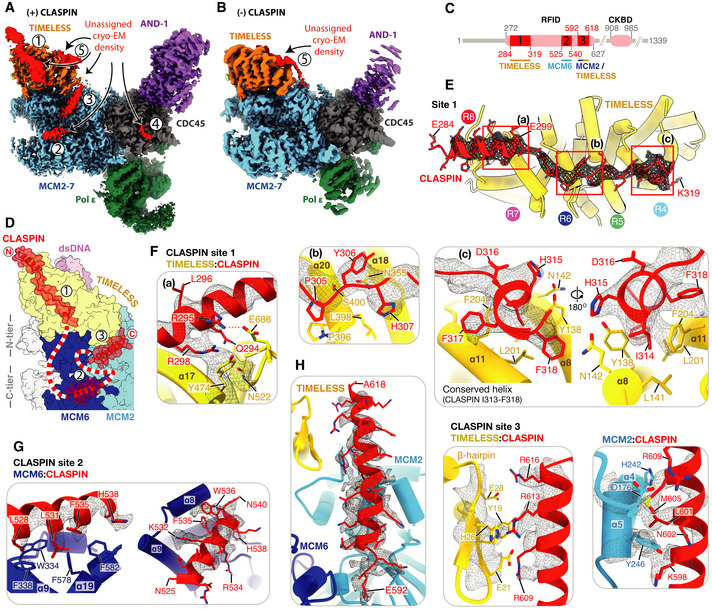
CLASPIN binding in the human replisome ACryo‐EM map of the complete core replisome coloured as in Fig 1C showing regions of density, labelled 1–5 (red), that remained unassigned after initial model building.BCryo‐EM map of a core replisome complex prepared in the absence of CLASPIN (Appendix Fig S3) coloured as in (A). Unassigned density 5 (red) is present in the absence of CLASPIN.CSchematic for the domain architecture of CLASPIN. Pink ovals represent previously characterised functional regions with primary sequence numbering in grey. RFID: replication‐fork interaction domain, CKBD: Chk1 binding domain. Red rectangles represent regions of CLASPIN visualised in this structure, with primary sequence numbering in red and replisome components that contact each respective region labelled below.DSchematic for the path of CLASPIN across one side of the replisome. Models visualised using surface rendering and coloured as in (A). CLASPIN model is displayed using surface transparent rendering with ribbon model overlaid. Red and white dashed line represents CLASPIN sequence not visualised linking sites 1–3.E–HDetailed views of the contacts between CLASPIN and replisome components. Models displayed using cartoon rendering overlaid with density for selected CLASPIN residues displayed using grey mesh. (E) Overview of CLASPIN site 1. TIMELESS helical repeats are indicated by coloured circles. (F) Detailed views of interactions at CLASPIN site 1. Left, a: CLASPIN residues E284‐E299 form an α‐helix that contacts TIMELESS helical repeats 7 and 8. CLASPIN R298 contacts TIMELESS Y474 with density displayed for both residues. Centre, b: CLASPIN residues P305‐H307 sit between TIMELESS helical repeats 5 and 6. Right, c: Two views of CLASPIN residues F318‐H315 docked into a hydrophobic pocket formed between TIMELESS helices α8 and α11. (G) CLASPIN site 2. Two views of CLASPIN residues N525‐N540 binding a hydrophobic patch formed by MCM6 α9 and α19 in the MCM C‐tier. (H) CLASPIN site 3. Three views of CLASPIN residues E592‐A618 interacting with both the TIMELESS N‐terminal β‐hairpin (centre) and MCM2 α4 and α5 (right).

**Figure EV5:**
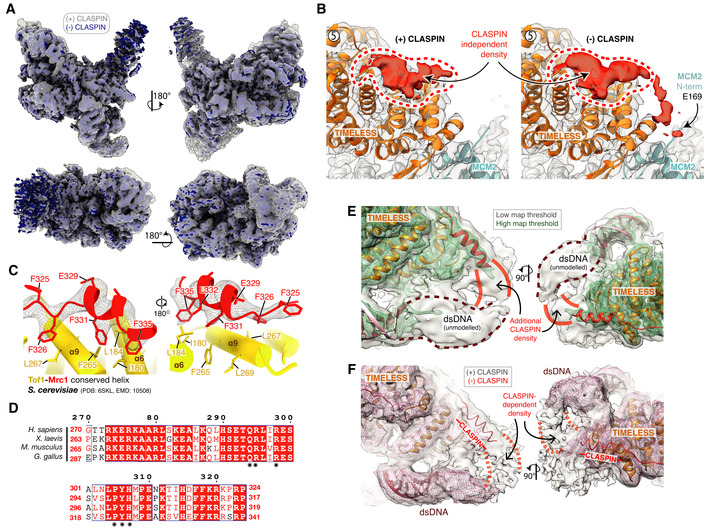
(Related to Fig 6). Identification of CLASPIN docking sites on the human replisome Cryo‐EM reconstruction of the core human replisome in the presence of CLASPIN (grey) rigid‐body‐docked into the cryo‐EM reconstruction in the absence of CLASPIN (blue) using UCSF Chimera (Pettersen *et al*, 2004) (correlation 0.8993).Detailed view of the CLASPIN independent density that is bound to the N‐terminal end of the TIMELESS α‐solenoid. (Left) density in the presence and (right) absence of CLASPIN.Closeup view of the interface between Mrc1 residues F335‐F325 and Tof1. The Mrc1 model was built into unmodelled density present in the *S. cerevisiae* replisome (PDB: 6SKL, EMD: 10508). Tof1 α6 and α9 are structurally equivalent to TIMELESS helices α8 and α11, respectively. Tof1 and Mrc1 are visualised using cartoon rendering with density assigned to Mrc1 displayed as grey mesh.Multiple sequence alignment for *H. sapiens* CLASPIN residues 270–324 at CLASPIN site 1. CLASPIN residues involved in contacting the replisome are marked with an asterisk. Alignments carried out using NCBI Clustal Omega (Sievers & Higgins, 2014) and visualised using ESPrit (Robert & Gouet, 2014) with the primary sequence indicated. Uniprot ID for sequences used for CLASPIN alignment: *H*. *sapiens* (Q9HAW4‐1), *M. musculus* (Q80YR7‐1), *G. gallus* (F1P0J7‐1), *X. laevis* (Q9DF50‐1).Cryo‐EM map for the core human replisome in the presence of CLASPIN at both high (green mesh) and low (grey transparent surface) thresholds, indicating the presence of density, continuous with CLASPIN site #1, projecting towards the upstream parental DNA duplex.Cryo‐EM map of the core human replisome in the presence of CLASPIN (grey transparent surface) and absence (red mesh) both at low thresholds. Indicates that density projecting from CLASPIN‐dependent site #1 towards the unmodelled parental DNA duplex is dependent upon CLASPIN.

Despite not observing significant cryo‐EM density that we could initially attribute to CLASPIN, five distinct regions of density remained unassigned following the completion of initial model building (Fig 6A). Although several of these regions were resolved to 3.2–4.5 Å (Fig 6A, regions 1–3) and displayed clear helical and side‐chain densities, amino acid sequence assignment was challenging due to the limited size and disconnected nature of the densities. We hypothesised that the densities might represent regions of CLASPIN because their positioning was reminiscent of similar unassigned densities in the yeast replisome that XL‐MS experiments suggested might be contributed by Mrc1 (Baretić *et al*, 2020). Therefore, to further examine the identity of the unassigned densities, we determined a cryo‐EM structure of the human replisome lacking CLASPIN to 3.4 Å resolution (Figs 6B and Appendix Fig S3). CLASPIN omission did not significantly alter the structure of the replisome (Fig EV5A). Despite this, four of the five regions of unassigned density were completely absent, strongly suggesting they represent regions of CLASPIN (compare Fig 6A and B). Region 5, which is bound to the N‐terminal end of the TIMELESS α‐solenoid, remained present in the absence of CLASPIN (Figs 6A and B, and EV5B). This surface of TIMELESS was previously proposed to be a protein:protein interaction site because it bound to an affinity purification tag in the crystal structure of *Chaetomium thermophilum* Tof1 (Grabarczyk, 2020). The same study showed that mutation of positively charged Tof1 residues in the area abolished interaction of Tof1‐Csm3 with a fragment of Mrc1. Given that our structures now show that a replisome protein other than CLASPIN/Mrc1 can bind to this surface of TIMELESS, the prior study may not have accurately recapitulated Tof1‐Csm3:Mrc1 interactions. Alternatively, these observations might indicate that CLASPIN/Mrc1 competes with another replisome protein for binding to this region of TIMELESS/Tof1.

Having established that the unassigned cryo‐EM densities 1‐4 were likely contributed by CLASPIN, we inspected the AlphaFold structure prediction for *H. sapiens* CLASPIN (Jumper *et al*, 2021; Tunyasuvunakool *et al*, 2021) to identify regions of the protein that were predicted to be helical. Interestingly, the majority of CLASPIN is predicted to be unstructured with only a limited number of regions confidently predicted to form α‐helices (pLDDT score 70–90). Therefore, we systematically inspected the fit to the CLASPIN‐dependent densities, of all regions predicted to be α‐helical and of appropriate length. This enabled docking of segments of the AlphaFold structure prediction into three of the CLASPIN‐dependent densities (sites #1–3) with high confidence. The density at site #4 was of insufficient resolution to permit docking. Following fit‐to‐density optimisation using real‐space‐refinement, we were able to generate an atomic model for CLASPIN residues 284–319 (site #1), residues 525–540 (site #2) and residues 592–618 (site #3) (Fig 6C and D). These assignments are consistent with cross‐linking mass spectrometry data of the budding yeast replisome (Baretić *et al*, 2020) that demonstrated Mrc1 residues 300 and 322 cross‐link to the leading edge of Tof1, while Mrc1 residues 425, 454 and 462 cross‐link to the C‐tier by Mcm6/Mcm2. Furthermore, upon reinspection of our published *S. cerevisiae* cryo‐EM map of the Tof1 N‐terminus (Baretić *et al*, 2020), we could unambiguously dock the AlphaFold structure prediction for Mrc1 residues 325–335 (Jumper *et al*, 2021) into the equivalent position to CLASPIN site #1 (Fig EV5C).

The first region of CLASPIN (residues 284–319) extends along the top of TIMELESS (Figs 6E and EV5D), where residues E284‐E299 form an α‐helix that sits astride repeats 7 and 8 of the TIMELESS α‐solenoid (Fig 6F, left a). At low map thresholds, density for the N‐terminus of this helix extends to contact the parental DNA duplex ahead of TIMELESS (Fig EV5E and F). CLASPIN residues S300‐T313 then snake through a groove between TIMELESS helical repeats 5 and 6 (Fig 6F, centre b) before residues I314‐F318 form a short α‐helix, highly conserved in metazoa, that nestles within a hydrophobic patch between α8 and α11 of TIMELESS (Fig 6F, right c). We note that this α‐helix forms part of a previously identified PCNA‐interacting protein (PIP) motif that has been shown to mediate the binding of CLASPIN to PCNA (Yang *et al*, 2016). Given that this region is bound to TIMELESS/Tof1 in both the *H. sapiens* and *S. cerevisiae* replisomes (Figs 6F and EV5C), further work is required to determine whether replisome‐associated CLASPIN can bind PCNA.

The second region of CLASPIN (residues N525‐N540) forms a short α‐helix that is cradled by α9 and α19 of MCM6 in the MCM2‐7 C‐tier (Fig 6G). CLASPIN residues L528, L531, F535 and H538 project into a hydrophobic patch on MCM6 formed by residues W334, F338, F578 and F582.

The third region of CLASPIN (residues E592‐A618) extends N to C from the base of the MCM2 N‐tier towards helical repeat 1 of TIMELESS (Fig 6H). The interface is of a primarily charged nature with a trio of arginine residues on CLASPIN (R616, R613 and R609) interacting with a beta‐turn at the TIMELESS N‐terminus (Fig 6H, centre), while CLASPIN residues M605, N602 and K598 form extensive contacts with α4 and α5 of MCM2 (Fig 6H, right).

Our structure shows that CLASPIN adopts an extended and flexible configuration stretching across one side of the replisome, from its N‐terminal association with TIMELESS, across MCM6 and MCM2, towards CDC45. Based on the stoichiometry of CLASPIN in glycerol gradients (Figs 1B and Appendix Fig S1C), we consider it likely that a single copy is present in the replisome. The arrangement of CLASPIN is very similar to the positioning of Mrc1 in the *S*. *cerevisiae* replisome that we determined by XL‐MS (Baretić *et al*, 2020), indicating that it is conserved amongst CLASPIN/Mrc1 proteins. Importantly, our data have directly identified CLASPIN binding sites in the human replisome, illustrating that the first 600 a.a. of CLASPIN contains three discrete structural elements for CLASPIN replisome attachment. Consistent with our assignment of site #1 on TIMELESS, the first 350 a.a. of CLASPIN are sufficient for its association with TIMELESS in 293T cells (Yang *et al*, 2016). Prior work in Xenopus egg extracts identified a replication‐fork interacting domain (RFID) that was essential for CLASPIN to stably associate with chromatin (Lee *et al*, 2005). Notably, the RFID is equivalent to human CLASPIN residues 272–627 and therefore encompasses the regions of CLASPIN at sites #1–3, illustrating that they are likely to be crucial for CLASPIN function at the replication fork. Accordingly, whereas N‐terminal fragments of CLASPIN containing the RFID could associate well with chromatin in Xenopus egg extracts that contained full‐length CLASPIN, deletion of CLASPIN residues at site #1, or the helix at site #3, greatly reduced association with chromatin (Lee *et al*, 2005). Similar results were observed when these regions were removed from full‐length CLASPIN, or when residues at site #1 were mutated to alanine (equivalent residues to human CLASPIN Q294, R295, L296, P305, Y306, H307 (Fig 6Fa and Fb)), indicating that both sites #1 and #3 contribute to CLASPIN replisome binding in Xenopus egg extracts. In contrast, when full‐length CLASPIN was depleted from egg extracts, CLASPIN constructs lacking the regions required for attachment at sites #1 and #3 could bind to chromatin (Lee *et al*, 2005). This suggests that, although both sites #1 and #3 contribute to CLASPIN chromatin association, individual sites are not essential.

Previous work identified DNA‐binding activity in the N‐terminus of *S. pombe* Mrc1 (a.a. 160–317) and *H. sapiens* CLASPIN (a.a. 149–340) (Sar *et al*, 2004; Zhao & Russell, 2004). Although this is consistent with the positioning of the CLASPIN N‐terminus in our structure, where it appears to contact the parental DNA duplex ahead of TIMELESS, both studies found that CLASPIN/Mrc1 bound preferentially to branched DNA structures rather than dsDNA. Furthermore, two lysine residues in *S. pombe* Mrc1 (K235, K236), that when mutated abolished DNA binding, are situated immediately ahead the short α‐helix that binds TIMELESS/Tof1 at site #1. These observations therefore indicate that DNA‐binding studies using isolated regions of CLASPIN may not accurately recapitulate CLASPIN DNA binding in the human replisome.

### Coordination of replication fork DNA in the human replisome

**Figure 7:**
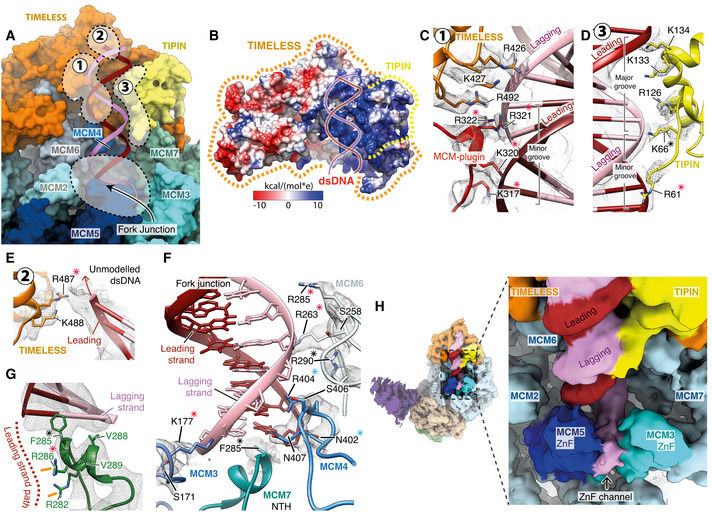
Coordination of replication fork DNA during template unwinding AOverview of the interactions between the MCM2‐7 N‐tier and TIMELESS‐TIPIN with the parental DNA duplex and fork junction. Key regions of protein–DNA contacts are circled with dashed lines and labelled. Surface rendering of the replisome model with DNA displayed as a cartoon.BSurface rendering of TIMELESS‐TIPIN coloured by Coulombic potential (see inset key) highlighting the positively charged concave groove that accommodates the parental DNA duplex. Approximate positions of TIMELESS and TIPIN indicated using dashed lines.C–EDetailed views of the contacts between TIMELESS‐TIPIN and the parental DNA duplex. Cartoon model rendering with selected side chains displayed and overlaid with their corresponding cryo‐EM density in transparent mesh. Asterisks indicate conserved residues, red—charge conserved, blue—highly conserved, black—invariant. (C) A network of positively charged residues from the TIMELESS MCM‐plugin and helical repeats 6‐7 interact with the DNA backbone across the minor groove. (D) Positively charged residues extending from the TIPIN tetra‐helical HTH contact the DNA backbone, an interface that is augmented by additional DNA contacts formed by the N‐terminal DNA‐binding motif of TIPIN. (E) Positively charged residues from helical repeat 7 of TIMELESS project towards the leading strand of the parental DNA duplex.F, GDetailed view of the replisome contacts with DNA at the fork junction. Asterisks indicate conserved residues, red—charge conserved, blue—highly conserved, black—invariant. (F) Contacts between MCM3,4,6 and 7 in the N‐tier and the DNA fork junction. (G) Detailed view of the MCM7 NTH positioned against the final base of dsDNA. The likely path of the leading‐strand template following unwinding is depicted by a dashed line and two arginine residues likely to coordinate it are highlighted (orange lines).H(Left) Cryo‐EM map for the core human replisome coloured according to chain occupancy using a radius of 5 Å. The cryo‐EM map was obtained using a subset of data processed in CryoSPARC (Fig EV6H). (Right) Zoomed in view of the fork junction displaying continuous density extending from the lagging‐strand template at the point of strand separation out through the MCM3/5 ZnF channel. Density in the MCM3/5 ZnF channel that we attribute to the lagging‐strand template was coloured manually in UCSF Chimera.

Ahead of strand separation, and at the fork junction, the human replisome makes extensive contacts with DNA involving TIMELESS‐TIPIN and four MCM subunits (Fig 7A). We observed strong cryo‐EM density in this region providing high‐resolution insights into forked DNA co‐ordination and the mechanism of strand separation. We speculate this is a result of TIMELESS‐TIPIN stabilising the parental DNA duplex, because, in the prior structure of hsCMG:ssDNA, although fork DNA was present in the sample, only ssDNA in the C‐tier was visible (Rzechorzek *et al*, 2020).

**Figure EV6:**
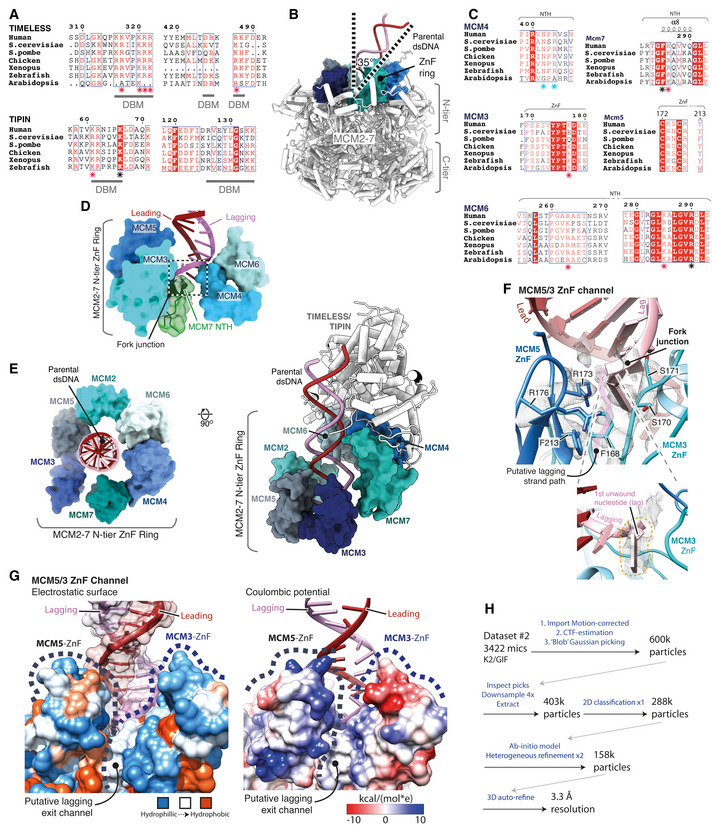
(Related to Fig 7). Interactions between the human replisome and fork DNA Multiple sequence alignment for regions in TIMELESS (top) and TIPIN (bottom) involved in DNA binding. Specific residues seen to be interacting with dsDNA in the structure are demarcated as part of a DNA‐binding motif (DBM). Level of conservation for residues contacting dsDNA indicated with a coloured asterisk, red—charge, blue—highly, black—invariant. All alignments in the figure were carried out using NCBI Clustal Omega and visualised using ESPrit with the primary human sequence indicated. Uniprot ID for sequences used for TIMELESS alignment: *H*. *sapiens* (Q9UNS1‐1), *S. cerevisiae* (P53840‐1), *S. pombe* (Q9UUM2‐1), *G. gallus* (A0A3Q2UKM8‐1), *X. laevis* (Q3LGB9‐1), *D. rerio* (E7FGL0‐1), *A. thaliana* (A0A1P8B9S9‐1). TIPIN alignment: *H*. *sapiens* (Q9BVW5‐1), *S. cerevisiae* (Q04659‐1), *S. pombe* (O14350‐1), *G. gallus* (Q5F416‐1), *X. laevis* (Q0IHI4‐1), *D. rerio* (Q6DBR4‐1).Side‐on view of the MCM2‐7 complex, displayed using pipes and planks, illustrating the angle of the parental dsDNA as it enters the N‐tier. The ring of ZnF domains that encircle the incoming duplex is rendered as an opaque surface.Multiple sequence alignment for the regions of MCM4, 6, 3, 5, 7 contacting the DNA at the fork junction as shown in (A). Level of conservation for residues contacting DNA at the fork junction indicated with a coloured asterisk, red—charge, blue—highly, black—invariant. Uniprot ID for sequences used for the MCM5 alignment is as described in Fig EV4H. Uniprot IDs used for MCM4 alignment: *H*. *sapiens* (P33991‐1), *S. cerevisiae* (P30665‐1), *S. pombe* (P29458‐1), *G. gallus* (E1C2U4‐1), *X. laevis* (P30664‐1), *D. rerio* (Q6NZV2‐1), *A. thaliana* (Q0WVF5‐1). MCM6 alignment: *H*. *sapiens* (Q14566‐1), *S. cerevisiae* (P53091‐1), *S. pombe* (P49731‐1), *G. gallus* (Q5ZKR8‐1), *X. laevis* (Q5FWY4‐1), *D. rerio* (A0A0E4AYA7‐1), *A. thaliana* (F4KAB8‐1). MCM7 alignment: *H*. *sapiens* (P33993‐1), *S. cerevisiae* (P38132‐1), *S. pombe* (O75001‐1), *X. laevis* (Q91876‐1), *D. rerio* (Q7ZVL6‐1), *A. thaliana* (P43299‐1). MCM3 alignment: *H*. *sapiens* (P25205‐1), *S. cerevisiae* (P24279‐1), *S. pombe* (P30666‐1), *X. laevis* (P49739‐1), *D. rerio* (A0A0E4AY38‐1), *A. thaliana* (Q9FL33‐1).MCM2‐7 N‐tier loops contacting the dsDNA at the fork junction. Model rendered as an opaque surface, with the MCM2‐7 NTH also displayed as a cartoon with transparent surface rendering.Two views of the MCM2‐7 N‐tier secondary ZnF domain ring, which encircles the incoming parental dsDNA duplex. The ZnF models are displayed using opaque surface rendering with TIMELESS‐TIPIN and dsDNA visualised as a cartoon using pipes and planks rendering.Detailed view of the putative lagging‐strand exit channel between the MCM3 and MCM5 ZnF’s (Top). Model visualised using cartoon rendering with selected side chains displayed and their corresponding cryo‐EM density represented as a transparent mesh. Focussed view highlighting the first lagging‐strand nucleotide following strand separation and its corresponding cryo‐EM density (Bottom).MCM5/3 ZnF channel displayed using surface rendering, coloured according to electrostatic potential (left) and Coulombic potential (right) (Pettersen *et al*, 2004) according to their respective inset keys.Schematic pipeline diagram describing the processing of 3422 micrographs from cryo‐EM collection #1 in the presence of CLASPIN using CryoSPARC (Punjani *et al*, 2017). This processing resulted in a reconstruction in which density was identified between the MCM5 and MCM3 ZnF’s that is continuous with the lagging strand at the fork junction.

TIMELESS and TIPIN form a positively charged, concave groove that grips a complete turn of dsDNA ahead of the fork junction (Fig 7B). The groove is lined by numerous arginine and lysine residues (Fig 7C–E), many of which are conserved (Fig EV6A). These basic residues are contributed by DBMs from the MCM‐plugin and TIPIN N‐terminus, similar to *S. cerevisiae* Tof1‐ Csm3 (Baretić *et al*, 2020), as well as additional residues from the TIMELESS α‐solenoid and the TIPIN tetra‐helical HTH. The DNA contacts are almost exclusively with the phosphate backbone, which should enable the dsDNA to rotate and slide across the surface of the groove as it is pulled towards the MCM N‐tier by the motor activity of CMG.

Approaching the point of strand separation, the dsDNA—that is tilted at about 35° from the vertical axis of the MCM pore (Fig EV6B)—is engaged by the N‐terminal hairpins of MCM6 and MCM4 that form an extensive network of contacts with the lagging‐strand template, often using conserved amino acids (Figs 7F and EV6C). These contacts, together with residues from the MCM3 ZnF, guide the DNA duplex onto the NTH of MCM7 that contains a short helix at its tip that is wedged between the two DNA strands (Fig EV6D). Here, an invariant phenylalanine (MCM7 F285) stacks against the final base pair in a manner characteristic of separation pins in diverse helicases (Velankar *et al*, 1999; Gao *et al*, 2019) (Fig 7F). The position of the MCM7 NTH, and therefore the point of strand separation, sits below the rim of a secondary N‐tier ring formed by the MCM ZnF domains (Figs 7A and EV6E). This arrangement of DNA contacts suggests an unwinding mechanism whereby, as the leading‐strand template is pulled through the MCM central pore by the C‐tier motor domains, the lagging‐strand template is blocked from entering the pore resulting in the two strands being forced apart. Although we observe only a single unpaired lagging‐strand nucleotide after strand separation (Fig EV6F), it is positioned at the mouth of a positively charged channel formed between the MCM3 and MCM5 ZnFs (Fig EV6G), strongly indicating that the unwound lagging‐strand template exits the secondary ZnF ring through this channel. Indeed, consistent with this hypothesis, processing of a subset of the cryo‐EM data in cryoSPARC (Punjani *et al*, 2020) (Fig EV6H) resulted in a reconstruction at lower global resolution but with clear density extending from the lagging‐strand template at the base of the DNA duplex though the putative MCM3/MCM5 ZnF exit channel (Fig 7H). Similarly positioned density was attributed to the lagging strand in cryo‐EM reconstructions of drosophila CMG unwinding DNA in the presence of ATP (Eickhoff *et al*, 2019).

## Discussion

We have determined the structure of an ˜1.8 MDa human replisome comprising the CMG replicative helicase, TIMELESS‐TIPIN, CLASPIN, AND‐1, Pol ε and fork DNA. Consistent with the essential function of the chromosome replication machinery, our structure shows that the overall architecture of the core eukaryotic replisome is extremely highly conserved, with human TIMELESS‐TIPIN, CLASPIN, AND‐1 and Pol ε all occupying equivalent positions to their *S. cerevisiae* counterparts (Goswami *et al*, 2018; Yuan *et al*, 2019; Baretić *et al*, 2020). This is consistent with human replisomes having evolved additional complexity primarily through the addition of new replisome components that modulate the function of the core replisome, rather than by altering the structure and properties of the core replisome itself. We anticipate our structure will form the basis of future investigations that directly examine the structural and mechanistic basis by which these additional replisome factors augment human replisome function.

The high resolution of our cryo‐EM map has enabled us to build an atomic model for the core human replisome that provides a wealth of information showing how five key replisome proteins are organised by CMG, the complex protein:protein interactions that underpin this organisation and how replication fork DNA is coordinated for template unwinding. The stable positioning of TIMELESS‐TIPIN at the front of the replisome enables it to grip dsDNA before unwinding and this is likely to be important for TIMELESS‐TIPIN‐dependent replication fork stabilisation and fork protection (Leman *et al*, 2010; Rageul *et al*, 2020), perhaps by stabilising the entire replisome on chromatin when its progression is perturbed. The C‐terminal ˜400 a.a. of TIMELESS contains a DBD and PBD. and promotes replication past G‐quadruplex structures (G4s) (Lerner *et al*, 2020). Because TIMELESS is positioned ahead of CMG, we consider it likely the DBD senses G4s either in dsDNA ahead of the fork junction or in the lagging‐strand template, rather than the leading‐strand template. Likewise, the DDX11 helicase, which contributes to G4 processing and sister‐chromatid cohesion, is presumably targeted to DNA at the front of the replisome via its interaction with TIMELESS (Cortone *et al*, 2018; Lerner *et al*, 2020). TIMELESS‐TIPIN is displaced from the replisome in response to redox changes to slow replication fork progression (Somyajit *et al*, 2017). It will be interesting to discover how the many protein:protein and protein:DNA interactions that attach TIMELESS‐TIPIN to the replisome are disrupted to induce its displacement.

CLASPIN/Mrc1 proteins are important for normal rates of DNA replication in yeast (Szyjka *et al*, 2005; Tourriere *et al*, 2005; Hodgson *et al*, 2007), and human cells (Petermann *et al*, 2008) and experiments with reconstituted yeast replisomes indicate that Mrc1 can enhance fork rate when either Pol ε or Pol δ is synthesising the leading strand (Yeeles *et al*, 2017), although how this is achieved is not known. Our discovery that CLASPIN appears to contact the parental DNA duplex directly, while also binding across both the MCM N‐ and C‐tiers (Figs 6D and EV5E), might enable it to influence replication fork rates by directly controlling CMG helicase activity, or perhaps by limiting CMG backtracking (Burnham *et al*, 2019) during replisome progression. Because CLASPIN/Mrc1 can also bind to Pol ε (Lou *et al*, 2008; Sercin & Kemp, 2011), we propose that simultaneously contacting elements at the front and back of the replisome enables CLASPIN to coordinate template unwinding with Pol ε‐mediated leading‐strand synthesis (Katou *et al*, 2003). Together with a need to accommodate conformational changes in CMG and Pol ε during replisome progression, the extended configuration of CLASPIN might necessitate the use of multiple binding sites for its attachment. Consistent with data from Xenopus egg extracts, that indicate the three CLASPIN binding sites we have discovered are important for CLASPIN chromatin association (Lee *et al*, 2005), disruption of site #1 by mutation or truncation of Mrc1 renders *S. pombe* cells sensitive to hydroxyurea, indicating site #1 is important for CLASPIN/Mrc1 function at the replication fork (Zhao & Russell, 2004). Interestingly, overexpression of CLASPIN and TIMELESS protects cancer cells from replication stress making them promising anti‐cancer targets (Bianco *et al*, 2019). Therefore, the insights afforded by our structure could potentially be exploited to generate inhibitors of CLASPIN and TIMELESS replisome association for anti‐cancer therapy.

Pol ε contacts hsCMG via multiple small interfaces. This is likely to be important to accommodate conformational changes in the helicase during replication, particularly in the C‐tier, without Pol ε dissociation. Indeed, our data indicate that the interaction between the POLE2 N‐terminal helical domain and PSF1 permits Pol ε to completely dissociate from MCM while retaining contact with hsCMG. This could be necessary during the bypass of roadblocks such as DNA–protein crosslinks (Sparks *et al*, 2019) and interstrand crosslinks (Huang *et al*, 2013) where the MCM ring presumably opens to allow transverse of the blockage. The disorder observed in the C‐tier that accompanied loss of Pol ε density (Fig 4E) indicates that Pol ε might have the capacity to modulate C‐tier configuration and therefore potentially CMG helicase activity. Further work is required to establish how Pol ε modifies helicase activity and how this could be augmented by Pol ε‐mediated leading‐strand synthesis that itself might be regulated by CLASPIN/Mrc1 (Yeeles *et al*, 2017). Although we show that the rigid linear conformation of Pol ε (Yuan *et al*, 2020b) can be accommodated in the human replisome, it is unclear whether it represents an active or paused state given the considerable distance between the polymerase active site and the emerging leading‐strand template. Nevertheless, the fact that this Pol ε configuration is conserved from budding yeast to human indicates it has an important role during chromosome replication. Structures of hsCMG:Pol ε performing leading‐strand synthesis are required to determine the active configuration of Pol ε in the replisome.

The complex network of protein:DNA interactions surrounding the fork junction determines that template unwinding occurs at a fixed position in the eukaryotic replisome. Consequently, the unwound lagging‐strand template will always be extruded in the same direction and all currently available data (Eickhoff *et al*, 2019; Baretić *et al*, 2020; Yuan *et al*, 2020a), including this work, indicate it exits the secondary N‐tier ring between the MCM3 and MCM5 ZnFs. We propose that precise positioning of the lagging‐strand template after unwinding is critical to coordinate downstream processes involving this template strand. These include nascent‐strand priming by Pol α, parental histone transfer and CMG ubiquitylation during replication termination, that was recently shown to be regulated by the presence of the lagging strand at the replication fork by an unknown mechanism (Deegan *et al*, 2020; Low *et al*, 2020; Vrtis *et al*, 2021).

Until now, knowledge of the complex protein:protein and protein:DNA interactions that underpin faithful genome duplication by the human replisome was largely limited to co‐immunoprecipitation experiments, biochemical and structural characterisation of sub‐complexes and inferences from replisome structures determined using model systems, principally *S. cerevisiae*. The structure of the core human replisome therefore represents a major step forward, that, in addition to providing high‐resolution insights into human replisome organisation and DNA unwinding mechanism, will serve as a powerful platform for the direct investigation of larger and more elaborate human replisome assemblies.

## Materials and Methods

### Expression plasmid construction

cDNAs encoding all subunits of hsCMG (MCM2, MCM3, MCM4, MCM5, MCM6, MCM7, PSF1, PSF2, PSF3, SLD5, CDC45), Pol ε (POLE1, POLE2, POLE3, POLE4), RFC1, RFC2, RFC3, RFC4, RFC5 TIMELESS‐TIPIN, AND‐1 and CLASPIN were codon‐optimised for overexpression in insect cells; PCNA was codon‐optimised for overexpression in *E*. *coli* and synthesised by GeneArt Gene Synthesis (ThermoFisher) (see Appendix Table S1 for isoform identifiers). CDC45 was encoded with an internal flag tag, whereas SLD5 and RFC1 contained an N‐terminal twin strep tag. For AND‐1 and CLASPIN, N‐ and C‐terminal 3X Flag tag were used, respectively. For TIMELESS‐TIPIN, TIMELESS was encoded with an N‐terminal twin strep tag along with TEV cleavage site (see Appendix Table S2 for affinity tag sequences). The codon‐optimised sequences were then cloned into a pACEBac1 vector separately. For expression of hsCMG, individual genes were amplified by PCR and expression cassettes encoding MCM2‐7 and GINS (PSF1, PSF2, PSF3 and SLD5) were generated in pBIG2ab and pBIG1a vectors, respectively, using a modified version of the BiGBac system (Weissmann *et al*, 2016). Similar to hsCMG, individual genes encoding Pol ε subunits were amplified and cloned into pBIG1a. For RFC, TIMELESS‐TIPIN, CLASPIN, and AND‐1, PaCEBac1 constructs were used in subsequent virus generation. See Appendix Table S3 for details of expression plasmids used.

### Protein expression

To prepare baculoviruses, vector constructs for each individual protein and complexes were transformed into EMBacY *E. coli* competent cells for bacmid generation. Isolated bacmid was then transfected into Sf9 cells using FuGENE® HD (Promega). These baculoviruses were then amplified before a large‐scale culture was infected. For expression of hsCMG, separate viruses expressing MCM2‐7, GINS and CDC45 were used to co‐infect 3 l of Hi5 cells at a density of 1 × 10^6^ cells/ml. Individual viruses expressing the four subunit Pol ε and RFC were used to infect 2 l of Hi5 cells. For CLASPIN, TIMELESS‐TIPIN and AND‐1, 1 l of Hi5 cells was infected with the viruses. Cell growth and viability were monitored and cells harvested upon growth arrest (normally on day 3 after infection).

### hsCMG purification

Cells from a 3‐l culture were resuspended in lysis buffer (40 mM Hepes‐NaOH pH 7.5, 10% glycerol, 0.005% Tween‐20, 0.5 mM TCEP, 150 mM NaOAc) + protease inhibitors (cOmplete, EDTA‐ free (Roche), one tablet per 50 ml buffer). Cells were lysed by dounce homogenisation and insoluble material was removed by ultracentrifugation (235,000 *g*, 4°C, 45 min). Flag M2 affinity gel (Sigma) (5 ml) was added to the lysate and incubated for 2 h at 4°C. Resin was collected in 20‐ml columns (Bio‐Rad) (2 ml bed volume per column) and washed with 100 ml lysis buffer per column. Proteins were eluted with 1 CV (column volume) buffer + 0.5 mg/ml 3× FLAG peptide (Sigma) and 2 CV buffer + 0.2 mg/ml 3× FLAG peptide. Elutions were pooled, 0.5 ml strep‐tactin XT superflow high capacity (iba) was added, and the sample was incubated for 40 min at 4°C. Resin was collected in 20‐ml column and washed with 10 CV buffer. Resin was further washed with 10 CV lysis buffer + 5 mM Mg(OAc)_2_ + 0.5 mM ATP followed by 30 CV wash without ATP and Mg(OAc)_2_. Proteins were eluted with 14 CV (0.5 ml each fraction) lysis buffer + 30 mM biotin. Fractions were pooled and applied to a MonoQ PC 1.6/5 (GE Healthcare) equilibrated in 25 mM Tris–HCl pH 7.2,10% glycerol, 0.005% Tween‐20, 0.5 mM TCEP, 150 mM KCl. CMG was eluted with a 30 CV gradient from 150 to 1,000 mM KCl, and peak fractions were dialysed overnight against 500 ml dialysis buffer (40 mM HEPES‐KOH pH 7.6, 80 mM KOAc, 2 mM Mg(OAc)_2_, 0.25 mM EDTA, 1 mM DTT, 10% glycerol). Protein was concentrated (Amicon Ultra, Ultracel ‐ 30K), frozen in liquid nitrogen and stored at −80°C.

### CLASPIN purification

Cells from a 1‐l culture were resuspended in lysis buffer (50 mM Tris–HCl pH 8, 10% glycerol, 0.005% Tween‐20, 0.5 mM TCEP, 400 mM NaCl) + protease inhibitors (cOmplete, EDTA‐free, one tablet per 50 ml buffer). Cells were lysed by dounce homogenisation, and insoluble material was removed by centrifugation (235,000 *g*, 4°C, 45 min). Flag M2 affinity gel (1 ml) was added and the lysate incubated for 2 h at 4°C. Resin was collected in 20‐ml column (2 ml bed volume) and was washed with 30 ml lysis buffer. Resin was further washed with 10 CV lysis buffer + 5 mM Mg(OAc)_2_ + 0.5 mM ATP, followed by 10 CV wash without ATP and Mg(OAc)_2_. CLASPIN was eluted in 1 CV lysis buffer + 0.4 mg/ml 3× FLAG peptide and 2 CV lysis buffer + 0.2 mg/ml 3× FLAG peptide. Eluates were pooled and 0.4 ml was applied to a Superose 6 10/300 (GE Healthcare) column equilibrated in 25 mM Tris–HCl pH 7.2, 10% glycerol, 0.005% Tween‐20, 0.5 mM TCEP, 150 mM NaCl. Peak fractions were pooled, frozen in liquid nitrogen and stored at −80°C.

### TIMELESS‐TIPIN purification

Cell pellet from a 1‐l culture was resuspended in lysis buffer (25 mM Hepes‐KOH pH 7.2, 150 mM KCl, 5% glycerol, 0.5 mM TCEP, 0.01% NP‐40‐S) + protease inhibitors (cOmplete, EDTA‐free, one tablet per 50 ml buffer). Cells were lysed by Dounce homogenisation, and insoluble material was removed by centrifugation (235,000 *g*, 4°C, 45 min). 0.5 ml Strep‐Tactin XT superflow high capacity was added to the lysate and incubated for 30 min at 4°C. Resin was collected in 20‐ml column (2 ml bed volume) and was washed 50 ml lysis buffer. Protein was eluted with 10 CV (0.5 ml each fraction) lysis buffer + 30 mM biotin. Fractions were pooled and applied to 1 ml HiTrap Q HP column (GE Healthcare) equilibrated in 25 mM Hepes‐KOH pH 7.2, 150 mM KCl, 5% glycerol, 0.5 mM TCEP, 0.01% NP‐40‐S. TIMELESS‐TIPIN was eluted with a 20 CV gradient from 150 to 1,000 mM KCl. Peak fractions were pooled, concentrated to ˜500 μl in an Amicon Ultra‐15 30 kDa MWCO concentrator and applied to a Superdex 200 Increase 10/300 gel filtration column (GE Healthcare) equilibrated in 25 mM Tris–HCl pH 7.2, 5% glycerol, 0.01% NP‐40‐S, 1 mM DTT, 150 mM NaCl. Peak fractions were pooled, frozen in liquid nitrogen and stored at −80°C.

### AND‐1 purification

Cell pellet obtained from 1 l of insect culture was resuspended in lysis buffer (25 mM Tris–HCl pH 7.2, 10% glycerol, 0.005% Tween‐20, 0.5 mM TCEP, 300 mM NaCl) + protease inhibitors (cOmplete, EDTA‐free, one tablet per 50 ml buffer). Cells were lysed by Dounce homogenisation, and insoluble material was removed by centrifugation (235,000 *g*, 4°C, 45 min). Flag M2 affinity gel (1 ml) was added to the lysate and incubated for 2 h at 4°C. Resin was collected in 20‐mL column (2 ml bed volume) and was washed with 30 ml lysis buffer. Resin was further washed with 10 CV lysis buffer + 5 mM Mg(OAc)_2_ + 0.5 mM ATP, followed by 10 CV wash without ATP and Mg(OAc)_2_. AND‐1 was eluted in 1 CV lysis buffer + 0.4 mg/ml 3× FLAG peptide and 2 CV buffer + 0.2 mg/ml 3× FLAG peptide. Eluates were pooled and applied to 1 mL MonoQ column equilibrated in 25 mM Tris–HCl pH 7.2, 10% glycerol, 0.005% Tween‐20, 0.5 mM TCEP, 150 mM NaCl). AND‐1 was eluted with a 20 CV gradient from 150 to 1,000 mM NaCl. Peak fractions were pooled, concentrated to ˜500 μl in an Amicon Ultra‐15 30 kDa MWCO concentrator and applied to a Superdex 200 Increase 10/300 gel filtration column equilibrated in 5 mM Tris–HCl pH 7.2, 10% glycerol, 0.005% Tween‐20, 0.5 mM TCEP, 150 mM NaCl. Peak fractions were pooled, frozen in liquid nitrogen and stored at −80°C.

### Pol ε purification

Cell pellet obtained from 2 l of insect cell culture was resuspended in lysis buffer (45 mM Hepes‐KOH pH 7.6, 100 mM NaCl, 10% glycerol, 0.5 mM TCEP, 0.02% NP‐40‐S) + protease inhibitors (cOmplete, EDTA‐free, one tablet per 50 ml buffer). Cells were lysed by dounce homogenisation, and insoluble material was removed by centrifugation (235,000 *g*, 4°C, 45 min). CaCl_2_ was added (2 mM) to the supernatant together with 2 ml Calmodulin Affinity Resin and incubated for 90 min at 4°C. Unbound protein was applied to 1 ml HiTrap Heparin column (GE Healthcare) equilibrated in 45 mM Hepes‐KOH pH 7.6, 100 mM NaCl, 10% glycerol, 0.5 mM TCEP, 0.02% NP‐40‐S. The protein was eluted with a 30CV gradient from 100 to 1,000 mM NaCl. Peak fractions were pooled and incubated with 1 ml Calmodulin Affinity Resin + 2 mM CaCl_2_ for 1 h. After incubation, resin was collected and washed with 50 ml lysis buffer + 2 mM CaCl_2_ and bound proteins were eluted with 10 CV (1 ml each fraction) lysis buffer + 2 mM EDTA + 2 mM EGTA. Fractions were pooled and applied to MonoQ PC 1.6/5 (GE Healthcare) column equilibrated in lysis buffer. The protein was eluted with a 30CV gradient from 100 to 600 mM NaCl. Peak fractions were pooled and dialysed overnight against 1 l dialysis buffer (25 mM HEPES‐KOH pH 7.6, 10% glycerol, 1 mM DTT, 0.005% Tween, 10% glycerol, 300 mM KOAc). Protein was concentrated with Amicon Ultra‐15 30 kDa MWCO concentrator, frozen in liquid nitrogen and kept at −80°C.

### RFC purification

Cell pellet obtained from 2 l of insect cell culture was resuspended in lysis buffer (25 mM Hepes‐KOH pH 7.6, 300 mM NaCl, 10% glycerol, 0.5 mM TCEP, 0.02% NP‐40‐S) + protease inhibitors (cOmplete, EDTA‐free, one tablet per 50 ml buffer). Cells were lysed by dounce homogenisation, and insoluble material was removed by centrifugation (235,000 *g*, 4°C, 45 min). 1 ml Strep‐Tactin XT superflow high capacity resin was added to the lysate and incubated for 30 min at 4°C. Resin was collected in 20‐ml column (2 ml bed volume) and was washed with 100 ml lysis buffer. Protein was eluted with 10 CV (1 ml each fraction) lysis buffer + 30 mM biotin. Fractions were pooled and applied to 1 ml HiTrap Heparin column (GE Healthcare) equilibrated in 25 mM Hepes‐KOH pH 7.6, 100 mM NaCl, 10% glycerol, 0.5 mM TCEP, 0.01% NP‐40‐S. The protein was eluted with a 30 CV gradient from 100 to 1,000 mM NaCl. Peak fractions were pooled and the conductivity of the sample was adjusted to a buffer containing 25 mM Hepes‐KOH pH 7.6, 100 mM NaCl, 10% glycerol, 0.5 mM TCEP, 0.01% NP‐40‐S. The sample was applied to 1 ml MonoQ column equilibrated in 25 mM Hepes‐KOH pH 7.6, 100 mM NaCl, 10% glycerol, 0.5 mM TCEP, 0.01% NP‐40‐S. Protein was eluted with a 20 CV gradient from 150 to 1,000 mM NaCl. Peak fractions were pooled and dialysed overnight against 1 l dialysis buffer (25 mM HEPES‐KOH pH 7.6, 10% glycerol, 1 mM DTT, 0.005% Tween, 10% glycerol, 300 mM KOAc). Protein was concentrated with Amicon Ultra‐15 30 kDa MWCO concentrator, frozen in liquid nitrogen and kept at −80°C.

### PCNA purification

Bl21 (DE3) Rosetta, transformed with pET28a PCNA (1 l culture), were grown at 37°C in LB + 50 μg/ml kanamycin + 10 μg/ml chloramphenicol to an OD_600_ of 0.6. Expression was induced by addition of 0.8 mM IPTG, and the culture was grown further for 3 h. Cells were harvested and the pellet was resuspended in 50 mM Tris–HCl pH 7.2, 10% w/v sucrose + protease inhibitors (cOmplete, EDTA‐free, one tablet per 50 ml buffer). Cells were lysed via sonication (30%, 5s on/5s off, total 2 min), and insoluble material was removed by centrifugation (235,000 *g*, 4°C, 20 min). Ammonium sulphate was added slowly to 150 mM, and then, polymin P was added to 0.4%. The sample was stirred for 10 min at 4°C and insoluble material was removed by centrifugation (27,000 *g* 4°C, 10 min). 0.23 g/ml solid ammonium sulphate was added slowly to the supernatant; the sample was stirred for 10 min and centrifuged (48,000 *g*, 4°C, 10 min). The pellet was resuspended in 3 ml 25 mM Tris–HCl pH 7.2, 10% glycerol, 1 mM EDTA and 100 mM NaCl and the sample dialysed against the same buffer for 2 h. The conductivity was adjusted to a buffer containing 25 mM Tris−HCl pH 7.2, 10% glycerol, 1 mM EDTA and 150 mM NaCl. The sample was applied to a 1 ml HiTrap SP FF and a 1 ml HiTrap heparin column assembled in tandem. The unbound sample was collected and applied to a 1 ml DEAE column equilibrated in 25 mM Tris–HCl pH 7.2, 10% glycerol, 1 mM EDTA and 150 mM NaCl. Protein was eluted with a 30 CV gradient from 150 to 600 mM NaCl. Fractions were pooled, diluted twofold in 25 mM Tris–HCl pH 7.2, 10% glycerol, 1 mM EDTA and 150 mM NaCl and applied to a 1 ml MonoQ column equilibrated in the same dilution buffer. Protein was eluted with a 30 CV gradient from 150 to 600 mM NaCl. Peak fractions were pooled, concentrated to ˜400 μl and applied to Superdex 200 increase 10/300 column (GE Healthcare) equilibrated in 25 mM Tris–HCl pH 7.2, 10% glycerol, 1 mM EDTA and 150 mM NaCl. Fractions were pooled; protein was concentrated with Amicon Ultra‐15 30 kDa MWCO concentrator, frozen in liquid nitrogen and kept at −80°C.

### RPA purification

Bl21 (DE3) Rosetta, transformed with pET28a RPA (1 l culture), were grown at 37°C in LB + 50 μg/ml kanamycin + 10 μg/ml chloramphenicol to an OD_600_ of 0.6. Expression was induced by addition of 0.3 mM IPTG, and the culture was grown further for 3 h. Cells were harvested, and the pellet was resuspended in 50 mM Tris–HCl pH 7.5, 10% glycerol, 100 mM KCl, 1 mM EDTA, 1 mM DTT, 0.01% NP‐40‐S + protease inhibitors (cOmplete, EDTA‐free, one tablet per 50 ml buffer). Triton X‐100 was added to 0.1% and stirred for 5 min at 4°C. Cells were lysed via sonication (30%, 5s on/5s off, total 2 min), and insoluble material was removed by centrifugation (235,000 *g*, 4°C, 45 min). The supernatant was applied to a 5 ml HiTrap Blue column equilibrated in 50 mM Tris–HCl pH 7.5, 10% glycerol, 100 mM KCl, 1 mM EDTA, 1 mM DTT, 0.01% NP‐40‐S. The column was washed first with 40 ml equilibration buffer and further washed with 40 ml 20 mM Tris–HCl pH 7.5, 10% glycerol, 0.8 M NaCl, 1 mM EDTA, 1 mM DTT, 0.01% NP‐40‐S. Protein was eluted in a buffer containing 40% ethylene glycol, 2.5 M NaCl, 10% glycerol, 20 mM Tris–HCl pH 7.5, 1 mM EDTA, 1 mM DTT, 0.01% NP‐40‐S. Peak fractions containing RPA were pooled and dialysed against 2 l dialysis buffer (20 mM Tris–HCl pH 7.5, 10% glycerol, 50 mM NaCl, 1 mM EDTA, 1 mM DTT, 0.01% NP‐40‐S) for 90 min. Protein was applied to a 2 ml Bio‐Gel HT hydroxyapatite column (Bio‐Rad), and the flow through was re‐applied once. The column was first washed with 6 ml 50 mM Tris–HCl pH 7.5, 10% glycerol, 100 mM KCl, 1 mM EDTA, 1 mM DTT, 0.01% NP‐40‐S; then, protein was eluted with 6 ml 50 mM Tris–HCl pH 7.5, 10% glycerol, 100 mM KCl, 1 mM EDTA, 1 mM DTT, 0.01% NP‐40‐S, 80 mM potassium phosphate. Peak fractions were pooled and diluted in 20 mM Tris–HCl pH 7.5, 10% glycerol, 0.1 M KCl, 1 mM EDTA, 0.01% NP‐40‐S, 0.5 mM TCEP to reduce the conductivity of the sample. The sample was then applied to 1 ml MonoQ (GE Healthcare) equilibrated in 20 mM Tris–HCl pH 7.5, 10% glycerol, 0.1 M KCl, 1 mM EDTA, 0.01% NP‐40‐S, 0.5 mM TCEP. Protein was eluted with a 15 CV gradient from 100‐550 mM KCl. Fractions enriched for RPA were pooled and dialysed against 2 l dialysis buffer (25 mM HEPES‐KOH pH 7.6, 150 mM KOAc, 0.5 mM TCEP, 10% glycerol, 0.02% NP‐40‐S) for 4 h. The protein was frozen in liquid nitrogen and kept at −80°C.

### DNA fork preparation

Stock solutions of both leading‐ and lagging‐strand oligos (Integrated DNA Technologies) were prepared, both at 53 μM in 25 mM HEPES‐NaOH, pH 7.5, 150 mM NaOAc, 0.5 mM TCEP, 2 mM Mg(OAc)_2_. The sequence of the lead strand fork was:

5′‐(Cy3)TAGAGTAGGAAGTGA(Biotinylated‐dT)GGTAAGTGATTAGAGAATTGGAGAGTGTG(T)_34_ T^∗^T^∗^T^∗^T^∗^T^∗^T, where * denotes a phosphorothioate backbone linkage. The sequence of the lagging‐strand fork was:

5′‐GGCAGGCAGGCAGGCACACACTCTCCAATTCTCTAATCACTTACCA(Biotinylated‐dT)CACTTCCTACTCTA.

Both leading and lagging oligos were mixed at an equimolar ratio and annealed to form a fork structure via gradual cooling from 80°C to room temperature.

### Preparation of fork DNA for helicase assay

To anneal fork DNA, equal molars of fork‐leading and fork‐lagging oligos were mixed. The mixture was heated to 75°C and cooled to room temperature gradually. Oligo stocks were prepared in 25 mM HEPES‐NaOH, pH 7.5, 150 mM NaOAc, 0.5 mM TCEP, 2 mM Mg(OAc)_2_. Oligo sequences were modified from the fork substrates used in previous work (Georgescu *et al*, 2017; Kose *et al*, 2020). Fork leading was 5′‐ (Cy3)TAGAGTAGGAAGTGA(Bio‐dT)GGTAAGTGATTAGAGAATTGGAGAGTGTG (T)_34_ T^∗^T^∗^T^∗^T^∗^T^∗^T, where ^∗^denotes phosphorothioate backbone linkages. Fork‐lagging was 5′‐(Cy5)GGCAGGCAGGCAGGCAGGCAGGCAGGCAGGCAGGCAGGCAACACACTCTCCAATTCTCTAATCACTTACCATCACTTCCTACTCTA. The sequence of trap oligo used in the assay to prevent re‐annealing of the unwound DNA was 5′‐GGCAGGCAGGCAGGCACACACTCTCCAATTCTCTAATCACTTACCA(Bio‐dT) CACTTCCTACTCTA.

### Helicase assay

To load CMG onto the substrate without unwinding, 50 nM CMG was incubated with 2 nM fork DNA in a buffer containing 25 mM HEPES‐KOH (pH 7.6), 100 mM potassium glutamate, 50 mM magnesium acetate, 0.005% (v/v) Tween‐20, 1 mM TCEP, 200 µg/ml BSA, 0.1 mM AMP‐PNP for 5 min at 37°C. 5 mM ATP (or equal volume of water in ‐ATP reaction) and 80 nM trap oligo were added to initiate the reactions. The reactions were stopped after 15‐min incubation at 37°C with a buffer containing 0.1% SDS, 10 mM EDTA, 5% glycerol, 10 U/ml Proteinase K and bromophenol blue. The reactions were run on 10% TBE PAGEr Gold Precast Gels (Lonza) at 170 V for 70 min. The gel was imaged on Typhoon laser imager (GE Healthcare).

### Primer extension assay

Primed template was prepared by annealing 500 nM oligonucleotide (sequence: 5′‐GAATAATGGAAGGGTTAGAACCTACCAT) to 50 nM M13mp18 ssDNA (New England Biolabs) in 10 mM Tris–HCl pH 7.6, 100 mM NaCl and 5 mM EDTA. The mixture was heated to 75°C and gradually cooled to room temperature. Unannealed oligonucleotide was removed using S400 column (GE Healthcare). The primer extension reaction was performed at 37°C in a buffer containing 25 mM HEPES‐KOH (pH 7.6), 100 mM potassium glutamate, 0.01% NP‐40‐S, 1 mM DTT, 10 mM Mg(OAc)_2_, 0.1 mg/ml BSA, 3 mM ATP, 400 μM CTP, GTP, UTP, 30 μM dATP, dCTP, dGTP, dTTP, 33 nM α‐[^32^P]‐dCTP. 1 nM primed templated was pre‐incubated with 250 nM RPA for 5 min. 20 nM PCNA and 4 nM RFC were added, and the reaction was initiated by the addition of 20 nM Pol ε. Aliquots were removed at the indicated time points and stopped with 50 mM EDTA. Unincorporated nucleotide was removed with illusta MicroSpin G‐50 columns (GE Healthcare), and samples were run on 0.6% alkaline agarose gel at 23 V for 16 h. The gel was fixed with cold 5% trichloroacetic acid and dried onto Whatman paper. The gel was exposed on BAS‐IP MS Storage Phosphor Screen (GE Healthcare), and screen was developed on a Typhoon laser imager (GE Healthcare).

### Glycerol gradient preparation

Buffer A (40 mM HEPES‐NaOH, pH 7.5, 150 mM NaOAc, 0.5 mM TCEP, 500 μM AMP‐PNP, 3 mM Mg(OAc)_2_ and 10% v/v glycerol) was layered on top of an equal volume of Buffer B (Buffer A + 30% v/v glycerol) in a 2.2 ml TLS‐55 tube (Beranek Laborgerate) to prepare un‐crosslinked samples. For the generation of crosslinked samples, fresh Buffer B was supplemented with 0.16% glutaraldehyde (Sigma) and 2 mM bis(sulfosuccinimidyl)suberate (BS^3^, ThermoFisher). Gradients were prepared using a gradient‐making station (Biocomp Instruments, Ltd.) and cooled for 30 min at 4°C.

### Replisome assembly for cryo‐EM

The reconstitution reaction was set up to yield a final volume of 550 μl, containing 100 nM CMG with a 1.5‐fold molar excess of other components in reconstitution buffer (25 mM HEPES‐NaOH pH 7.6, 150 mM NaOAc, 0.5 mM TCEP, 500 µM AMP‐PNP, 10 mM Mg(OAc)_2_). Firstly, CMG was incubated with the fork DNA for 30 min on ice. Next, the additional proteins were added in the following order: AND‐1, TIMELESS/TIPIN, Pol ε and CLASPIN, and the volume adjusted to 550 μl. CLASPIN was omitted at this stage for the minus‐CLASPIN sample. The reaction was incubated for 30 min on ice prior to being loaded onto a gradient. 183 μl of the reconstitution reaction was loaded onto each gradient: one lacking crosslinker and two containing crosslinker. Samples were separated by centrifugation (Beckman TLS‐55 rotor, 200,000 *g*, 4°C, 2 h) and 100 μl fractions collected manually. Silver‐stained SDS–PAGE of samples +/− crosslinker was used to identify fractions containing the complete core replisome. The selected fractions from the crosslinked gradients were pooled and buffer exchanged into cryo‐EM buffer (reconstitution buffer lacking glycerol + 100 μM AMP‐PNP and 0.005% v/v Tween‐20 (Sigma, Cat#P8341)) through six rounds of concentration via centrifugation (21,000 *g*, 4°C, 1 min/round) and re‐dilution in a 0.5 ml 30K MWCO centrifugal filter (Amicon). Finally, the sample was concentrated to ˜30 μl and used for cryo‐EM grid preparation.

### Cryo‐EM grid preparation

Quantifoil R2/2, Cu‐400 mesh cryo‐EM grids pre‐coated with an ultra‐thin (3–5 nm) amorphous carbon (produced in‐house and by electron microscopy sciences) were glow discharged for 5 s at a plasma current of 15 mA (PELCO easiGlow). 3 μl of sample was applied and incubated for 45 s at 4°C before manually blotting with filter paper for 8 s and plunge‐freezing in liquid ethane.

### Data collection

#### Complete replisome

Three datasets were collected on the same FEI Titan Krios microscope (LMB Krios1), operating at 300 keV with the specimen at cryogenic temperatures (approximately −180°C), with images recorded at a defocus of between −1.5 and −3.5 μm. A total of 4,923 movies were acquired across two collections using the K2 Summit direct electron detector (Gatan) in electron counting mode with a GIF Quantum energy filter slit width of 20 eV, using a calibrated pixel size of 1.145 Å/pixel. These data were collected using the EPU software package (ThermoFisher) and the dose fractionated into 40 frames per movie, with an exposure time of 10 s to achieve a total dose of 39.8 e^‐^/Å^2^. An additional 2,400 movies were acquired using the Falcon III direct electron detector (ThermoFisher) in electron counting mode using a calibrated pixel size of 1.07 Å/pixel. 75 movie frames were recorded over 60 s to give a total dose of 37.5 e^−^/Å^2^.

#### Minus CLASPIN replisome

2998 movies were collected on a Titan Krios microscope (LMB Krios2), operated as described for the complete core replisome sample. Images were acquired at a defocus of between −1.5 and −3.5 μm using the K2 Summit direct electron detector (Gatan) in electron counting mode with a GIF Quantum energy filter slit width of 20 eV, using a calibrated pixel size of 1.1 Å/pixel. The total dose was fractionated into 40 frames, with an exposure time of 10 s to achieve a total dose of 39.2 e^−^/Å^2^.

### Data processing

#### Complete core replisome

Image processing was carried out using RELION 3.1 (Zivanov *et al*, 2018) unless otherwise stated. All refinements were performed using independent data half‐sets (gold standard refinement), and resolutions were determined based on the Fourier shell correlation (FSC = 0.143) criterion. The gain‐corrected movies were aligned using 5 × 5 patches in MotionCor2 (Zheng *et al*, 2017) with dose weighting. CTF estimation was carried out using CTFFIND‐4.1 (Rohou & Grigorieff, 2015). After manual inspection of the aligned micrographs for the complete replisome datasets, 331 micrographs were discarded due to the presence of crystalline ice. No micrographs were discarded from the minus CLASPIN dataset. Gautomatch (https://www2.mrc‐lmb.cam.ac.uk/research/locally‐developed‐software/zhang‐software/#gauto) was used to pick particles from the complete replisome micrographs. Initially, a subset of 500 micrographs were picked using 2D references generated from the previously published yeast replisome structure (Baretić *et al*, 2020). 20,214 particles were picked from this subset and submitted for two rounds of 2D classification. Five 2D classes were then selected from the results of this processing that contained high‐resolution features and represented diverse molecular views of the particle. These 2D classes were subsequently used as the templates to pick the entire complete replisome and minus CLASPIN datasets.

For the complete replisome data, a total of 490,110 particles were picked using Gautomatch. These particles were extracted and down‐sampled by a factor of four into a box of 100 pixels and submitted for one round of 2D classification. 360,349 particles were selected following 2D classification and submitted for 3D classification into four classes using a regularisation parameter of 4 and a 3D reference derived from the previously published structure of the yeast replisome. A single class was selected from the results of this 3D classification, comprising 280,190 particles, and the particles re‐extracted and down‐sampled by a factor of 2 into a box of 200 pixels. These particle images were then submitted for three further rounds of 3D classification, with classes being taken forward if they contained all replisome components and displayed structural features, e.g. helical density. Using these criteria, 138,400 particles were selected to be un‐binned into a box of 380 pixel diameter (435.5 Å) and submitted for 3D auto‐refinement. The results of the refinement were post‐processed, generating a reconstruction at a resolution of 3.8 Å. The data were then polished (Zivanov *et al*, 2019) and the CTF parameters refined, before being re‐submitted for 3D auto‐refinement and post‐processing, generating a reconstruction at 3.4 Å resolution. Using 3D classification without alignment, a subset of 110,266 particles was identified that displayed high‐resolution features. This subset was refined to 3.2 Å resolution and sharpened using a B‐factor of −35 Å^2^. This map was used to build atomic models for CMG, TIMELESS, TIPIN and DNA. Multi‐body refinement (Nakane *et al*, 2018) was performed by generating soft masks, generated in UCSF Chimera (Pettersen *et al*, 2004), around the MCM2‐7 N‐ and C‐tiers, TIMELESS‐TIPIN and DNA, AND‐1 and a complex of CDC45/GINS and Pol ε, the results of which were used to build the model of the Pol ε non‐cat module. An additional multi‐body refinement was carried out using masks covering a complex of CDC45/GINS and AND‐1, and the remainder of the map which significantly improved the density of AND‐1, permitting model building.

In order to recover density for the catalytic domain of Pol ε, the entire dataset was re‐picked using the Laplacian‐of‐Gaussian autopicking feature within Relion‐3.1. 560,443 particles that were auto‐picked were extracted and binned by a factor of four, into a box of 100 pixels. These particle images were classified using one round of 2D classification, resulting in 388,320 particles that were further classified in 3D. Following four rounds of 3D classification to remove low‐resolution classes and those lacking replisome components, 100,988 particles from two 3D classes were selected for refinement. The selected particle images were re‐extracted and binned by a factor of two, to boost the signal‐to‐noise of regions of weak density, into a box of 160 pixels. The data were submitted for 3D‐auto‐refinement which, following post‐processing, generated a reconstruction at 4.9 Å resolution. A soft mask was generated in UCSF Chimera covering the Pol ε non‐catalytic module, and signal subtraction was carried out to remove any signal outside of the mask boundary. The data were recentred on the mask and then submitted for 3D classification without alignment using a 3D reference of the complete core human replisome, recentred on the Pol ε non‐catalytic module. Of the ten classes generated, the best three, displaying secondary structure features were selected as representative of classes incorporating high‐quality Pol ε particles. The 87,877 signal‐subtracted particle images from the selected 3D classes were reverted to their original non‐signal‐subtracted parent images, re‐extracted into a larger box of 550 pixels (629.75 Å) and re‐submitted for 3D auto‐refinement and post‐processing, generating a reconstruction at 6.8 Å resolution. In order to identify density for the Pol ε catalytic domain, three soft masks were generated which represented putative regions of catalytic domain density: one covering the disordered density between AND‐1 and the Pol‐ε non‐cat module which appears during consensus refinement, a second mask representing the linear configuration of Pol ε identified in yeast (Yuan *et al*, 2020b) (EMD‐21707) and a third close to the MCM2‐7 C‐tier. These three masks were aligned on, and were merged with, the original mask covering the human Pol ε non‐catalytic module. Signal subtraction and 3D classification were then carried out as previously described in this section. Of the 15 classes generated, two contained additional density in the linear configuration. The 6,303 particles presented by these selected classes were reverted to their original.star file and refined and post‐processed to a resolution of 10 Å. The resulting map was used to dock in the structure of the complete yeast Pol ε holoenzyme.

#### cryoSPARC processing

Human replisome dataset #1, in the presence of CLASPIN, was additionally processed using cryoSPARC‐3 (Punjani *et al*, 2017). 3422 previously motion‐corrected micrographs were imported into the cryoSPARC pipeline and their CTF parameters estimated using the Patch CTF estimation, 5 × 5. The Blob‐Gaussian picking feature identified 602,412 particles. Following particle screening, 503,188 particle images were extracted and down‐sampled 4×. These images were classified in 2D and classes were selected that best resembled previous 2D classes obtained using Relion‐3.1. This resulted in 288,073 particles being submitted for two rounds of 3D classification via heterogeneous refinement, using four copies of an identical 3D ab initio model as a reference. Classes were selected based upon the presence of high‐resolution features and whether they contained the full complement of replisome proteins. The resulting 158,465 particles were refined to 3.3 Å resolution using homogenous refinement.

#### Minus CLASPIN replisome

For the minus‐CLASPIN replisome data, a total of 482,101 particles were picked by Gautomatch, using 2D references generated from the complete replisome dataset. These particles were extracted and down‐sampled by a factor of four into a box of 100 pixels and submitted for two rounds of 3D classification into four classes using a regularisation parameter of 4 and a 3D reference derived from the complete replisome data. Two classes were selected from the results of this 3D classification, comprising 107,833 particles based upon their protein composition and the presence of high‐resolution features. These selected particles were re‐extracted, un‐binned, into a box for 400 pixels (440 Å) and submitted for 3D auto‐refinement. The results of the refinement were post‐processed, generating a reconstruction at a resolution of 3.8 Å. The data were then polished and the CTF parameters refined, before re‐refinement and post‐processing which generated a reconstruction at a resolution of 3.4 Å.

### Model building and refinement

In order to begin building a model for the complete core human replisome, previously published atomic models for various replisome components were rigid‐body‐docked into the consensus refinement map at 3.2 Å resolution. Models for the MCM2‐7 N‐tier and both CDC45 and GINS came from the previous cryo‐EM structure of hsCMG (PDB: 6XTX) (Rzechorzek *et al*, 2020). As the C‐tier configuration of the structure of hsCMG differed to that of the human replisome structure presented here, the C‐tier region of each MCM2‐7 subunit (PDB: 6XTX) was docked individually into the density and both the linkers between the N‐ and C‐tier domains and the AMP‐PNP ligands removed. The crystal structure of the N‐terminal domain of TIMELESS (PDB: 5MQI) (Holzer *et al*, 2017) was docked into the map and this ensemble was used as the starting point for model building. First models were refined against the map density in real‐space using Phenix real‐space‐refine (Afonine *et al*, 2018) in the absence of secondary structure restraints. The models were then manually refined in Coot (Emsley *et al*, 2010) using the local refinement and regularisation tools incorporating stereochemical restraints. Where the density was of sufficient quality, we were able to expand the coverage of the starting models by building into the density de novo using Coot.

For TIMELESS, we were able to build the previously absent MCM‐plugin (residues 239–332) and extend the C‐terminal region of the protein (residues 464–803) containing both DNA‐binding motifs and the TIPIN interaction domain. TIMELESS residues 527–684 were not visualised in this study. A homology model was generated for TIPIN (residues 68–132) using I‐TASSER (Yang *et al*, 2015), based on the structure of Csm3 and rigid body‐docked into the density. The N‐ and C‐terminal regions were expanded to cover residues 62–147 manually in Coot.

For the MCM2‐7 subunits, the MCM6 N‐terminus is extended (residues 1–14) and interacts extensively with the core of TIMELESS. An additional 11 residues of the N‐terminal extension of MCM4 is visualised (residues 146–157) interacting with the wedge feature of the TIMELESS MCM‐plugin. The first 14 residues of MCM3 in the published human hsCMG model (PDB: 6XTX) (Rzechorzek *et al*, 2020) are re‐assigned to MCM3 residues 524–533. In MCM7, an additional, flexibly linked helix (residues 100–114) is identified. The N‐terminal hairpin of MCM7 (residues 283–290) was built as a short helix. The N/C‐tier flexible linkers were re‐built for each subunit. The MCM2‐7 C‐tier was re‐built to accommodate an alternative DNA‐binding mode, with the PS1 loops, helix H2 and H2I loops extensively remodelled. A homology model was generated for the winged‐helix (WH) of MCM4 (residues 798–857) which was rigid body docked into the lower resolution density sat within the C‐tier pore and subjected to real‐space‐refinement in Phenix. The placement of this domain was guided by the reasonable resolution density for the first helix of the MCM4 WH domain. AMP‐PNP/Mg^2+^ was built in well resolved density at the MCM2/6, 2/5 and 3/5 interfaces with side chains visible for WalkerA, WalkerB, Arg‐finger and Sensor2 motifs. Eleven nucleotides of ssDNA within the C‐tier were built de novo whereas the duplex portion of the DNA was rigid body docked as an idealised B‐form duplex. Sequence register was assigned based on the sequence of our fork DNA assuming no unwinding occurred. DNA at the fork junction was refined manually in Coot and the first nucleotides following strand separation built manually.

The crystal structure of the AND‐1 SepB domain (PDB: 5OGS) (Kilkenny *et al*, 2017) was docked into the AND‐1 trimer density within the multi‐body refinement map containing CDC45/GINS and AND‐1. The position of residues at the interface between AND‐1 and CDC45/GINS was adjusted manually in Coot and the fit to density optimised using Phenix real‐space‐refine.

A homology model for the non‐catalytic module of Pol ε (POLE1 residues 1,371–2,280 and POLE2 residues 1–527) was generated using I‐TASSER and rigid body docked into the multi‐body refinement map containing CDC45/GINS and Pol ε. Two of the top threading templates were a crystal structure of human POLE2 in complex with a C‐terminal region of POLE1 (PDB: 5VBN) (Baranovskiy *et al*, 2017) and an NMR structure of the N‐terminal helical domain of POLE2 (PDB: 2V6Z) (Nuutinen *et al*, 2008). Despite the existence of the NMR structure covering this region, residues 1–85 of POLE2, comprising the N‐terminal helical domain and flexible linker, were manually built manually using Coot due to the high quality of the data. The fit‐to‐density of the resulting homology model was optimised using ISOLDE and residues at the interfaces with CMG were manually optimised in Coot followed by real‐space‐refinement in Phenix. The resulting model displayed high levels of structural homology with previously published structures: RMSD of 1.21 Å for 5VBN (Baranovskiy *et al*, 2017) and 1.23 Å for 2V6Z (Nuutinen *et al*, 2008).

AlphaFold (Jumper *et al*, 2021; Tunyasuvunakool *et al*, 2021) was used to identify candidate regions of CLASPIN to dock into CLASPIN‐dependent densities 1–3. CLASPIN‐dependent density 1 consists of two α‐helical segments of density, connected by a linker. The shorter of the two α‐helices comprises approximately 5 amino acids and contains density for three large, bulky residues. The longer of the two helices comprises approximately 15 residues. To estimate the length of the linker region between the two helices, a 13‐residue poly‐alanine model was manually built into the density. Manual inspection of the AlphaFold predicted model for *H. sapiens* CLASPIN identified only one region of sequence, residues 277–318, which satisfied these structural requirements. Rigid‐body docking of the AlphaFold model for CLASPIN residues 277–318 into CLASPIN‐dependent density 1 resulted in an excellent fit‐to‐density, with clear side‐chain density correctly positioned for CLASPIN H315, F317, F318. The model fit‐to‐density was improved manually using COOT and automatically using both ISOLDE and PHENIX real‐space‐refinement. The resulting model displayed clear side‐chain density for residues in the linker region between the two helices, particularly P305, Y306, H307 and P309. It also correctly oriented the larger of the two α‐helices, residues 284–299, displaying clear side‐chain density for R298 which interacts with Y474 of TIMELESS. In addition to the excellent fit‐to‐density, the interactions predicted by the model for CLASPIN make energetically favourable and chemically feasible interactions. CLASPIN residues F317 and F318 extend into a conserved hydrophobic pocket in TIMELESS, L304 and L302 contact another hydrophobic patch on TIMELESS and polar residues in the longer of the two α‐helices form charged interactions at a third site on TIMELESS. Finally, the equivalent region of Mrc1, the *S. cerevisiae* ortholog of CLASPIN, can be docked into previously unmodelled density present in the analogous position to site 1 in a yeast replisome reconstruction (EMD‐10227) (Baretić *et al*, 2020), with clear side‐chain density present for Mrc1 residues F325, F326, F331 and F335.

CLASPIN‐dependent density 2 consists of a single α‐helix approximately 12–15 residues in length. This helical segment docks onto a highly hydrophobic pocket on the C‐tier of MCM6. Inspection of the density indicates the presence of three large bulky residues within the helix. There is only one candidate helix present in the AlphaFold predicted model that satisfies these requirements, spanning residues 526–539. Rigid‐body docking of this helix into CLASPIN‐dependent density 2 following by fit optimisation using COOT, ISOLDE and PHENIX resulted in an excellent fit‐to‐density. There is clear side‐chain density for residues H538, W536, F535, K532, R534 and L531. Furthermore, this helix positions F535 and L531 into the hydrophobic pocket on MCM6 while K532 and R534 project away from the replisome, satisfying the chemical requirements of the interface.

CLASPIN‐dependent density 3 consists of a single α‐helix approximately 26‐residues in length. The AlphaFold predicted model for *H. sapiens* CLASPIN reveals only three candidate helices of sufficient length the occupy this density. Each of the three candidate helices was rigid‐body‐docked into the density and the fit optimised using COOT, ISOLDE and PHENIX. The fit to density for the helix comprising CLASPIN residues 592–625 was excellent and far superior to the other two candidate helices: residues 1,091–1,121 and residues 1,205–1,221. There is clear side‐chain density for residues K593, Q595, V596, K598, K600, Q602 and M605. There is also clear density for CLASPIN residues L594, L597 and L601 which contact a hydrophobic patch on MCM2. The model for CLASPIN site 3 also predicts many residues forming chemically favourable interactions with both TIMELESS, MCM2 and MCM6. Furthermore, the other two candidate helices are in the C‐terminal region of CLASPIN, which is not predicted to interact with this region of the replisome based upon cross‐linking mass spectrometry data of the budding yeast replisome (Baretić *et al*, 2020). Finally, the relative positioning of the helices occupying CLASPIN sites 2 and 3 is in agreement with the AlphaFold model, with there being no intervening helices between them.

### Minus CLASPIN replisome

For the minus CLASPIN replisome, the model for the complete replisome was rigid body docked into the density (minus the candidate CLASPIN poly‐alanine chain) and the fit optimised using Phenix real‐space‐refine and manual editing in Coot.

### Combine focussed maps

The complete replisome model, consensus refinement map at 3.2 Å and the two multi‐body refinement maps used to build Pol ε and AND‐1 were submitted to the combine focussed maps feature of the Phenix software package. Combine‐focussed‐maps uses map‐to‐model correlation to determine which are the highest resolution regions of each map, and the relationships between the models in the different maps. This information is then used to superimpose the highest resolution regions of each map to generate a single composite map. This permitted the refinement of the complete replisome model within a single map using real‐space‐refinement in Phenix and ISOLDE, coupled to manual optimisation in Coot.

### Model to cryo‐EM map validation

Fourier shell correlation (FSC) between the fully refined models +/− CLASPIN and the respective unsharpened sums of their two half maps was calculated using XMIPP (Sorzano *et al*, 2004).

### Multiple sequence alignments

Amino acid sequences were retrieved from UniProt and protein sequence alignments carried out using Clustal Omega (Sievers & Higgins, 2014). Alignments were rendered using ESPript3.0 (http://espript.ibcp.fr) (Robert & Gouet, 2014).

### Structural analysis and visualisation

All figures of structures were generated in either Chimera or ChimeraX. Calculations of buried surface area were performed using PDBePISA (Krissinel & Henrick, 2007).

## Author contributions

MLJ prepared samples, performed all cryo‐EM data collection and processing and built the atomic models. YB generated expression constructs, purified proteins, performed helicase and polymerase assays and preliminary analysis of replisome complex formation. MRGT generated expression constructs and purified proteins. JTPY and MLJ analysed data and wrote the manuscript.

## Conflict of interest

The authors declare that they have no conflict of interest.

## Supporting information



Appendix

Expanded View Figures PDF

## Data Availability

Cryo‐EM density maps of the human replisome used in model building have been deposited in the Electron Microscopy Data Bank (EMDB), https://www.ebi.ac.uk/pdbe/emdb, under the following accession numbers: EMD‐13375 (full complex, consensus refinement), EMD‐13377 (multi‐body refinement, Pol ε/CDC45/GINS), EMD‐13376 (multi‐body refinement, AND‐1/CDC45/GINS), EMD‐13384 (minus CLASPIN, consensus refinement). Atomic coordinates have been deposited in the Protein Data Bank (PDB), http://www.pdb.org, with the accession number PDB: 7PFO for the complete core human replisome. The cryo‐EM map with density that we attribute to the lagging‐strand template has been deposited in the EMDB with accession number EMD‐13457.
