# Supplementary material for: Structure of a human replisome shows the organisation and interactions of a DNA replication machine
Source: EMBO J. 2021 Oct 25;40(23):e108819. doi: 10.15252/embj.2021108819 (PMC8634136; doi:10.15252/embj.2021108819)
Supplement: Supplementary file 1 — Appendix [file EMBJ-40-e108819-s002.pdf]

Table of Contents

Appendix Figure S1	Page 2
Appendix Figure S2	Page 3
Appendix Figure S3	Page 4
Appendix Table S1	Page 5
Appendix Table S2	Page 6
Appendix Table S3	Page 7

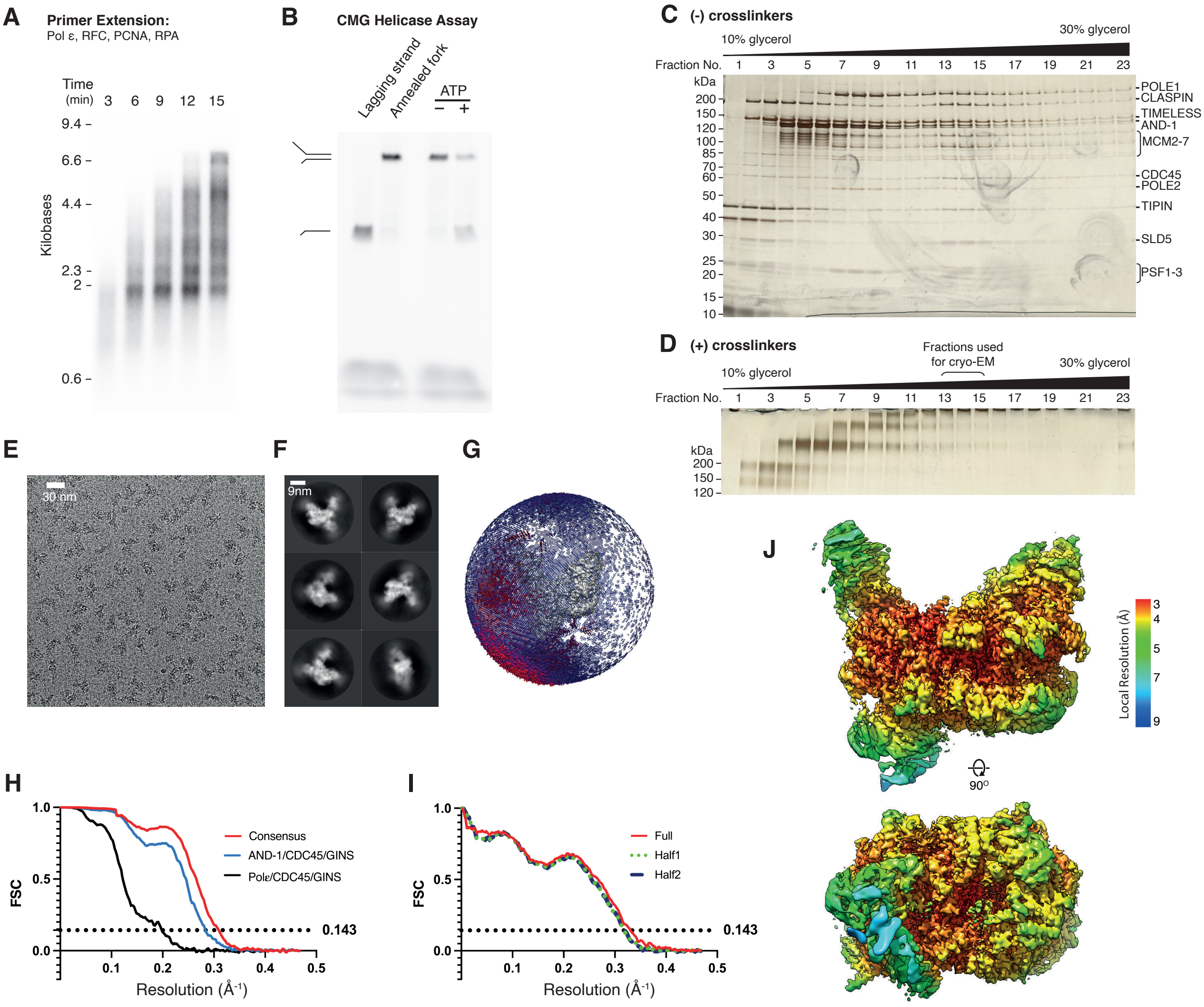

Appendix Figure S1. Assembly of the core human replisome and analysis by cryo-EM.

A Primer extension assay illustrating template extension by Pol ε from 3 to 15 minutes in a reaction including Pol ε, PCNA, RFC, RPA and CMG.

B CMG helicase assay illustrating the unwinding of fork DNA by CMG in an ATP dependent manner.

C Silver-stained SDS-PAGE analysis of a core human replisome assembly separated through a native glycerol gradient.

D As in (**C**) but with the inclusion of the crosslinkers glutaraldehyde and BS^3 in the glycerol gradient.

E Representative cryo-EM micrograph following motion (Zheng *et al.*, 2017) and gain-correction, scale bar indicates 30 nm.

F Representative cryo-EM 2D class averages, mask diameter 340 Å.

G 3D angular distribution of particle projections.

H Representative Fourier shell correlation (FSC) curves for consensus refinement and multi-body maps used in model building (Liebschner *et al*, 2019). The FSC=0.143 criterion used to determine map resolution is indicated as a dotted black line.

I Map-to-model FSC curves, generated using XMIPP, for the complete refined model relative to the full (half map sum) and individual half maps for the consensus refinement.

J Two views of a composite map, generated using Phenix Combine-Focused-Maps, incorporating the highest resolution regions of the three maps described in panel F. The map is coloured by local resolution according to the inset key.

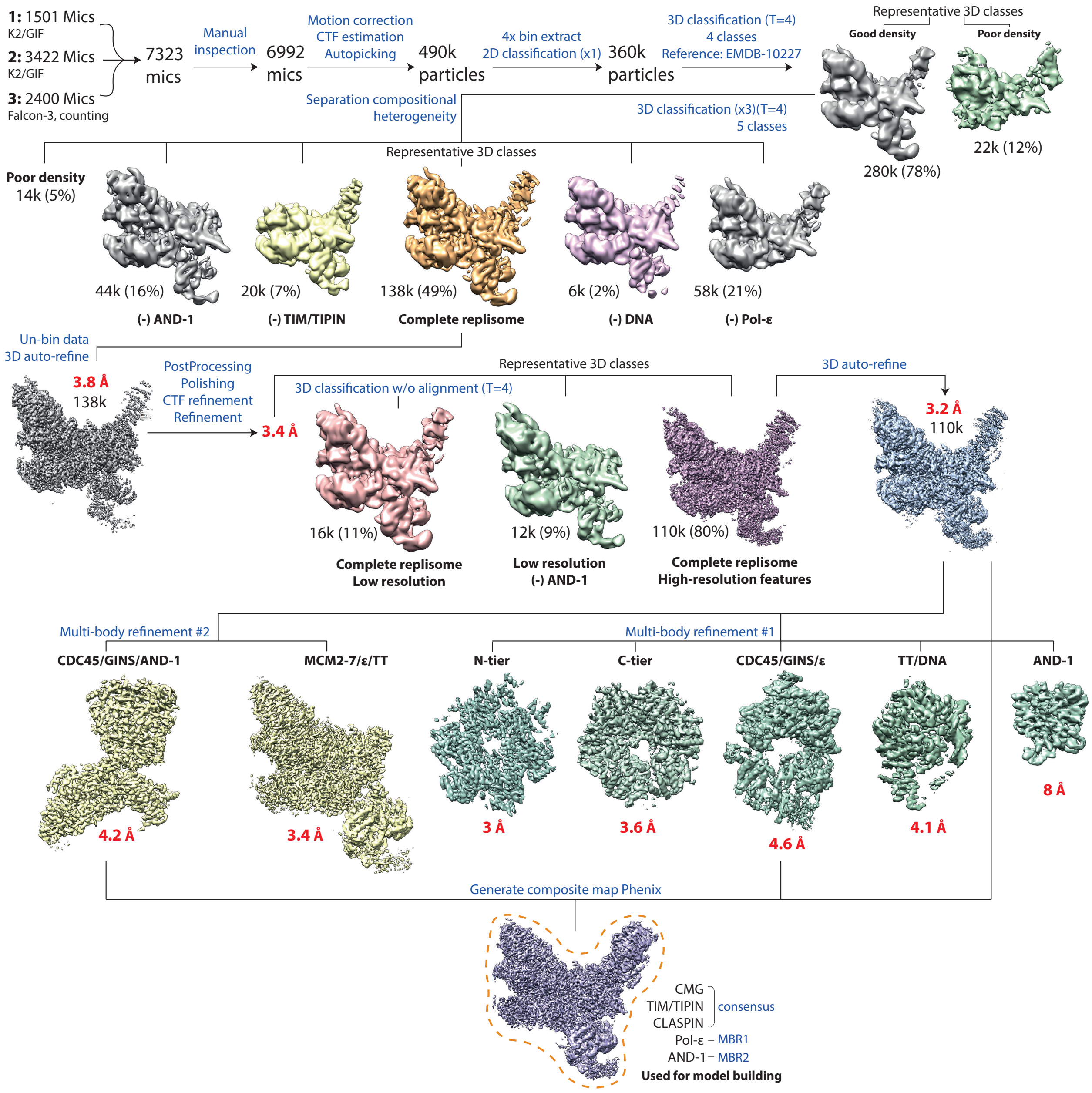

Appendix Figure S2. Data processing pipeline for the core human replisome.
Schematic documenting the various steps in the processing-pipeline for the core human replisome sample. Results of individual multi-body refinement jobs are colour coded. The consensus refinement at 3.2 Å resolution, CDC45/GINS/AND-1 multi-body refinement at 4.2 Å resolution and the CDC45/GINS/Pol ε multibody refinement at 4.6 Å resolution were used for model building.

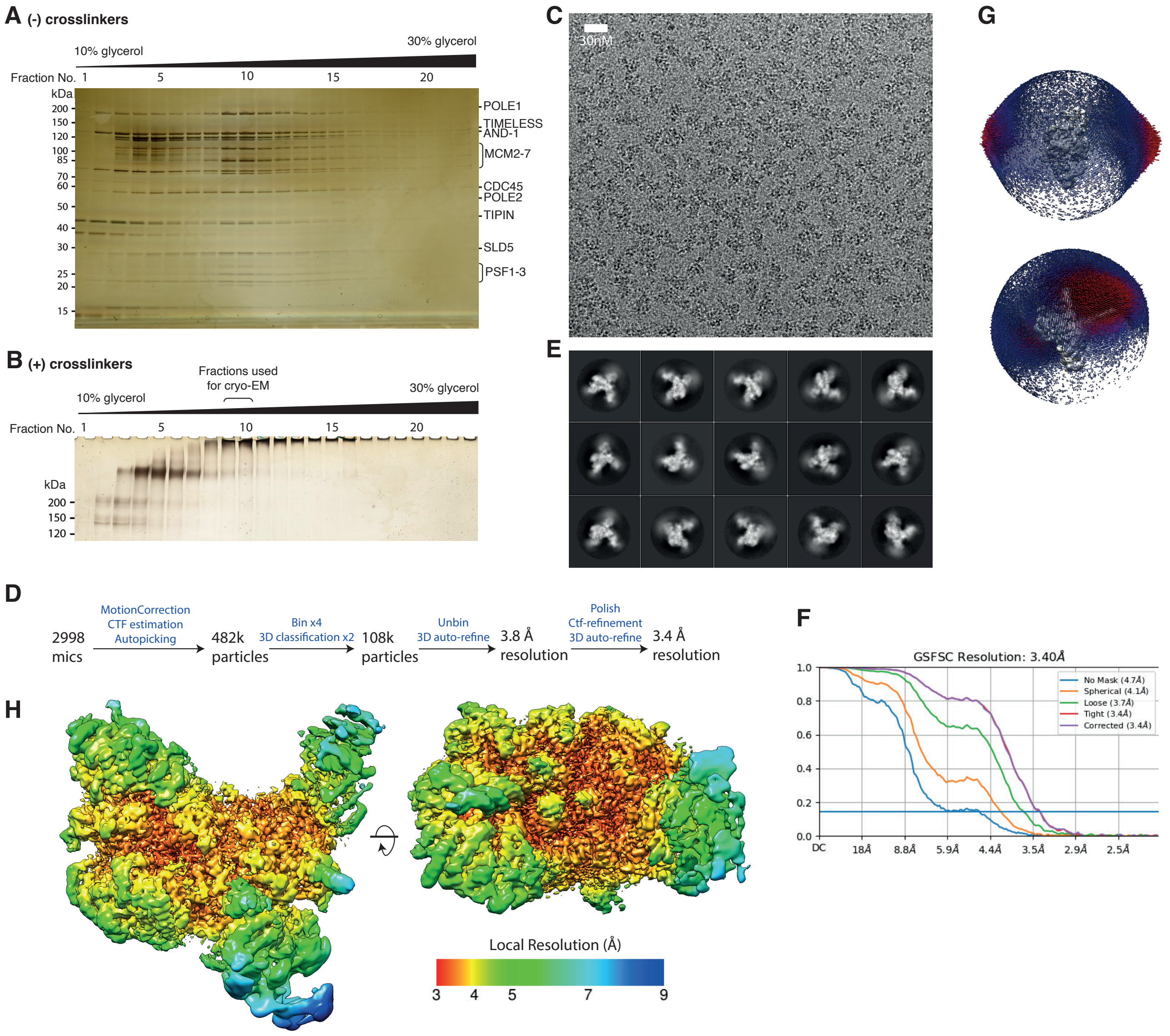

Appendix Figure S3. Structure of the core human replisome lacking CLASPIN.

A Silver stained SDS-PAGE analysis of a core human replisome assembly lacking CLASPIN separated through a native glycerol gradient.

B As in (**A**) but with the inclusion of the crosslinkers glutaraldehyde and BS3 in the glycerol gradient.

C Representative cryo-EM micrograph following motion (Zheng *et al.*, 2017) and gain-correction, scale bar indicates 30 nm.

D Schematic processing pipeline used to generate a 3.4 Å resolution replisome reconstruction in the absence of CLASPIN.

E Representative cryo-EM 2D class averages, mask diameter 350 Å.

F Fourier shell correlation (FSC) curve for the replisome refinement in the absence of CLASPIN to 3.4 Å resolution. The FSC=0.143 criterion was used to determine map resolution.

G 3D angular distribution of particle projections.

H Two views of refined replisome map in the absence of CLASPIN, at 3.4 Å resolution, coloured by local resolution according to the inset key.

Appendix Table S1. UniProt identification numbers for protein sequences used for codon optimisation, related to experimental procedures.

Gene	Uniprot identifier
MCM2	P49736
MCM3	P25205-1
MCM4	P33991
MCM5	P33992
MCM6	Q14566
MCM7	P33993-1
CDC45	O75419-1
PSF1	Q14691
PSF2	Q9Y248
PSF3	Q9BRX5
SLD5	Q9BRT9-1
POLE1	Q07864
POLE2	P56282
POLE3	Q9NRF9
POLE4	Q9NR33
CLASPIN	Q9HAW4-1
AND-1	O75717-1
TIPIN	Q9BVW5
TIMELESS	Q9UNS1-1

Appendix Table S2. Affinity tags for protein purifications, related to experimental procedures.

Protein	Affinity tag	Tag sequence
**CMG**	N-terminal Twin-Strep HRV 3C on SLD5	WSHPQFEKGGGSGGGSGGSAWSHPQFEKSGLEVLFQGP
	Internal FLAG in CDC45<br>L160 - DYK - E161D, E162D, E163D<br>(residue numbers correspond to the original CDC45 sequence)	DYKDDD
**AND-1**	N- terminal 3X FLAG TEV	DYKDDDDKDYKDDDDKDYKDDDDKENLYFQG
**CLASPIN**	C-terminal HRV 3C 3X FLAG	LEVLFQGPDYKDDDDKDYKDDDDKDYKDDDDK
**Pol ε**	C-terminal TEV CBP on POLE3	ENLYFQGEKRRWKKNFIAVSAANRFKKISSSGAL
**TIMELESS-TIPIN**	N-terminal Twin-Strep TEV on TIMELESS	WSHPQFEKGGGSGGGSGGSAWSHPQFEKSGENLYFQG

Appendix Table S3. Plasmids used for protein expression, related to experimental procedures.

Plasmid name	Protein	Cloning details
MT_H1	MCM2	Synthetic construct cloned BamHI / XbaI into pACEBac1
MT_W1	MCM3	Synthetic construct cloned BamHI / XbaI into pACEBac1
MT_Q1	MCM4	Synthetic construct cloned BamHI / XbaI into pACEBac1
MT_JA1	MCM5	Synthetic construct cloned BamHI / XbaI into pACEBac1
MT_P1	MCM6	Synthetic construct cloned BamHI / XbaI into pACEBac1
MT_K1	MCM7	Synthetic construct cloned BamHI / XbaI into pACEBac1
MT_O1	1X FLAG- CDC45	Synthetic construct cloned BamHI / XbaI into pACEBac1
MT_D1	PSF1	Synthetic construct cloned BamHI / XbaI into pACEBac1
MT_E1	PSF2	Synthetic construct cloned BamHI / XbaI into pACEBac1
MT_F1	PSF3	Synthetic construct cloned BamHI / XbaI into pACEBac1
MT_G1	TWIN STREP- SLD5	Synthetic construct cloned BamHI / XbaI into pACEBac1
YB_1	GINS	pBIG1a vector for expression of GINS
YB_2	MCM2-7	pBIG2ab vector for expression of MCM2-7
MT_U2	POLE1	Synthetic construct cloned BamHI / XbaI into pACEBac1
MT_L1	POLE2	Synthetic construct cloned BamHI / XbaI into pACEBac1
MT_M1	POLE3	Synthetic construct cloned BamHI / XbaI into pACEBac1
MT_N1	POLE4	Synthetic construct cloned BamHI / XbaI into pACEBac1
MT_X15	POLE1-E4	pBIG1a vector for expression of Polε
MT_BF1	AND1	Synthetic construct cloned BamHI / XbaI into pACEBac1
MT_DF1	TIMELESS	Synthetic construct cloned BamHI / XbaI into pACEBac1
MT_BD1	TIPIN	Synthetic construct cloned BamHI / XbaI into pACEBac1
MT_BH1	TWIN STREP-RFC1	Synthetic construct cloned BamHI / XbaI into pACEBac1
MT_BI1	RFC2	Synthetic construct cloned BamHI / XbaI into pACEBac1
MT_BJ1	RFC3	Synthetic construct cloned BamHI / XbaI into pACEBac1
MT_BK1	RFC4	Synthetic construct cloned BamHI / XbaI into pACEBac1
MT_BL1	RFC5	Synthetic construct cloned BamHI / XbaI into pACEBac1
MT_EB1	PCNA	Synthetic construct cloned NcoI / BamHI into pET28a
