# Supplementary material for: Structure of a human replisome shows the organisation and interactions of a DNA replication machine
Source: EMBO J. 2021 Oct 25;40(23):e108819. doi: 10.15252/embj.2021108819 (PMC8634136; doi:10.15252/embj.2021108819)
Supplement: Supplementary file 2 — Expanded View Figures PDF [file EMBJ-40-e108819-s001.pdf]

Expanded View Figures

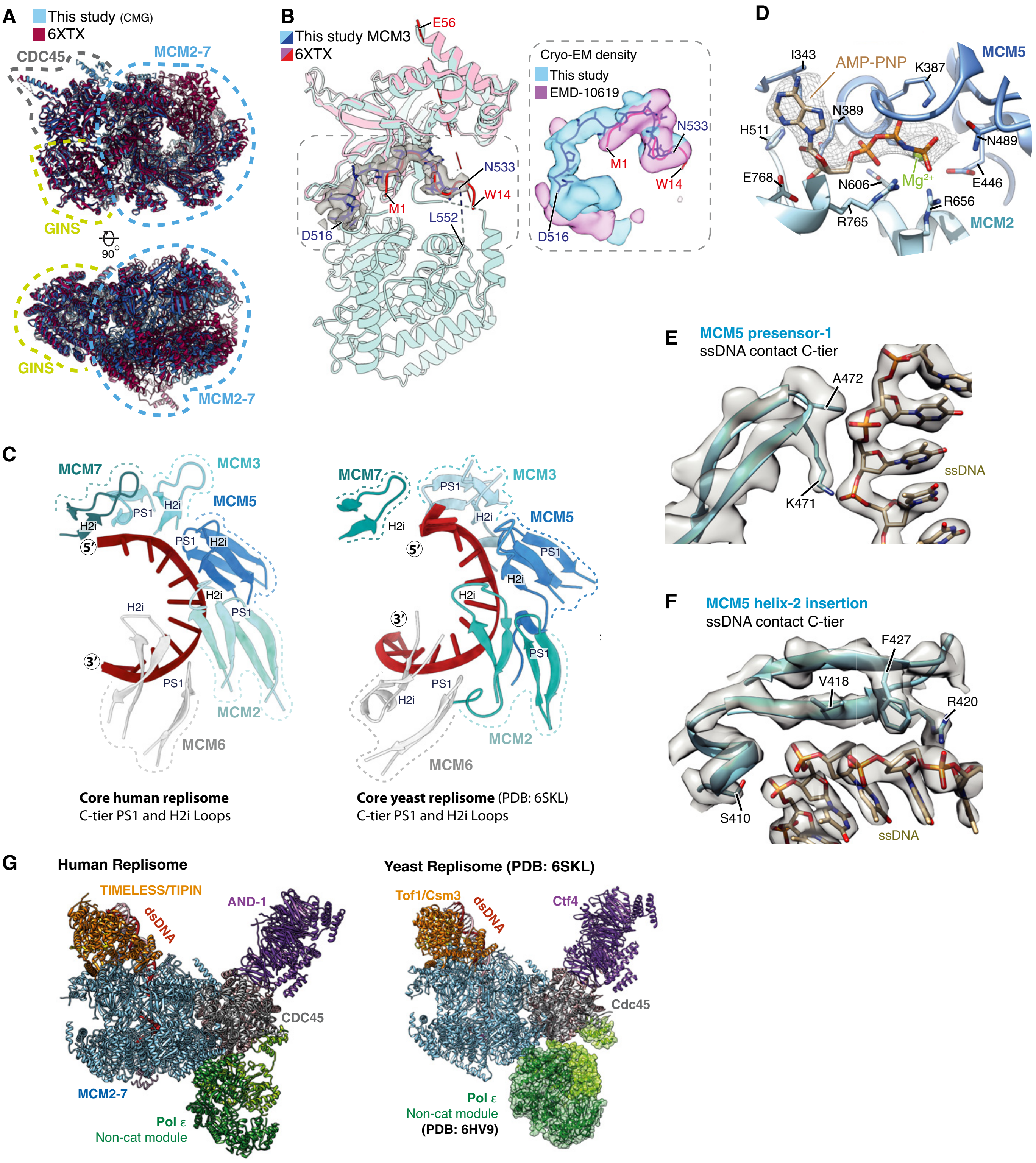

Figure EV1.

© 2021 MRC Laboratory of Molecular Biology.

Figure EV1. (Related to Fig 1). MCM2-7 engagement with ssDNA in the C-tier.

A Comparison of the MCM N-tier, GINS and CDC45 between the core replisome structure from this study (blue) (PDB: 7PFO) and the hsCMG:ssDNA structure (red) (PDB: 6XTX) (Rzechorzek *et al*, 2020).

B (Left) Comparison of the MCM3 model from this study (blue) with the N-tier lobe of MCM3 from the hsCMG:ssDNA structure (pink). Cryo-EM density for residues D516-N533 from this study is shown as a transparent grey surface. This region was assigned to the N-terminal extension of a longer MCM3 isoform in the hsCMG:ssDNA structure (Rzechorzek *et al*, 2020). (Right) Overlay of cryo-EM density from this study (blue) and the previous hsCMG:ssDNA map (pink). Despite the isoform used in this study not containing this N-terminal extension, almost identical density is observed, which in our map shows clear connectivity with the MCM3 C-terminal domain. We therefore attribute this density to MCM3 residues 524–533.

C Comparison of ssDNA engagement by the PS1 and H2i loops between the core human (left) and *S. cerevisiae* replisomes (right) (PDB: 6SKL) (Baretić *et al*, 2020).

D Detailed view of the MCM2:5 ATPase site. Cryo-EM density for AMP-PNP is shown as mesh.

E, F Models illustrating MCM5 engagement with ssDNA in the C-tier. Cryo-EM density represented as transparent grey surface with selected side chains contacting ssDNA displayed. (E) Representative density for the PS1 loop (MCM5) interacting with the phosphodiester ssDNA backbone. (F) Representative density for the H2i loop (MCM5) interacting with ssDNA in the C-tier.

G Comparison of the core human (left) and *S. cerevisiae* (right) (PDB: 6SKL) replisomes. Models are coloured as in Fig 1C. For the *S. cerevisiae* replisome, the Pol ϵ non-cat module has been positioned based on the structure of scCMG:Pol ϵ (PDB: 6HV9) (Goswami *et al*, 2018).

© 2021 MRC Laboratory of Molecular Biology.

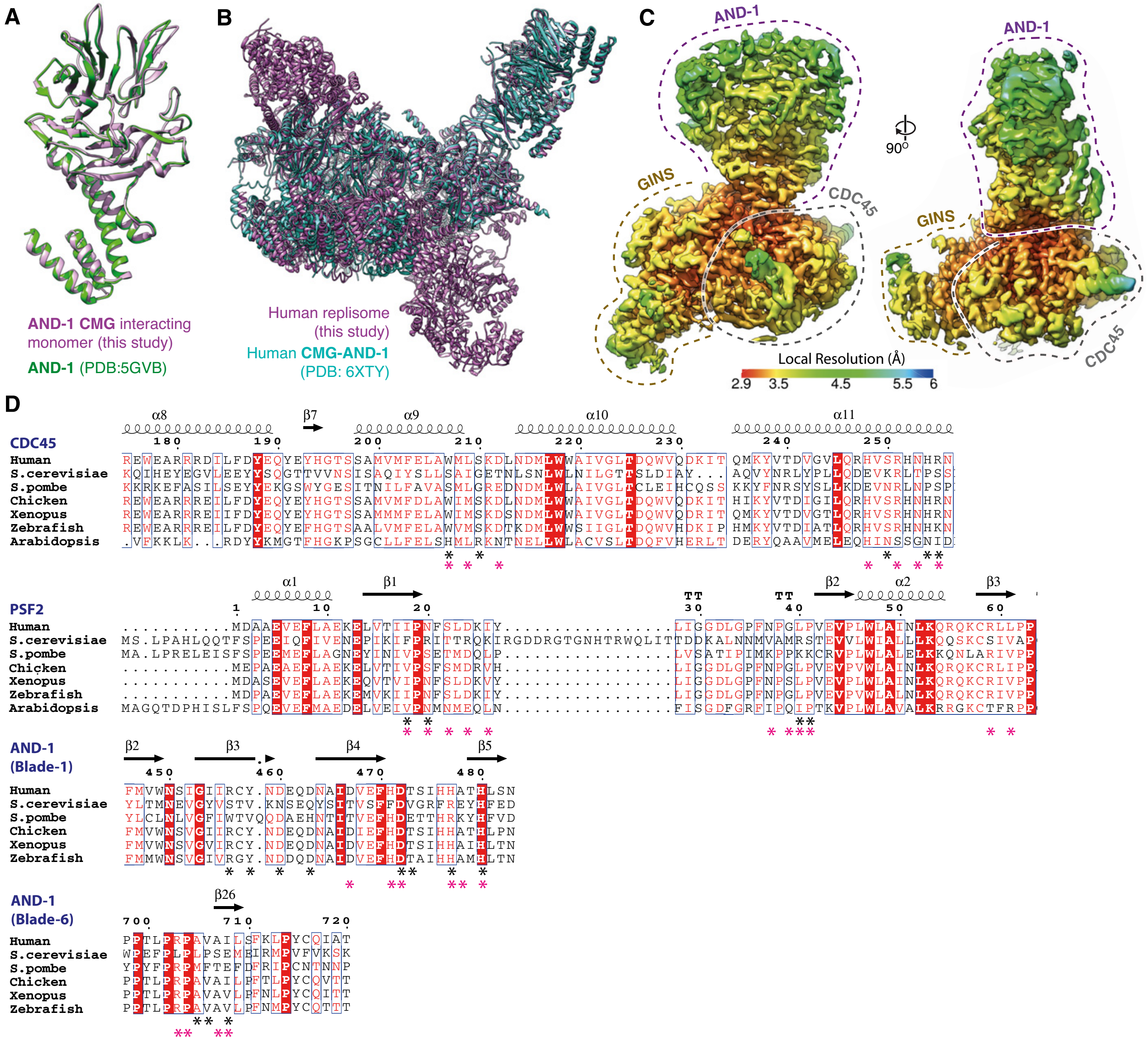

Figure EV2. (Related to Fig 2). Structure of AND-1 in the replisome.

A Comparison of the AND-1 SepB domain from the core replisome (this study, purple) with the crystal structure of the isolated protein (PDB: 5GVB, green) (Guan *et al*, 2017).

B Comparison of the positioning of AND-1 in the core replisome (this study, purple) with the CMG:AND-1 structure (PDB: 6XTY, cyan) (Rzechorzek *et al*, 2020).

C Multi-body cryo-EM map comprising AND-1, CDC45 and GINS coloured by local resolution according to inset key.

D Multiple sequence alignments of residues involved in the AND-1/CMG interface. Residues making specific AND-1/CMG or Ctf4/CMG interface contacts are marked by a black (human) or pink (*S. cerevisiae*) asterisk. Alignments carried out using NCBI Clustal Omega (Sievers & Higgins, 2014) and visualised using ESPrit (Robert & Gouet, 2014) with the primary human sequence indicated. Uniprot ID for sequences used for CDC45 alignment: *H. sapiens* (O75419-1), *S. cerevisiae* (Q08032-1), *S. pombe* (O74113-1), *G. gallus* (E1BYS7-1), *X. laevis* (Q9YHZ6-1), *D. rerio* (Q7ZU79-1), *A. thaliana* (Q9LSG6-1). PSF2 alignment: *H. sapiens* (Q03519-1), *S. cerevisiae* (P40359-1), *S. pombe* (O94329-1), *G. gallus* (A0A1D5PQM4-1), *X. laevis* (Q7ZT46-1), *D. rerio* (Q4VBJ6-1), *A. thaliana* (Q9C7A8-1). AND-1 alignment: *H. sapiens* (O75717-1), *S. cerevisiae* (Q01454-1), *S. pombe* (Q9C107-1), *G. gallus* (P30985-1), *X. laevis* (O13046-1), *D. rerio* (A0A0R4J7S6-1), *A. thaliana* (Q9ZQX6-1).

© 2021 MRC Laboratory of Molecular Biology.

Figure EV3. (Related to Fig 3). Structure of TIMELESS-TIPIN.

A Cryo-EM density coloured according to protein chain occupancy, except for the TIMELESS “MCM-plugin” element which is highlighted in red. For clarity DNA density is not shown.

B Assignment of the TIMELESS helical repeats. Model is coloured according to helical repeat and the α helices numbered.

C Structural comparison between TIMELESS from this study (brown) and yeast Tof1 (blue) (PDB: 6SKL) (Baretić *et al*, 2020).

D Multiple sequence alignment region covering the Tof-1 Ω-loop. All alignments in the figure were carried out using NCBI Clustal Omega and visualised using ESPrit with the primary human sequence indicated. Uniprot ID for sequences used for TIMELESS alignments: *H. sapiens* (Q9UNS1-1), *S. cerevisiae* (P53840-1), *S. pombe* (Q9UUM2-1), *G. gallus* (Q8QGQ6-1), *X. laevis* (A0A6I8PXH0-1), *D. rerio* (E7FGL0-1), *A. thaliana* (A0A1P8B9S9-1).

E–J, L Detailed views of the interactions between the TIMELESS MCM-Plugin and specific MCM subunits as indicated.

G (Bottom) multiple sequence alignment covering residues that form the TIMELESS Anchor motif, alignment carried out as described in EV3D.

K Sequence alignment of Wedge from *H. sapiens* TIMELESS and *S. cerevisiae* Tof1 (PDB: 6SKL) (Baretić *et al*, 2020), alignment carried out as described in EV3D.

M The position of the MCM6 N-terminus (purple), which occupies a cavity formed by helical repeats 3, 4 and 5 of TIMELESS and the MCM-plugin. Models shown as cartoons with the MCM6 N-terminus also displayed using transparent surface rendering.

N The cavity occupied by the MCM6 N-terminus in the core human replisome is partially blocked by the Tof-1 Ω-loop and MCM-plugin in *S. cerevisiae* (PDB: 6SKL) (Baretić *et al*, 2020). Model of TIMELESS, displayed as a cartoon, overlaid with the Tof-1 Ω-loop (gold) and Tof-1 MCM-plugin (grey) displayed as a cartoon with transparent surface rendering. The approximate position adopted by the MCM6 N-terminus in the core human replisome is indicated with a dashed purple line.

O, P Detailed views of the interactions between the TIMELESS α-solenoid and MCM2 N-terminal helical domain (O) and MCM6 zinc finger (P). TIMELESS model displayed using cartoon rendering with the surface of MCM2 and MCM6 displayed using transparent surface rendering.

© 2021 MRC Laboratory of Molecular Biology.

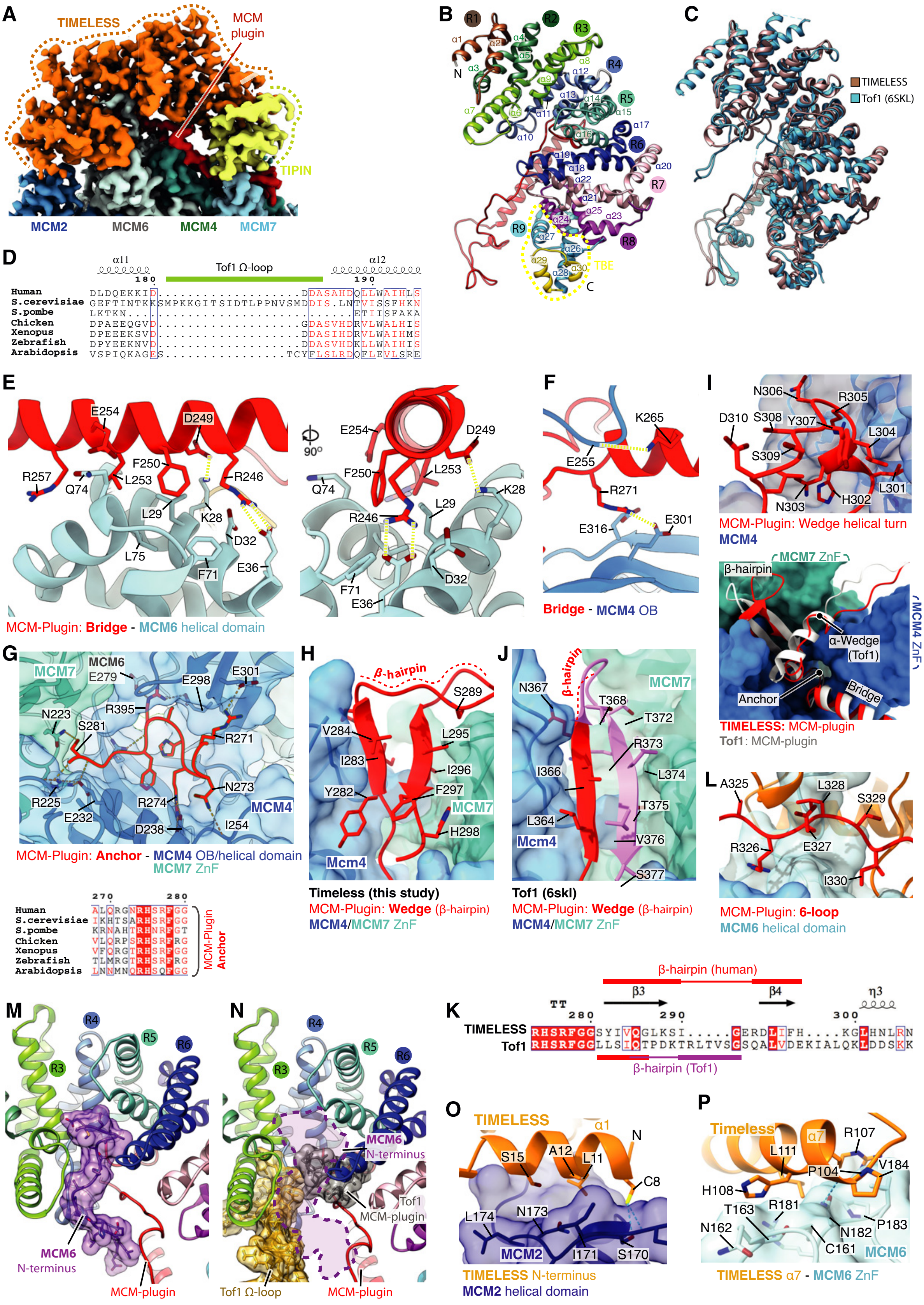

Figure EV3.

© 2021 MRC Laboratory of Molecular Biology.

Figure EV4. (Related to Fig 4). Structure of Pol ϵ.

A Cryo-EM map obtained using MultiBody refinement (Nakane *et al*, 2018) masking over the Pol ϵ non-catalytic module, CDC45 and GINS. Map coloured by local resolution according to inset key.

B Model for the Pol $\epsilon^{\text{non-cat}}$ domain as part of the core human replisome (other replisome components not shown). Models displayed as cartoons overlayed with transparent surface rendering. (Top) POLE1 is coloured green and POLE2 in blue. POLE2 domains are annotated and their position indicated using dashed lines. (Bottom) Model for POLE1 displaying the "wide-open jaw" configuration. Approximate positions of the inactive polymerase and exonuclease motifs are indicated and the C-terminal ZnF coloured red.

C Comparison between the structure of the Pol $\epsilon^{\text{non-cat}}$ domain from this study (grey) with previous structures of POLE2 (orange) with a C-terminal region of POLE1 (Baranovskiy *et al*, 2017) (yellow) (PDB: 5VBN) and the N-terminal helical domain of POLE2 (red) (PDB: 2V6Z) (Nuutinen *et al*, 2008).

D (Top) Detailed view of the interface between POLE1 (green) and the MCM2 winged-helix domain (blue). Atomic models visualised using cartoon rendering with selected side chains displayed and corresponding cryo-EM density is overlaid in transparent mesh. (Bottom) Multiple sequence alignment for residues involved in the MCM2-WH/POLE1 interface. Orange bars indicate the pattern of charge conserved residues along the same face of POLE1 α22, residues that make inter-protein contacts are marked with an asterisk. All alignments in the figure were carried out using NCBI Clustal Omega and visualised using ESPrit with the primary human sequence indicated. Uniprot ID for sequences used for POLE1 alignment: *H. sapiens* (Q07864-1), *S. cerevisiae* (P21951-1), *S. pombe* (P87154-1), *G. gallus* (E1C5P2-1), *X. laevis* (A0A1L8HZP3-1), *D. rerio* (B0V351-1), *A. thaliana* (F4HW04-1). MCM2 alignment: *H. sapiens* (P49736-1), *S. cerevisiae* (P29469-1), *S. pombe* (P40377-1), *G. gallus* (F1NB20-1), *X. laevis* (P55861-1), *D. rerio* (A0A0R4IF65-1), *A. thaliana* (Q9LPD9-1).

E Model highlighting the repositioning of the MCM2 WH domain following Pol ϵ engagement with CMG. Replisome model displayed using cylinder and stub cartoon rendering. The position of the MCM2 WH domain is displayed using surface rendering in the absence of Pol ϵ (Rzechorzek *et al*, 2020) (red) and presence (blue).

F Model visualising the ring-stacking interaction between MCM2 Y821 (blue) and POLE1 F2138 (green). Cryo-EM density is displayed as a transparent surface.

G (Top) Detailed view of the interface between POLE1 and the CDC45 as shown as in (C). (Bottom) Multiple sequence alignment for residues involved in the POLE1/CDC45 interface. Residues that make inter-protein contacts are marked with an asterisk. Uniprot ID for sequences used for POLE1 alignment is as described in (C) and Uniprot IDs used for the CDC45 alignment are as described in Fig EV2D.

H (Top) Detailed view of the interface involving POLE1, POLE2 and MCM5 as shown as in (C). (Bottom) Multiple sequence alignment for residues involved in the POLE1/POLE2/MCM5 interface. Residues that make inter-protein contacts are marked with an asterisk. Uniprot ID for sequences used for POLE1 alignment are as described in panel b. Uniprot IDs used for POLE2 alignment: *H. sapiens* (P56282-1), *S. cerevisiae* (P24482-1), *S. pombe* (O94263-1), *G. gallus* (Q5ZKQ6-1), *X. laevis* (Q9DGB4-1), *D. rerio* (Q8JHG8-1), *A. thaliana* (Q500V9-1). MCM5 alignment: *H. sapiens* (P33992-1), *S. cerevisiae* (P29496-1), *S. pombe* (P41389-1), *G. gallus* (Q5ZKL0-1), *X. laevis* (P55862-1), *D. rerio* (F1QK71-1), *A. thaliana* (O80786-1).

I Low threshold cryo-EM map, displayed as a transparent grey surface, for the complete core human replisome highlighting unmodelled density, in the same location as the MCM5 winged-helix domain in previous *S. cerevisiae* structures, circled with a dashed yellow line.

J Schematic describing the processing pipeline used to obtain a cryo-EM reconstruction containing additional ordered density for the Pol ϵ catalytic module.

© 2021 MRC Laboratory of Molecular Biology.

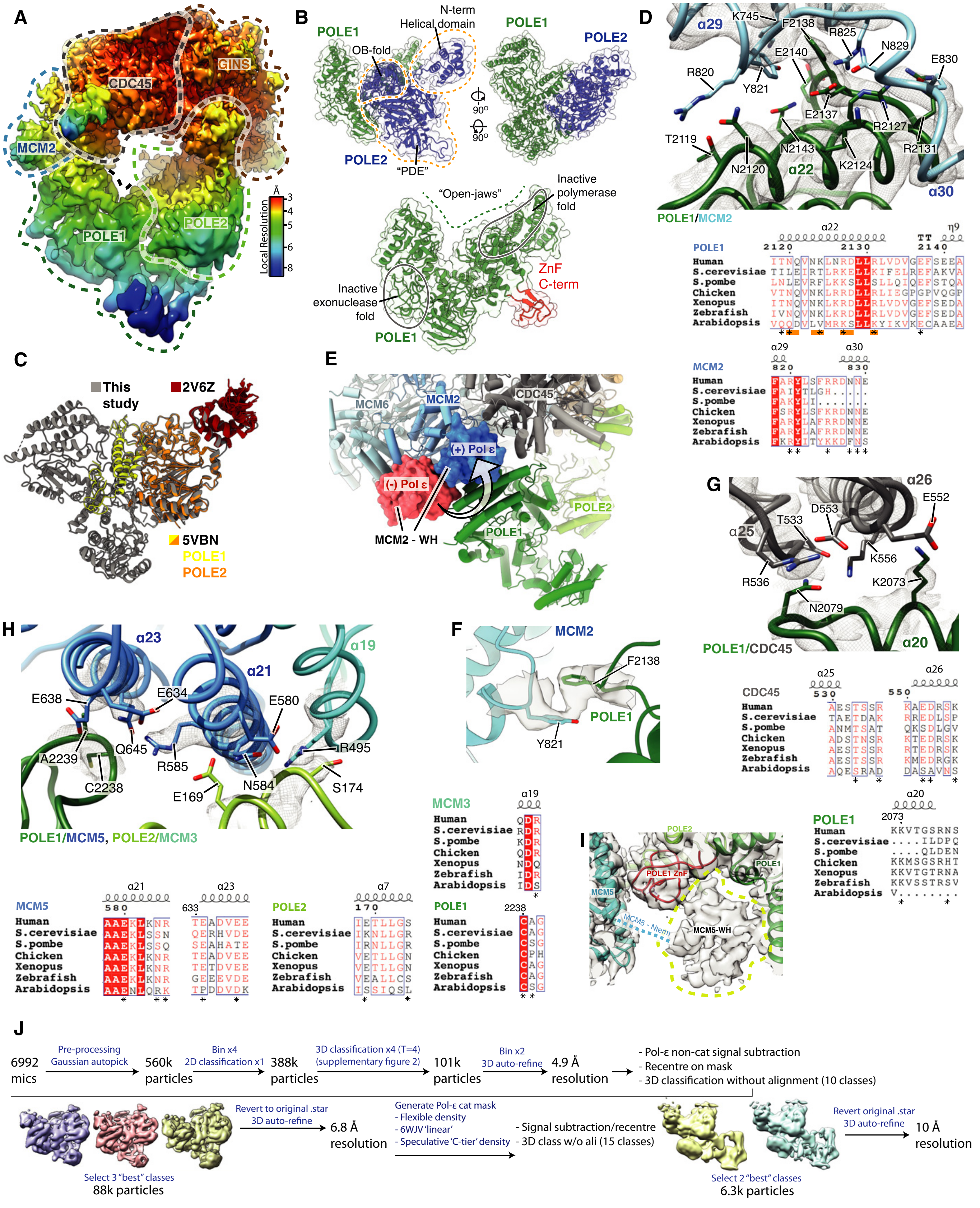

Figure EV4.

© 2021 MRC Laboratory of Molecular Biology.

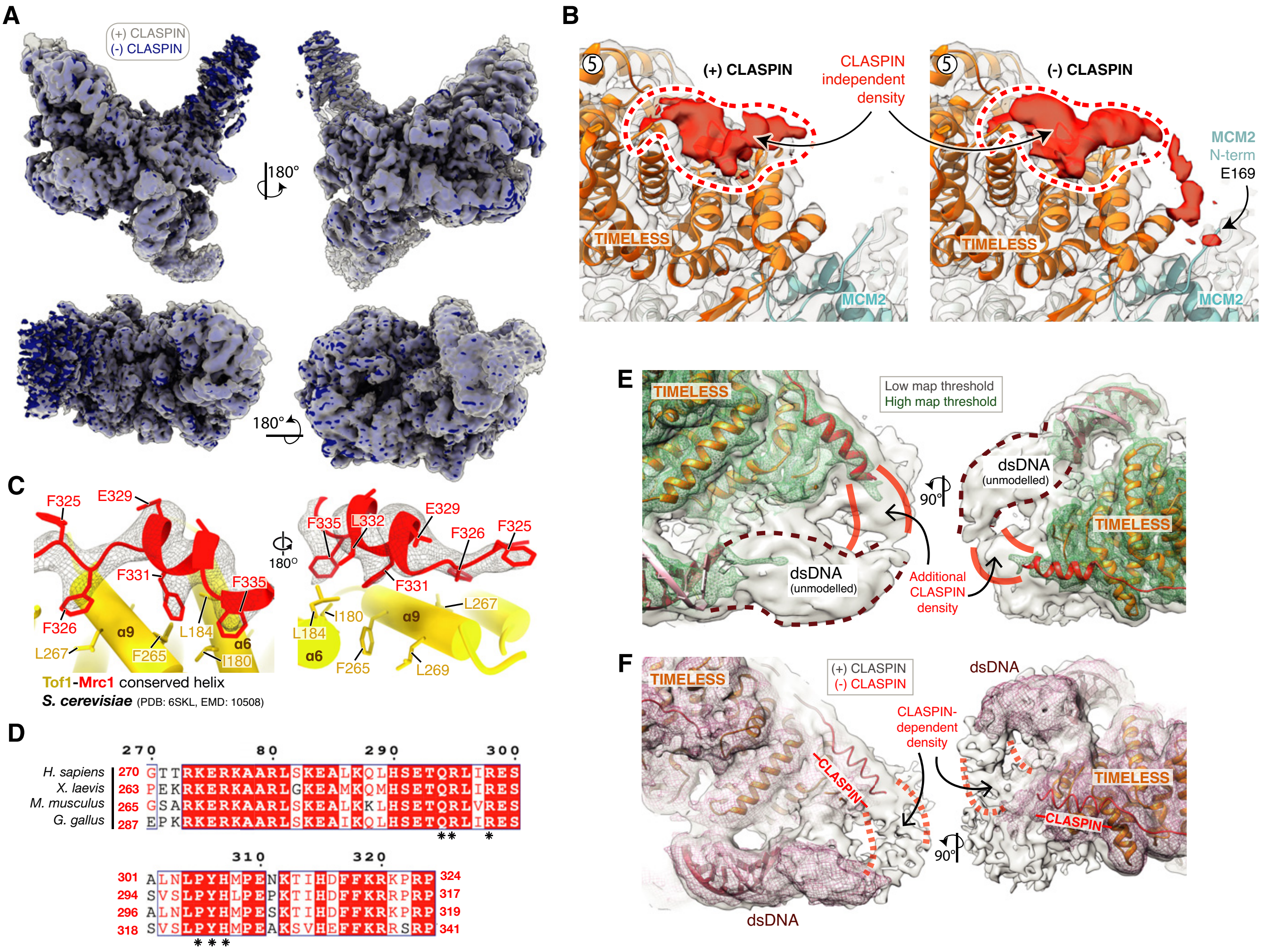

Figure EV5. (Related to Fig 6). Identification of CLASPIN docking sites on the human replisome.

A Cryo-EM reconstruction of the core human replisome in the presence of CLASPIN (grey) rigid-body-docked into the cryo-EM reconstruction in the absence of CLASPIN (blue) using UCSF Chimera (Pettersen *et al*, 2004) (correlation 0.8993).

B Detailed view of the CLASPIN independent density that is bound to the N-terminal end of the TIMELESS α-solenoid. (Left) density in the presence and (right) absence of CLASPIN.

C Closeup view of the interface between Mrc1 residues F335-F325 and Tof1. The Mrc1 model was built into unmodelled density present in the *S. cerevisiae* replisome (PDB: 6SKL, EMD: 10508). Tof1 α6 and α9 are structurally equivalent to TIMELESS helices α8 and α11, respectively. Tof1 and Mrc1 are visualised using cartoon rendering with density assigned to Mrc1 displayed as grey mesh.

D Multiple sequence alignment for *H. sapiens* CLASPIN residues 270–324 at CLASPIN site 1. CLASPIN residues involved in contacting the replisome are marked with an asterisk. Alignments carried out using NCBI Clustal Omega (Sievers & Higgins, 2014) and visualised using ESPrit (Robert & Gouet, 2014) with the primary sequence indicated. Uniprot ID for sequences used for CLASPIN alignment: *H. sapiens* (Q9HAW4-1), *M. musculus* (Q80YR7-1), *G. gallus* (F1P0J7-1), *X. laevis* (Q9DF50-1).

E Cryo-EM map for the core human replisome in the presence of CLASPIN at both high (green mesh) and low (grey transparent surface) thresholds, indicating the presence of density, continuous with CLASPIN site #1, projecting towards the upstream parental DNA duplex.

F Cryo-EM map of the core human replisome in the presence of CLASPIN (grey transparent surface) and absence (red mesh) both at low thresholds. Indicates that density projecting from CLASPIN-dependent site #1 towards the unmodelled parental DNA duplex is dependent upon CLASPIN.

© 2021 MRC Laboratory of Molecular Biology.

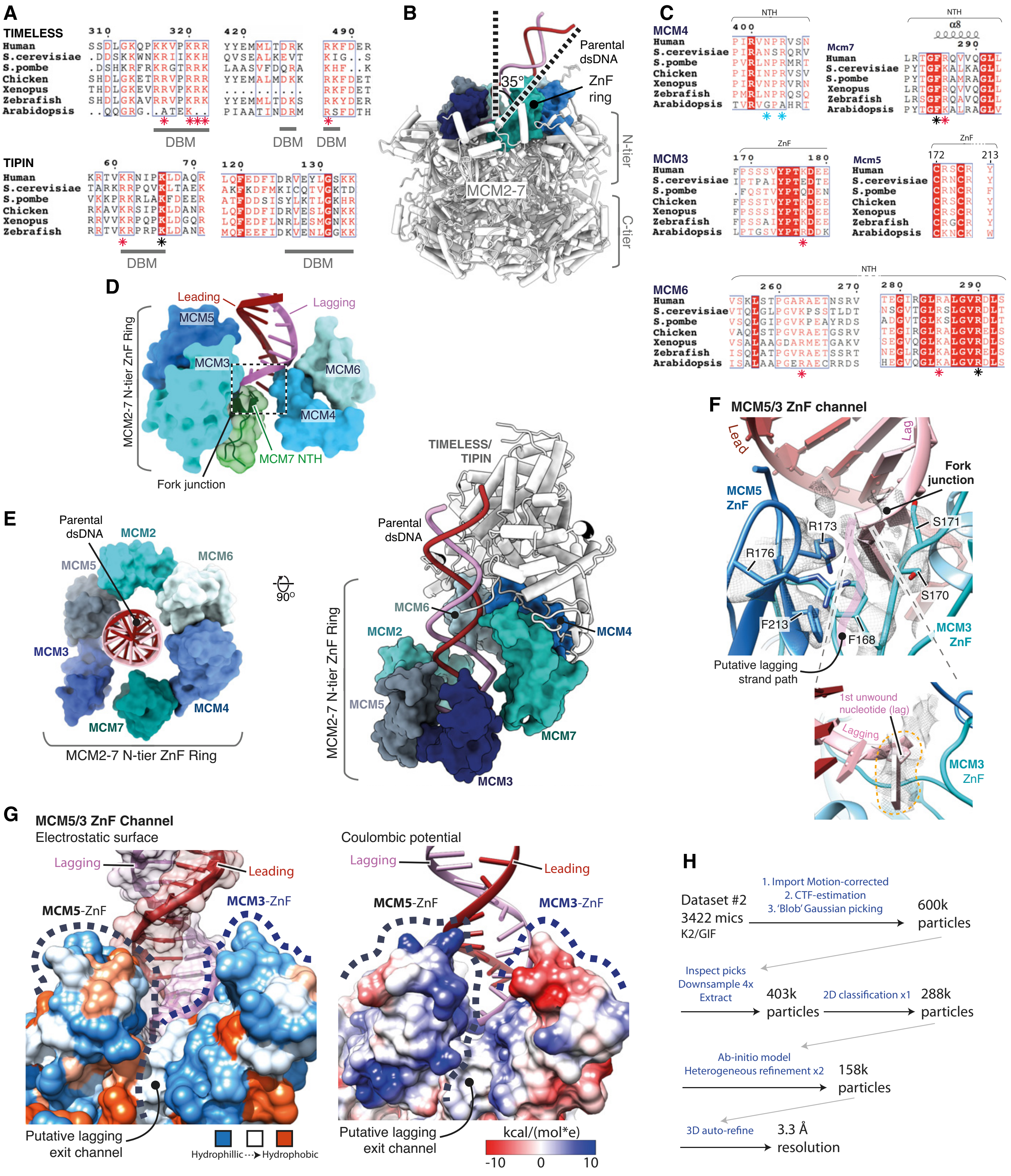

Figure EV6.

 © 2021 MRC Laboratory of Molecular Biology.

Figure EV6. (Related to Fig 7). Interactions between the human replisome and fork DNA.

A Multiple sequence alignment for regions in TIMELESS (top) and TIPIN (bottom) involved in DNA binding. Specific residues seen to be interacting with dsDNA in the structure are demarcated as part of a DNA-binding motif (DBM). Level of conservation for residues contacting dsDNA indicated with a coloured asterisk, red—charge, blue—highly, black—invariant. All alignments in the figure were carried out using NCBI Clustal Omega and visualised using ESPrit with the primary human sequence indicated. Uniprot ID for sequences used for TIMELESS alignment: *H. sapiens* (Q9UNS1-1), *S. cerevisiae* (P53840-1), *S. pombe* (Q9UUM2-1), *G. gallus* (A0A3Q2UKM8-1), *X. laevis* (Q3LGB9-1), *D. rerio* (E7FGL0-1), *A. thaliana* (A0A1P8B9S9-1). TIPIN alignment: *H. sapiens* (Q9BVW5-1), *S. cerevisiae* (Q04659-1), *S. pombe* (O14350-1), *G. gallus* (Q5F416-1), *X. laevis* (Q0IHI4-1), *D. rerio* (Q6DBR4-1).

B Side-on view of the MCM2-7 complex, displayed using pipes and planks, illustrating the angle of the parental dsDNA as it enters the N-tier. The ring of ZnF domains that encircle the incoming duplex is rendered as an opaque surface.

C Multiple sequence alignment for the regions of MCM4, 6, 3, 5, 7 contacting the DNA at the fork junction as shown in (A). Level of conservation for residues contacting DNA at the fork junction indicated with a coloured asterisk, red—charge, blue—highly, black—invariant. Uniprot ID for sequences used for the MCM5 alignment is as described in Fig EV4H. Uniprot IDs used for MCM4 alignment: *H. sapiens* (P33991-1), *S. cerevisiae* (P30665-1), *S. pombe* (P29458-1), *G. gallus* (E1C2U4-1), *X. laevis* (P30664-1), *D. rerio* (Q6NZV2-1), *A. thaliana* (Q0WVF5-1). MCM6 alignment: *H. sapiens* (Q14566-1), *S. cerevisiae* (P53091-1), *S. pombe* (P49731-1), *G. gallus* (Q5ZKR8-1), *X. laevis* (Q5FWY4-1), *D. rerio* (A0A0E4AYA7-1), *A. thaliana* (F4KAB8-1). MCM7 alignment: *H. sapiens* (P33993-1), *S. cerevisiae* (P38132-1), *S. pombe* (O75001-1), *X. laevis* (Q91876-1), *D. rerio* (Q7ZVL6-1), *A. thaliana* (P43299-1). MCM3 alignment: *H. sapiens* (P25205-1), *S. cerevisiae* (P24279-1), *S. pombe* (P30666-1), *X. laevis* (P49739-1), *D. rerio* (A0A0E4AY38-1), *A. thaliana* (Q9FL33-1).

D MCM2-7 N-tier loops contacting the dsDNA at the fork junction. Model rendered as an opaque surface, with the MCM2-7 NTH also displayed as a cartoon with transparent surface rendering.

E Two views of the MCM2-7 N-tier secondary ZnF domain ring, which encircles the incoming parental dsDNA duplex. The ZnF models are displayed using opaque surface rendering with TIMELESS-TIPIN and dsDNA visualised as a cartoon using pipes and planks rendering.

F Detailed view of the putative lagging-strand exit channel between the MCM3 and MCM5 ZnF's (Top). Model visualised using cartoon rendering with selected side chains displayed and their corresponding cryo-EM density represented as a transparent mesh. Focussed view highlighting the first lagging-strand nucleotide following strand separation and its corresponding cryo-EM density (Bottom).

G MCM5/3 ZnF channel displayed using surface rendering, coloured according to electrostatic potential (left) and Coulombic potential (right) (Pettersen *et al*, 2004) according to their respective inset keys.

H Schematic pipeline diagram describing the processing of 3422 micrographs from cryo-EM collection #1 in the presence of CLASPIN using CryoSPARC (Punjani *et al*, 2017). This processing resulted in a reconstruction in which density was identified between the MCM5 and MCM3 ZnF's that is continuous with the lagging strand at the fork junction.

© 2021 MRC Laboratory of Molecular Biology.
