# Supplementary material for: Structure of a human replisome shows the organisation and interactions of a DNA replication machine
Source: EMBO J. 2021 Oct 25;40(23):e108819. doi: 10.15252/embj.2021108819 (PMC8634136; doi:10.15252/embj.2021108819)
Supplement: Supplementary file 3 [file EMBJ-40-e108819-s003.pdf]

THE EMBO JOURNAL

Structure of a human replisome shows the organisation and interactions of a DNA replication machine

Morgan Jones, Yasemin Baris, Martin Taylor, and Joseph Yeeles
DOI: 10.15252/embj.2021108819

Corresponding author(s): Joseph Yeeles (jyeeles@mrc-lmb.cam.ac.uk)

Review Timeline:

Submission Date:	27th May 21
Editorial Decision:	2nd Jul 21
Revision Received:	26th Aug 21
Editorial Decision:	24th Sep 21
Revision Received:	28th Sep 21
Accepted:	30th Sep 21

Editor: Hartmut Vodermaier

Transaction Report:

(Note: With the exception of the correction of typographical or spelling errors that could be a source of ambiguity, letters and reports are not edited. Depending on transfer agreements, referee reports obtained elsewhere may or may not be included in this compilation. Referee reports are anonymous unless the Referee chooses to sign their reports.)

1st Editorial Decision 2nd Jul 2021

Thank you for submitting your manuscript on human replisome structure for our editorial consideration. I have now received reports from three expert referees, copied below for your information. As you will see, all three reviewers acknowledge the general interest and technical quality of the structural work, but raise a number of specific concerns related mainly to the presentation and to insufficient experimental validation of structural results. Pending adequate improvement of these issues, we would be happy to pursue a revised manuscript further for EMBO Journal publication. Key points for such a revision would be better focussing the structural descriptions and adding more comparison to various other related full/partial replisome structures reported recently; and inclusion of additional biochemical/mutational data, e.g. for helicase activity or Claspin localization, as suggested in the reports.

Given that the key points are generally shared by all referees and well explained in the reports, I will not repeat them in detail here; but please do not hesitate to contact me early on in case you would like to further clarify any referee requests or revision requirements; as carefully answering to all referee points at the time of resubmission is particularly important in light of our policy to allow only a single round of major revision. We would also be happy to grant an extension of the default three-months revision period if needed.

Detailed information on preparing and uploading a revised manuscript can be found below, and in our Guide to Authors. Thank you again for the opportunity to consider this work for The EMBO Journal, and I look forward to your revision.

Referee #1:

In this manuscript, Jones et al reported a cryo-EM structure of a human replisome core consisting of CMG helicase, leading strand polymerase Pol epsilon, and four other accessory factors. This structure provides a framework for the organization of these human replisome components and a wealth of information about the detailed interactions among them. Technically, the data presented here is sound and underscores the conservation in organization between the yeast and human replisome. While the litany of structural features described here is important for understanding the replisome organization, it seems fragmented and does not integrate well with a focal point to address some of the key issues of the human replisome. A major concern is the lack of functional validation of the structure. There are other concerns that need to be addressed.

Major points:

1. The human replisome reconstituted in this work was assembled on the core complex, the CMG helicase. In 2020, Rzechorzek et al determined a structure of the human CMG complex in complex with And-1. However, this work was not properly referenced nor sufficiently discussed in the introduction.

2. In contrast to the work of Rzechorzek et al 2020 in which human CMG complex was purified from cultured human cells, all of the components for replisome assembly here were isolated from insect cells. The authors should provide evidence showing that these recombinant components, for example CMG helicase and Poε, and the assembly are active in duplex DNA unwinding and DNA synthesis.

3. As MCM2-7 genes are highly conserved across species and their expression levels are high, it is likely that some MCM proteins of insect origin may be incorporated into hMCM2-7 assemblies and co-purified. This heterogeneity might be the explanation for the difference in conformations between the structures observed in Rzechorzek et al 2020 and this work. It would be useful if the authors could establish the degree of compositional homogeneity of the recombinant hMCM2-7.

4. The structures of hCMG between Rzechorzek et al 2020 and this work should be compared more carefully in order to reveal the conformational changes induced by the docking of Polε, And-1, and Timeless-Tipin. The authors mentioned ssDNA binding in the inner chamber of MCM ring appears different in the two studies. This variation in CMG conformation should be analyzed in depth to see if the helicase could be reconfigured by Pol ε and other factors.

5. On page 6 line 22-23, it is stated that This configuration is very similar to one of the three conformations (conformation 1) we observed for scCMG bound to fork DNA, the FPC and Ctf4 in the presence of AMP-PNP (Baretić et al., 2020). Why was only one conformation identified for the human replisome? It would be informative if the authors employ the strategy they used before to perform more elaborate classifications and refinements focusing on the DNA. Additional conformers may emerge. This kind of information will further our understanding of how the CMG helicase copes with other engines to engage with DNA.

6. The authors identified some regions of unassigned densities and attributed them to Claspin as these densities disappear in the absence of Claspin. However, there is an alternative explanation to this observation. These densities may be attributed to other floppy replisome components, which becomes structured upon Claspin binding. Additional assays should be used to confirm the docking sites for Claspin.

7. On page 15, the authors concluded that Claspin adopts an extended and flexible configuration stretching across one side of human replisome likely involving more than one copy of Claspin. The stoichiometry of Claspin in the protein gel in Fig. 1B does not support this notion. More discussion on the flexible nature and stoichiometry of Claspin would be helpful here.

8. For Polε structure, some crystal and NMR structures have been determined for POLE1 and POLE2. It is not clear if the

relevant structural information were utilized for modelling. On page 10, the authors only mentioned rigid body docking of I-TASSER models. If the modelling process involves those structures, it should be properly referenced. In addition, structural comparison should also be conducted between Polε structure determined here and the crystal structure (Baranovskiy et al, 2017) to understand if CMG binding modulates Polε conformation.
For example, the NMR structure of N-terminal helical domain of POLE2 (PDB: 2v6z) has been previously determined (Nuutinen et al 2008). It is not clear how similar these structures are. The authors should include this information in the manuscript.

9. The overall resolution of Poε including the interfaces between hCMG and Polε is quite low. As shown in the figure, the densities for side chains of a number of residues at those interfaces are not obvious. So, it is tenuous to draw conclusion on the nature of the interactions involving side chains. This low resolution also reflects the interactions at the interfaces between CMG and Polε maybe relatively transient and/or dynamic. The tone of this part should be softened.

10. On page 12, the authors claimed that Polε alters the configuration of MCM active sites. It should be further expanded to provide insights into the function of Polε in modulating CMG activities. Structural comparison among available hCMG structures should be conducted to have a thorough understanding of the structural changes in MCM ring upon Polε binding.

11. It is interesting to know that an invariant phenylalanine (F285) of Mcm7 stacks against the final base pair. Based on this observation, the authors proposed an unwinding mechanism. If it is true, it will be a major contribution to the field. The authors should consider including an in vitro helicase assay to test this hypothesis.

Minor points

1. on page 6 line 12, "The conformations of the MCM N-tier, Cdc45 and GINS are very similar to those observed for isolated hCMG (Rzechorzek et al., 2020) (Fig 3A)." The figure reference should be Fig EV3A.

2. on page 3 line 3-4, there are some typos. It should be revised as "As the leading-strand template is pulled through the MCM central pore, the lagging-strand template is excluded (Fu et al, 2011).
On page 9 line 14, "serval" should be "several".

3. In all of the figures, the EM densities are not superimposed on the atomic model making it difficult to evaluate the quality of the map.

4. The order of figure 2 and 3 should be reversed to match their order of appearance in the text.

Referee #2:

This manuscript by Jones and coauthors reports the 3.2A cryo-EM structure of the human fork progression complex bound to DNA polymerase epsilon, an assembly at the core of the replisome that is responsible for DNA replication. With the exception of claspin and the catalytic domain of pol e, all components are well resolved in the structure, allowing the authors to build models of the various human protein components and analyse how they bind each other. This work adds to a list of structures of core replisome components from yeast, fly, and humans, including the yeast fork progression complex, the human CMG-And-1 complex, the yeast CMG-pol e complex, among others. The human core replisome structure presented by the authors recapitulates many of the findings observed in previous structures (especially of the yeast fork progression complex) but also highlights some interesting differences, for example how binding of timeless to the CMG differs between yeast and humans. Although it is important to show that similar concepts observed in yeast apply to the human replisome, the manuscript falls short of testing at least some of the structural conclusions biochemically. Including such data would support the importance of the observed interactions for function and strengthen the manuscript.

Other comments:

Generally, the manuscript is well written, although at times it is easy to get lost in the very detailed descriptions of the structure and contacts at the various interfaces. Streamlining the description of the structure would benefit the manuscript and make it more accessible to readers that are not structural biologists.

Figures (general):
The figure panels are not always presented in order, which at times is confusing and makes it difficult to spot the correct panel. The font sizes used in several panels are also very small, which makes it challenging to read certain labels and the alignments.

Page 2, line 8:
The authors should acknowledge the work of the Pellegrini group here, who have determined the first snapshot of the human CMG bound to fork DNA (Rzechorzek et al, NAR 2020). The next sentence (lines 9-10) is also somewhat misleading: the human CMG structure has been determined without and with And-1, which represents a major complex at the core of the replisome.

Page 3, line 2:
Rzechorzek et al (NAR 2020) proposed a sequential rotary model for ATP hydrolysis and DNA translocation based on the structure of the human CMG and should be acknowledged here as well.

Fig. EV3B:
This panel is quite busy and it is hard to distinguish the two maps. It would help to show the models of both structures with only one of the EM maps.

Fig. EV4C:
Regions corresponding to And-1, Cdc45 and GINS in this map should be labelled.

Fig. EV4D:
The authors should highlight the residues in the alignment (and all other alignments) that are directly involved in interactions at the various interfaces and that are shown with side chains in Fig. 2.

Fig. 3:
The authors describe the interactions of timeless-tipin with the CMG in great detail, but the importance of the individual interactions is not immediately apparent. How is CMG unwinding activity and DNA synthesis affected when wedge, anchor, bridge, and DBM of timeless are mutated? Are these regions equally important for recruitment of timeless-tipin to the CMG? This section of the manuscript would greatly benefit from some mutational analysis and biochemistry to support the significance of the interactions that are described in detail.

Fig. 6D:
The authors assign polarity to the helix in claspin-dependent density 2. In the figure, the density does not appear to be resolved well enough to assign N and C-termini. The authors should explain how they came to their conclusion.

Fig. 7:
The authors point out that an invariant phenylalanine in Mcm7 serves as a separation pin during DNA strand separation. What effect do mutations of this residue have on CMG helicase activity and DNA synthesis? This should be tested experimentally.

Page 11, line 15: Fig. 6C should probably be EV6C.

PDB validation reports are missing and should be included with the manuscript submission.

Referee #3:

The authors provide a structural insight into the human replisome. This expands on the work by Rzechorzek et al, as it incorporates additional factors (Timeless-Tipin, Claspin) and achieves a higher resolution. The overall structure is consistent with work in yeast by the same group, but provide new structural insights about PolE and Claspin attachment. The structural description is in part very detailed and could be slightly shortened (see below), but seems to be fairly presenting results in the context of previously published work.

Major comments:
The description of the PolE Non-catalytic interface with the CMG is fairly descriptive. Either shorten it, or add mutants to verify some of the results.

Minor comments:
It would be informative to illustrate how much of each protein was modelled in form of a protein chain overview, and to show this in comparison to other structures from yeast and human.

The authors state on page 7, lines 9-12 "Although this suggests the majority of Claspin is not bound stably, several regions of density that could not be unambiguously modelled are visible (Fig 1C). These regions may therefore represent Claspin docking sites and we address this in a subsequent section". This is not clear - where are these regions?

It is mentioned on page 8 line 14/15 that "there is poor sequence conservation between human and S.cerevisiae (Fig EV4D)". Is this also true for the specific amino acids making contact?

The authors mention "POLE1nonCat adopts a polymerase fold with a wide-open jaw and C-terminal zinc finger (Fig 4B)". However, this is not clear from the figure (both open jaw and zinc finger location).

The authors state "Whilst the resolution of docking site 1 is insufficient to unambiguously assign side-chain rotamers, the pattern of charged residues on the face of the POLE1 α22 helix interacting with Mcm2 is well conserved (Fig 6C)." I am not sure if Figure 6C shows this - maybe EV6C?

The authors state "This interface is locked in place through a ring-stacking interaction between the well conserved F2138 of POLE1 and invariant Y821 of Mcm2". It is unclear which figure shows this - maybe EV6C - but here F2138 is labelled as P2138 - so this is confusing.

The authors suggest that Pol E is involved in the rate of replisome progression. However, the absence of DNA and flexibility in Mcm2 would rather argue for a release of the complex from DNA. Therefore, this statement should be corrected accordingly (this is better argued in the discussion).

Figure 6: The authors speculate that region 5 belongs to Mcm2. I think it would be better to remove this statement or alternatively make an experiment to proof the point. The whole section is too speculative and should be deleted.

The authors describe the location of Claspin, but little functional relevance is mentioned. How could this be enhanced? (the discussion provides more info in this respect).

The DNA binding analysis is interesting. Would be possible to highlight the most conserved amino acids in Figure 7CDEF e.g. with an asterisk or changing the colour of the font. Also, Figure 7H is nice, but could benefit from a zoomed out view - to understand the lagging strand channel in the context of the entire complex.

In the discussion the authors suggest a role for Claspin in limiting CMG backtracking and slowing PolE during replisome progression and connecting this to cancer treatment. It would be sensible to reference this to a figure, to make the link stronger. It would be exciting to test this model by mutagenesis, but this appears not essential for publication.

In the discussion the authors suggest that Pol ε can modulate C-tier configuration and therefore potentially CMG helicase activity. I think it is not clear if this is due to PolE activity, due to failed complex assembly or due to the effect of a different protein, so it would be good to revise the statement accordingly, or provide data that support the statement.

Referee #1:

In this manuscript, Jones et al reported a cryo-EM structure of a human replisome core consisting of CMG helicase, leading strand polymerase Pol epsilon, and four other accessory factors. This structure provides a framework for the organization of these human replisome components and a wealth of information about the detailed interactions among them. Technically, the data presented here is sound and underscores the conservation in organization between the yeast and human replisome. While the litany of structural features described here is important for understanding the replisome organization, it seems fragmented and does not integrate well with a focal point to address some of the key issues of the human replisome. A major concern is the lack of functional validation of the structure. There are other concerns that need to be addressed.

Major points:
1. The human replisome reconstituted in this work was assembled on the core complex, the CMG helicase. In 2020, Rzechorzek et al determined a structure of the human CMG complex in complex with And-1. However, this work was not properly referenced nor sufficiently discussed in the introduction.

We apologise that this work from the Pellegrini lab did not feature more prominently at the beginning of our introduction. In the revised manuscript we have additionally cited the Rzechorzek paper at the beginning of the introduction (Page 2, lines 7-9) and added a new sentence highlighting the working in relation to helicase translocation mechanism (Page 3, lines 2-3).

2. In contrast to the work of Rzechorzek et al 2020 in which human CMG complex was purified from cultured human cells, all of the components for replisome assembly here were isolated from insect cells. The authors should provide evidence showing that these recombinant components, for example CMG helicase and Pol ε, and the assembly are active in duplex DNA unwinding and DNA synthesis.

In a separate project (Yeeles lab unpublished data) we have reconstituted leading- and lagging-strand DNA replication at cellular rates entirely with purified human proteins. This work uses proteins that were expressed and purified in the same way as the proteins in the current manuscript. Therefore, we know that all our protein preparations are active. In our view this work represents a major advance in the field and, once it is completed, will be published elsewhere as a standalone study.

As we cannot include any of the functional data described above, to address the Referee's comment, in the revised manuscript we include a DNA helicase assay with purified human CMG and DNA polymerase assay with Pol ε, PCNA, RFC and RPA (Appendix Figure S1A and B). These assays clearly demonstrate that human CMG and Pol ε purified from insect cells are active.

3. As MCM2-7 genes are highly conserved across species and their expression levels are high, it is likely that some MCM proteins of insect origin may be incorporated into hMCM2-7 assemblies and co-purified. This heterogeneity might be the explanation for the difference in conformations between the structures observed in Rzechorzek et al 2020 and this work. It would be useful if the authors could establish the degree of compositional homogeneity of the recombinant hMCM2-7.

When the Referee refers to differences in conformation we assume they are referring to ATPase site occupancy and ssDNA engagement in the MCM C-tier. For several reasons we think contamination with insect proteins is not the reason for these differences. Firstly, it is important to emphasise that

prior CMG structures have observed similar conformations to both the Rzechorzek structure and our structure. Importantly, a paper from Alessandro Costa's lab, that imaged drosophila CMG following translocation in ATP (Eickhoff et al, 2019), captured several conformations, one similar to the Rzechorzek paper and one similar to our paper. Therefore, both conformations are likely to reflect distinct steps on the translocation cycle. We have added a sentence emphasising this in the results (Page 7, lines 7-10). Secondly, there are many differences in the way samples were prepared between the Rzechorzek paper and our paper e.g. the presence of Pol ε, TIMELESS-TIPIN, CLASPIN and AND-1; the use of different nucleotides (AMP-PNP or ATP-gamma-S); GraFix. Any one of these variables could have influenced which conformation was observed. Again, importantly, because both configurations are reminiscent of conformations observed during CMG translocation in ATP (Eickhoff et al, 2019) we do not consider this to be a major issue. Thirdly, we have analysed our cryo-EM maps, specifically focusing on regions of density where the amino acid sequence clearly differs between moth (Trichoplusia ni) and human for Mcm5, which is the MCM subunit expressed at the lowest level. In these regions the density clearly matches the human Mcm5 sequence and is of equivalent quality to neighbouring regions, suggesting that there is therefore not considerable heterogeneity due to contaminating insect proteins.

4. The structures of hCMG between Rzechorzek et al 2020 and this work should be compared more carefully in order to reveal the conformational changes induced by the docking of Polε, And-1, and Timeless-Tipin. The authors mentioned ssDNA binding in the inner chamber of MCM ring appears different in the two studies. This variation in CMG conformation should be analyzed in depth to see if the helicase could be reconfigured by Pol ε and other factors.

Related to point 3, the major difference between the Rzechorzek conformation and our structures is ATPase site occupancy and ssDNA engagement. By comparison with the conformations of translocating CMG from the Costa lab (Eickhoff et al, 2019), both are likely to be representative of translocation intermediates. Because DNA is bound on opposite sides of MCM ring in our structure compared to the Rzechorzek structure, the C-tier of MCM is in a very different configuration with different subunits binding ssDNA and different ATPase sites occupied. In our view, these considerable differences - that have been seen in drosophila CMG in the absence of accessory factors (Eickhoff et al, 2019) - preclude a detailed comparison of our structure and the Rzechorzek structure to identify changes to the MCM C-tier that may be induced by the additional replisome factors present in our structure. Furthermore, because the CMG conformation in our structure, specifically the MCM C-tier, is similar to one of the translocating conformations (Eickhoff et al, 2019), the conformation is very unlikely to be dependent on the additional factors present in our structure. It is our view that in order to unambiguously assign conformational changes dependent on Pol ε, AND-1 and TIMELESS-TIPIN, a structure of isolated CMG prepared in the same way with DNA bound to the MCM2/3/5 side of the ring would be required and such a structure has not yet been obtained.

5. On page 6 line 22-23, it is stated that This configuration is very similar to one of the three conformations (conformation 1) we observed for scCMG bound to fork DNA, the FPC and Ctf4 in the presence of AMP-PNP (Baretić et al., 2020). Why was only one conformation identified for the human replisome? It would be informative if the authors employ the strategy they used before to perform more elaborate classifications and refinements focusing on the DNA. Additional conformers may emerge. This kind of information will further our understanding of how the CMG helicase copes with other engines to engage with DNA.

We do not know why only one conformation for the CMG C-tier was observed in our dataset, although this is certainly not without precedent. Indeed, our prior yeast replisome structure (Baretić et al., 2020) was the first example of a yeast replisome complex assembled in the presence of a non-hydrolysable nucleotide analogue (AMP-PNP) to display multiple conformations for the MCM C-tier. This structure was solved in the absence of Pol ε, and we speculate that its binding stabilises the MCM C-tier, favouring a single DNA engagement conformation. In addition, four previously reported datasets found ssDNA bound on only one side of the MCM ring (Georgescu et al., 2017, Goswami et

al., 2018, Yuan et al., 2019, Rzechorzek et al., 2020). We have of course performed extensive sub-classification of our human replisome data sets and we have several more human replisome data sets related to separate projects: in all cases we observe a single conformation of the C-tier with DNA engaged on the MCM2/3/5 side of the complex. It is our view that, in order to directly address human CMG translocation mechanism, structures of translocating human CMG prepared in the presence of ATP will be required and this is beyond the scope of this work.

6. The authors identified some regions of unassigned densities and attributed them to Claspin as these densities disappear in the absence of Claspin. However, there is an alternative explanation to this observation. These densities may be attributed to other floppy replisome components, which becomes structured upon Claspin binding. Additional assays should be used to confirm the docking sites for Claspin.

We agree it is possible that CLASPIN could stabilise another protein such that it could contribute to the Claspin-dependent densities. However, we considered it extremely unlikely that ordering of other proteins accounted for all four Claspin-dependent densities.

By taking advantage of the newly released AlphaFold structure predictions for human Claspin and yeast Mrc1, we have been able to build an atomic model for CLASPIN that encompasses three regions of CLASPIN-dependent density that we were previously unable to assign. We have also been able to re-evaluate our published yeast cryo-EM map (Baretic et al., 2020) to unambiguously assign sequence to a region of Mrc1. This analysis demonstrates that 3 of the 4 CLASPIN-dependent densities are indeed contributed by CLASPIN, density 4 being of insufficient resolution to unambiguously assign, and identifies specific binding sites for CLASPIN/Mrc1 in the replisome for the first time. Moreover, comparison of our new atomic model for CLASPIN with published functional data, indicates that the CLASPIN binding sites we have discovered are functionally significant. In light of this new analysis, the section of the revised manuscript with the subheading “Claspin binding in the human replisome” (Page 13, line 21 – Page 17, line 9) and revised Figures 6 and EV5 have been significantly reworked from the original manuscript.

7. On page 15, the authors concluded that Claspin adopts an extended and flexible configuration stretching across one side of human replisome likely involving more than one copy of Claspin. The stoichiometry of Claspin in the protein gel in Fig. 1B does not support this notion. More discussion on the flexible nature and stoichiometry of Claspin would be helpful here.

We are a little confused by this comment. It is certainly not our conclusion that multiple Claspin molecules are present in the replisome. Rather, based on the silver-stained gel (Figure 1B) we think Claspin is present as a single copy in the replisome that has an extended and flexible conformation. For clarity we have added a sentence stating that we think there is a single copy of CLASPIN in the replisome (Page 16, lines 3 and 4).

8. For Polε structure, some crystal and NMR structures have been determined for POLE1 and POLE2. It is not clear if the relevant structural information were utilized for modelling. On page 10, the authors only mentioned rigid body docking of I-TASSER models. If the modelling process involves those structures, it should be properly referenced. In addition, structural comparison should also be conducted between Polε structure determined here and the crystal structure (Baranovskiy et al, 2017) to understand if CMG binding modulates Polε conformation.
For example, the NMR structure of N-terminal helical domain of POLE2 (PDB: 2v6z) has been previously determined (Nuutinen et al 2008). It is not clear how similar these structures are. The authors should include this information in the manuscript.

Two of the top threading templates used to generate homology models for the non-cat domain of Pol ε were indeed structures of the human N-terminal helical domain of POLE2 (2V6Z) and a complex with a C-terminal region of human POLE1 and POLE2 (5VBN). We have subsequently acknowledged this within the model building section of the methods (Page 40, lines 9-11). We have also commented on the levels of structural homology between these input models and the final, refined model for Pol ε through RMSD analysis on page 40, lines 16-17.

9. The overall resolution of Poε including the interfaces between hCMG and Polε is quite low. As shown in the figure, the densities for side chains of a number of residues at those interfaces are not obvious. So, it is tenuous to draw conclusion on the nature of the interactions involving side chains. This low resolution also reflects the interactions at the interfaces between CMG and Polε maybe relatively transient and/or dynamic. The tone of this part should be softened.

We have amended the description for the docking site 1 interface (page 11, lines 13-25) to remove discussion of specific side-chain contacts in favour of a comment on the general electrostatic nature of this interface. We only describe side-chain contacts between F2138 of POLE1 and Y821 of MCM2, for which there is unambiguous density.

10. On page 12, the authors claimed that Polε alters the configuration of MCM active sites. It should be further expanded to provide insights into the function of Polε in modulating CMG activities. Structural comparison among available hCMG structures should be conducted to have a thorough understanding of the structural changes in MCM ring upon Polε binding.

Related to points 3 and 4, the only published human CMG structure is from Rzechorzek et al., 2020, and in that structure the MCM C-tier is in a completely different conformation because ssDNA is bound on the opposite side of the ring. Currently there are no other structures (other than the ones presented in our work) of human CMG. Because DNA is bound on the opposite side of the MCM ring in the Rzechorzek structure compared to our structure, it would in our view be inappropriate to draw conclusions related to Pol ε mediated conformational changes based on a comparison of the two structures.

11. It is interesting to know that an invariant phenylalanine (F285) of Mcm7 stacks against the final base pair. Based on this observation, the authors proposed an unwinding mechanism. If it is true, it will be a major contribution to the field. The authors should consider including an in vitro helicase assay to test this hypothesis.

[AT THE AUTHORS' REQUEST, THE RESPONSE TO THIS REFEREE QUERY IS NOT INCLUDED IN THIS TRANSCRIPT, AS IT REFERS IN DETAIL TO ONGOING UNPUBLISHED RESEARCH OF THEIRS]

Minor points

1. on page 6 line 12, "The conformations of the MCM N-tier, Cdc45 and GINS are very similar to those observed for isolated hCMG (Rzechorzek et al., 2020) (Fig 3A)." The figure reference should be Fig EV3A.

Thank you for spotting this.

2. on page 3 line 3-4, there are some typos. It should be revised as "As the leading-strand template is pulled through the MCM central pore, the lagging-strand template is excluded (Fu et al, 2011).
On page 9 line 14, "serval" should be "several".

Thank you for spotting this.

3. In all of the figures, the EM densities are not superimposed on the atomic model making it difficult to evaluate the quality of the map.

We haven't shown EM density in all figures because, for many of them, the figure would become too busy. Where possible we tried to show density in figures where we show amino acid side chains and we have included maps coloured according to local resolution to illustrate the quality of the EM maps that were used for model building.

4. The order of figure 2 and 3 should be reversed to match their order of appearance in the text.

We think the figure ordering is correct. There was an error in the figure citations which meant that Fig 3 was cited before Fig 2, but this should actually have read Fig EV3.

Referee #2:

This manuscript by Jones and coauthors reports the 3.2A cryo-EM structure of the human fork progression complex bound to DNA polymerase epsilon, an assembly at the core of the replisome that is responsible for DNA replication. With the exception of claspin and the catalytic domain of pol e, all components are well resolved in the structure, allowing the authors to build models of the various human protein components and analyse how they bind each other. This work adds to a list of structures of core replisome components from yeast, fly, and humans, including the yeast fork progression complex, the human CMG-And-1 complex, the yeast CMG-pol e complex, among others. The human core replisome structure presented by the authors recapitulates many of the findings observed in previous structures (especially of the yeast fork progression complex) but also highlights some interesting differences, for example how binding of timeless to the CMG differs between yeast and humans. Although it is important to show that similar concepts observed in yeast apply to the human replisome, the manuscript falls short of testing at least some of the structural conclusions biochemically. Including such data would support the importance of the observed interactions for function and strengthen the manuscript.

As described above (response to Referee #1, point 6), in the revised manuscript we now present an atomic model for regions of CLASPIN in the replisome, showing for the first-time details of how CLASPIN/Mrc1 is attached in the replisome. This has enabled us to re-evaluate several prior studies that examined the function of different regions of CLASPIN. This analysis shows that the CLASPIN binding sites we identify are, as expected, functionally significant. We think this additional work significantly strengthens the manuscript in terms of highlighting the functional importance of the interactions our work has revealed.

Other comments:

Generally, the manuscript is well written, although at times it is easy to get lost in the very detailed descriptions of the structure and contacts at the various interfaces. Streamlining the description of the structure would benefit the manuscript and make it more accessible to readers that are not structural biologists.

We acknowledge that the structural descriptions are rather dense in places and have streamlined some of the descriptions as suggested, particularly the section on Pol ε.

Figures (general):
The figure panels are not always presented in order, which at times is confusing and makes it difficult to spot the correct panel. The font sizes used in several panels are also very small, which makes it challenging to read certain labels and the alignments.

We feel that we have organised the figure panels as best as we can to accommodate all the required information within reasonably size figures. In our revised submission we have ensured labels are appropriately sized.

Page 2, line 8:
The authors should acknowledge the work of the Pellegrini group here, who have determined the first snapshot of the human CMG bound to fork DNA (Rzechorzek et al, NAR 2020). The next sentence (lines 9-10) is also somewhat misleading: the human CMG structure has been determined without and with And-1, which represents a major complex at the core of the replisome.

Again, we apologise that this manuscript did not feature more prominently at the start of our introduction. This has been addressed in the revised manuscript (please see response to Referee #1, point 1).

Page 3, line 2:
Rzechorzek et al (NAR 2020) proposed a sequential rotary model for ATP hydrolysis and DNA translocation based on the structure of the human CMG and should be acknowledged here as well.

The sequential rotary model was proposed by Eickhoff et al 2019 based on structures of drosophila CMG in the presence of ATP. The Rzechorzek paper state that there are elements of their structure that are consistent with a sequential rotary mechanism. Accordingly, we have added a statement saying that the structure of human CMG in ATP-gamma-S is consistent with a sequential rotary mechanism (Page 3, lines 1-3).

Fig. EV3B:
This panel is quite busy and it is hard to distinguish the two maps. It would help to show the models of both structures with only one of the EM maps.

It is important to compare the density here to show that it is essentially the same between the two different EM maps in the region where the models differ. However, we agree that the original figure was too busy and have replaced it with a better example in the revised manuscript (Figure EV1B).

Fig. EV4C:
Regions corresponding to And-1, Cdc45 and GINS in this map should be labelled.

We have highlighted the different proteins as suggested.

Fig. EV4D:
The authors should highlight the residues in the alignment (and all other alignments) that are directly involved in interactions at the various interfaces and that are shown with side chains in Fig. 2.

We thank the Referee for this suggestion and have modified the revised manuscript accordingly.

Fig. 3:
The authors describe the interactions of timeless-tipin with the CMG in great detail, but the importance of the individual interactions is not immediately apparent. How is CMG unwinding activity and DNA synthesis affected when wedge, anchor, bridge, and DBM of timeless are mutated? Are these regions equally important for recruitment of timeless-tipin to the CMG? This section of the manuscript would greatly benefit from some mutational analysis and biochemistry to support the significance of the interactions that are described in detail.

Collectively, the individual motifs of the TIMELESS MCM-plugin comprise almost the entire interface between TIMELESS and MCM and are highly conserved between yeast and human. They are

therefore almost certainly all important for correct TIMELESS-TIPIN replisome attachment. However, because there are multiple contact points, and TIMELESS and TIPIN also bind to DNA, we think mutation of only individual MCM-plugin elements may not completely disrupt TIMELESS-TIPIN replisome attachment. We think it would be much more informative to test the role of these motifs, and the DNA contacts made by TIMELESS and TIPIN, in functional replication assays. However, related to our responses to Referee 1, points 2 and 11, although we are developing a functional human DNA replication system that uses purified proteins in which we could test mutants, we are not quite there yet.

Fig. 6D:
The authors assign polarity to the helix in claspin-dependent density 2. In the figure, the density does not appear to be resolved well enough to assign N and C-termini. The authors should explain how they came to their conclusion.

We have now been able to assign the amino acid sequence of this helix that belongs to CLASPIN (Please see updated section on CLASPIN binding in the replisome and revised Figures 6 and EV5). Please see response to Referee #1, point 6 for details.

Fig. 7:
The authors point out that an invariant phenylalanine in Mcm7 serves as a separation pin during DNA strand separation. What effect do mutations of this residue have on CMG helicase activity and DNA synthesis? This should be tested experimentally.

Please see response to Referee #1, point 11.

Page 11, line 15: Fig. 6C should probably be EV6C.

Thank you for spotting this.

PDB validation reports are missing and should be included with the manuscript submission.

Cryo-EM maps used from model building have been deposited at EMDB and the coordinates for the atomic model have been submitted to the PDB. A full PDB validation report has been included with our revised submission.

Referee #3:

The authors provide a structural insight into the human replisome. This expands on the work by Rzechorzek et al, as it incorporates additional factors (Timeless-Tipin, Claspin) and achieves a higher resolution. The overall structure is consistent with work in yeast by the same group, but provide new structural insights about PolE and Claspin attachment. The structural description is in part very detailed and could be slightly shortened (see below), but seems to be fairly presenting results in the context of previously published work.

Major comments:
The description of the PolE Non-catalytic interface with the CMG is fairly descriptive. Either shorten it, or add mutants to verify some of the results.

We have streamline this section of the manuscript as requested.

Minor comments:
It would be informative to illustrate how much of each protein was modelled in form of a protein chain overview, and to show this in comparison to other structures from yeast and human.

We think this would be difficult to do neatly given the number of different replisome structures from other organisms. For the replisome proteins that bind CMG, none of which have been observed before in the human replisome at high resolution, we have included primary structure diagrams illustrating how much of the protein has been modelled.

The authors state on page 7, lines 9-12 "Although this suggests the majority of Claspin is not bound stably, several regions of density that could not be unambiguously modelled are visible (Fig 1C). These regions may therefore represent Claspin docking sites and we address this in a subsequent section". This is not clear - where are these regions?

As stated above (response to Referee #1, point 6), we have now been able to assign sequence to 3 regions of CLASPIN and have updated Figure 1C accordingly to clearly annotate these regions.

It is mentioned on page 8 line 14/15 that "there is poor sequence conservation between human and S.cerevisiae (Fig EV4D)". Is this also true for the specific amino acids making contact?

This is a good point. As requested by Referee #2 we have highlighted residues involved in protein:protein interactions in the alignments. Generally, the specific amino acids comprising the AND-1 interface are not well conserved between yeast and human but do occupy equivalent spatial positioning. We have made this clear in the revised manuscript (Page 8, lines 19-24).

The authors mention "POLE1nonCat adopts a polymerase fold with a wide-open jaw and C-terminal zinc finger (Fig 4B)". However, this is not clear from the figure (both open jaw and zinc finger location).

We have added a new figure panel (Fig EV4B) to illustrate this point.

The authors state "Whilst the resolution of docking site 1 is insufficient to unambiguously assign side-chain rotamers, the pattern of charged residues on the face of the POLE1 α22 helix interacting with Mcm2 is well conserved (Fig 6C)." I am not sure if Figure 6C shows this - maybe EV6C?

Thank you, the figure reference should indeed have been EV6C (EV4D in the revised manuscript).

The authors state "This interface is locked in place through a ring-stacking interaction between the well conserved F2138 of POLE1 and invariant Y821 of Mcm2". It is unclear which figure shows this - maybe EV6C - but here F2138 is labelled as P2138 - so this is confusing.

The Referee is correct and EV6C was mislabelled. Thank you for spotting this.

The authors suggest that Pol E is involved in the rate of replisome progression. However, the absence of DNA and flexibility in Mcm2 would rather argue for a release of the complex from DNA. Therefore, this statement should be corrected accordingly (this is better argued in the discussion).

At this point in the manuscript, we should have cited prior work that indicated Pol ε has a role in fork pausing. We have amended this section in the revised manuscript accordingly (Page 12, line 25 – Page 13, line 1).

Figure 6: The authors speculate that region 5 belongs to Mcm2. I think it would be better to remove this statement or alternatively make an experiment to proof the point. The whole section is too speculative and should be deleted.

We agree that the discussion about Mcm2 was rather speculative and have altered this section of the manuscript accordingly to better reflect the data (Page 14, lines 9-18). We have retained a description of the region 5 density as it does suggest that a replisome protein other that Mrc1/CLASPIN binds to this region of TIMELESS, but have removed the speculation about MCM2.

The authors describe the location of Claspin, but little functional relevance is mentioned. How could this be enhanced? (the discussion provides more info in this respect).

As mentioned above (please see response to Referee #1, point 6) we have now assigned amino acid sequence to 3 regions of CLASPIN. Consequently, we have remade Figure 6 and significantly altered the corresponding text. In doing so, we now provided greater discussion of the potential function(s) of the CLASPIN binding sites in the results and discussion.

The DNA binding analysis is interesting. Would be possible to highlight the most conserved amino acids in Figure 7CDEF e.g. with an asterisk or changing the colour of the font. Also, Figure 7H is nice, but could benefit from a zoomed out view - to understand the lagging strand channel in the context of the entire complex.

We thank the reviewer for these suggestions and have accommodated them in the revised manuscript.

In the discussion the authors suggest a role for Claspin in limiting CMG backtracking and slowing PolE during replisome progression and connecting this to cancer treatment. It would be sensible to reference this to a figure, to make the link stronger. It would be exciting to test this model by mutagenesis, but this appears not essential for publication.

We have reworked the discussion section on CLASPIN. We did not intend to connect these putative functions of CLASPIN to cancer treatment.

In the discussion the authors suggest that Pol ε can modulate C-tier configuration and therefore potentially CMG helicase activity. I think it is not clear if this is due to PolE activity, due to failed complex assembly or due to the effect of a different protein, so it would be good to revise the statement accordingly, or provide data that support the statement.

In addition to this work, we have obtained several structures in the complete absence of Pol ε and we always see significant disorder in the C-tier. Therefore, we don't think this just represents a small proportion of particles that have failed to assemble correctly. However, as we cannot include this data in the manuscript as it belongs to other projects, we have modified the results section to state that Pol ε might modulate C-tier configuration, whilst also recognising that the C-tier disorder could represent problems with complex assembly (Page 12, lines 20-25). We have also altered the wording in the discussion to say that Pol ε "might" alter C-tier configuration (Page 21, lines 7-9).

1st Revision - Editorial Decision 24th Sep 2021

Thank you for submitting your revised manuscript on human replisome structure for our consideration, and apologies of the delayed re-evaluation. Referees 1 and 2 have now returned their comments, copied below. As you will see, they both still retain concerns regarding functional follow-up experiments; but in light of your well-taken previous arguments for why such assays are presently not feasible and/or unlikely to offer key additional insight, and given that almost all other points appear to have been well-addressed, we shall nevertheless be happy to accept the study for EMBO Journal publication. The only issue I would still ask you to comment on/respond to in a final rebuttal letter (and potentially through additional explanations in the manuscript text) is referee 2's concern about clasping fitting, detailed in their report below.

Referee #1:

In this revised manuscript, Jones et al addressed the reference issues and made some changes to the text. However, the authors did not provide the necessary functional assays to validate their structural data. Without functional assays, this paper is simply a collection of structural data of the human core replisome, basically recapitulating most of the findings reported in two previously reported structures1,2. While the structures in this manuscript provide valuable information, it doesn't offer new information on the function of the eukaryotic replisome in catalysing DNA replication. It would be more appropriate to document their report in a more specialized journal.

1 Goswami, P., Abid Ali, F., Douglas, M. E., Locke, J., Purkiss, A., Janska, A., Eickhoff, P., Early, A., Nans, A., Cheung, A. M. C., Diffley, J. F. X. & Costa, A. Structure of DNA-CMG-Pol epsilon elucidates the roles of the non-catalytic polymerase modules in the eukaryotic replisome. Nat Commun 9, 5061, doi:10.1038/s41467-018-07417-1 (2018).
2 Baretić, D., Jenkyn-Bedford, M., Aria, V., Cannone, G., Skehel, M. & Yeeles, J. T. P. Cryo-EM Structure of the Fork Protection Complex Bound to CMG at a Replication Fork. Mol Cell 78, 926-940 e913, doi:10.1016/j.molcel.2020.04.012 (2020).

Referee #2:

In the revised version, the authors have addressed some of the concerns raised during the first review by making editorial changes to the manuscript and modifications to some figures. Furthermore the authors have updated the structural analysis of the interactions of claspin. Unfortunately, the authors have not included experiments related to the biochemical validation of the structure, interactions and the proposed mechanisms as suggested, which remains a major weakness of the manuscript.

In terms of the new atomic model for claspin, the fitting of the short segments into the cryoEM map seems rather speculative. There are many alpha helical regions in claspin apart from those fitted by the authors. Based on fig. 6, side chain densities are poorly resolved for sites 1-3 in the cryoEM map, with density being absent for bulky side chains or modelled side chains not fitting the density (for example R298, Y306 in 6F, W, F, K in 6G). Experimental validation of the model and claimed residue contacts would needed to ensure the data is not overinterpreted and the sequence has been assigned correctly.

Referee #1:

In this revised manuscript, Jones et al addressed the reference issues and made some changes to the text. However, the authors did not provide the necessary functional assays to validate their structural data. Without functional assays, this paper is simply a collection of structural data of the human core replisome, basically recapitulating most of the findings reported in two previously reported structures1,2. While the structures in this manuscript provide valuable information, it doesn't offer new information on the function of the eukaryotic replisome in catalysing DNA replication. It would be more appropriate to document their report in a more specialized journal.

1 Goswami, P., Abid Ali, F., Douglas, M. E., Locke, J., Purkiss, A., Janska, A., Eickhoff, P., Early, A., Nans, A., Cheung, A. M. C., Diffley, J. F. X. & Costa, A. Structure of DNA-CMG-Pol epsilon elucidates the roles of the non-catalytic polymerase modules in the eukaryotic replisome. Nat Commun 9, 5061, doi:10.1038/s41467-018-07417-1 (2018).
2 Baretić, D., Jenkyn-Bedford, M., Aria, V., Cannone, G., Skehel, M. & Yeeles, J. T. P. Cryo-EM Structure of the Fork Protection Complex Bound to CMG at a Replication Fork. Mol Cell 78, 926-940 e913, doi:10.1016/j.molcel.2020.04.012 (2020).

We respectfully disagree with some aspects of the Referee's assessment of our manuscript. In our revised manuscript we didn't simply make "*some changes to the text*". Rather, we were able to build a model for 3 regions of CLASPIN bound to the human replisome. This enabled us to re-evaluate existing data related to functional domains of CLASPIN. We believe that this represents an important addition to the manuscript that has not been appropriately acknowledged.

Referee #2:

In the revised version, the authors have addressed some of the concerns raised during the first review by making editorial changes to the manuscript and modifications to some figures. Furthermore the authors have updated the structural analysis of the interactions of claspin. Unfortunately, the authors have not included experiments related to the biochemical validation of the structure, interactions and the proposed mechanisms as suggested, which remains a major weakness of the manuscript.

In terms of the new atomic model for claspin, the fitting of the short segments into the cryoEM map seems rather speculative. There are many alpha helical regions in claspin apart from those fitted by the authors. Based on fig. 6, side chain densities are poorly resolved for sites 1-3 in the cryoEM map, with density being absent for bulky side chains or modelled side chains not fitting the density (for example R298, Y306 in 6F, W, F, K in 6G). Experimental validation of the model and claimed residue contacts would needed to ensure the data is not overinterpreted and the sequence has been assigned correctly.

We are confident that the atomic model we have built for 3 regions of CLASPIN is not overly speculative. The Referee is right to point out that there are multiple regions of CLASPIN that are predicted to be helical. The AlphaFold structure prediction for human CLASPIN (Jumper et al 2021) models 16 α-helical regions with high confidence (Figure R1A). Consequently, we systematically inspected the fit-to-density of all of these helical regions. Importantly, only the helices that we model as CLASPIN at sites 1-3 could be docked into the CLASPIN-dependent densities. Furthermore, for all three regions of CLASPIN, the model satisfies the chemical requirements for the interfaces between CLASPIN and the replisome. Additionally, the regions of CLASPIN that we have modelled are in very good agreement with the positioning of equivalent regions of Mrc1 in the yeast replisome determined by cross-linking mass spec (Baretic et al., Mol Cell 2020). In the revised manuscript, we have updated the main text to emphasise that we systematically inspected all

regions of CLASPIN that are predicted to be helical, and that our assignments are consistent with XL-MS data from the yeast replisome (Page 14, lines 23-25; Page 15, lines 5-8). In the Methods section we now provide a detailed description of how the CLASPIN model was constructed, including a thorough explanation of why we are confident that our assignment of CLASPIN sequence is correct. Please refer to Materials and Methods, Page 40, line 19 - Page 42, line 14 for details.

The referee suggests that side chain densities at sites 1-3 are poorly resolved based on Fig. 6. It is difficult to illustrate the quality of cryo-EM density in complex figures that show many different sidechains. We tried to only show density for CLASPIN and not the neighbouring subunits in an attempt to prevent the figures becoming too busy. While the density for CLASPIN at sites 1-3 is typically between 3.5-5 Å, inspection of the cryo-EM map, EMD-13375, shows that many side chains are sufficiently well resolved to permit the docking of segments of the AlphaFold structure prediction with high confidence and subsequent model refinement (please refer to Materials and Methods, Page 40, line 19 - Page 42, line 14). Moreover, while this manuscript has been in revision, we have obtained an additional cryo-EM map of the human replisome where the CLASPIN density at sites 1-3 is better resolved in certain regions. This new map will be published as part of a follow-up manuscript and provides further support for our model of CLASPIN. It has been deposited at the EMDB and will soon be publicly available with accession code EMD-13494.

Regarding the side chains specifically queried by the referee, there is well resolved density for CLASPIN R298, although we accept that this was not as clearly illustrated in Figure 6F as it might have been because we did not show density for TIMELESS Y474, with which it interacts. To better illustrate this region of the structure, we have updated Figure 6F to also include density for TIMELESS Y474. Cryo-EM density in this region from EMD-13494 is illustrated in Figure R1B. For CLASPIN Y306 there is clear density that accommodates a tyrosine residue and this is further illustrated in Figure R1C. Moreover, it is important to emphasise that, not only is there density for the Y306 side chain, there is well resolved density for the flanking residues on either side of Y306 that include a histidine (H307) and two proline residues (P305, P309) (Figure R1C). We accept that the fit to density for W538 in Figure 6G was not optimal and we have updated our model accordingly. We now provide a new panel for Figure 6G that shows the density for K532, F535 and W536. Side chain density for these residues, and for additional amino acids in the helix at site 2 (L531, H538 and A539), are also illustrated in Figure R1D.

A

Residue Numbers	Protein sequence	Modelled
170-192	KRRLEKEERKMEKIRQLKKKETK	
230-247	EESLESIRAAVKNKVKKH	
277-299	KAARLSKEALKQLHSETQRLIRE	Site #1
314-318	IHDFF	Site #1
330-334	AMALL	
492-499	FTLDRLKQ	
526-539	RELEALKQRFWKHA	Site #2
592-625	EKLQVLKAKLQEAMKLRRFEERQKRQALFKLDNE	Site #3
908-915	MDELLDLC	
935-944	ENMEELLNLC	
973-981	PMEEALALC	
1034-1045	EEELLKRSEKLK	
1091-1121	DEELQSQIKKIHMKTMLDDDKRQLRLYQERY	
1158-1189	DDQTEEQLDESEARWRKERIEREQWLRDMAQQ	
1205-1221	QFMILAKKVTAKALQKN	
1260-1270	KAVLQKLAALS	

B

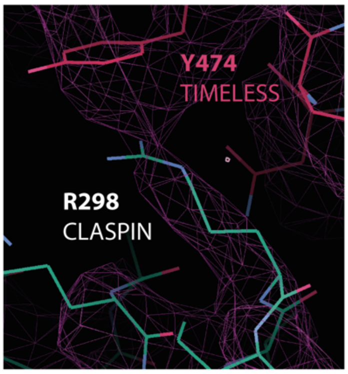

C

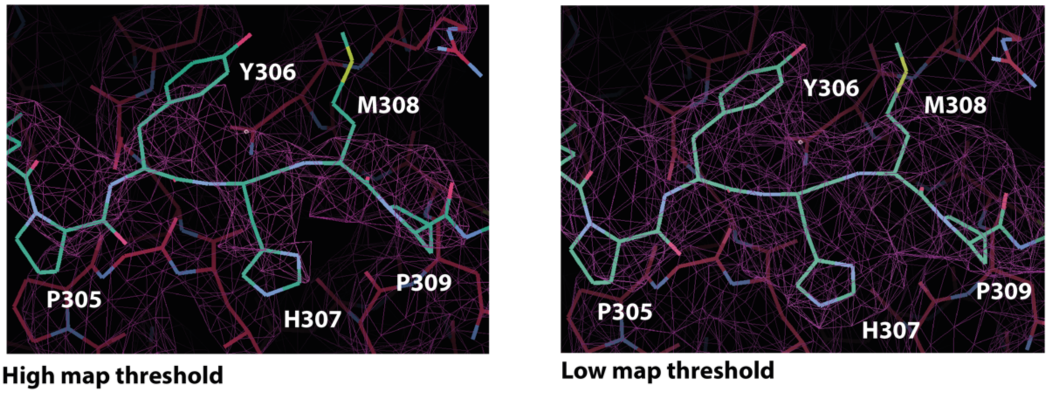

D

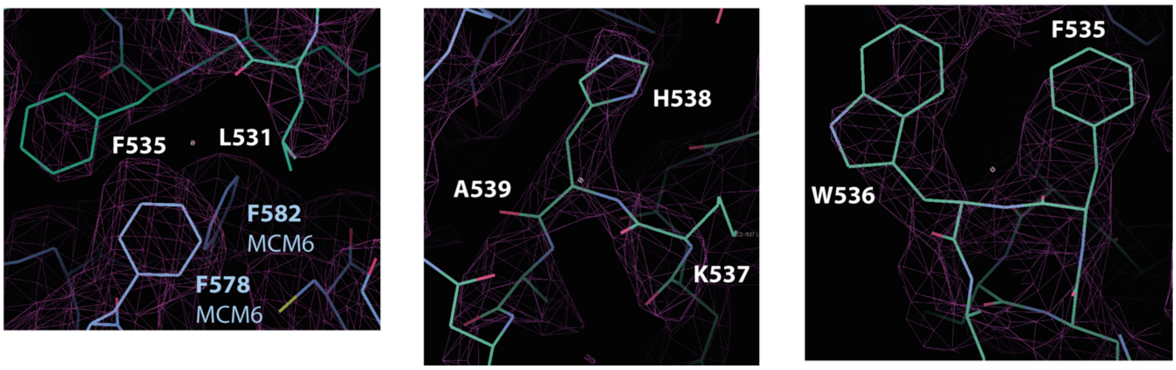

Figure R1. Supporting information for CLASPIN modelling
A Table listing the α-helices (greater than 3 residues in length) present in the AlphaFold model for *H. sapiens* CLASPIN. The single-letter amino acid sequence for each helix is coloured according to residue properties; hydrophilic (red) and aromatic (blue). **B-D** Model for the core human replisome (PDB: 7PFO) docked into cryo-EM density. The cryo-EM map used was obtained from an independent replisome preparation that also contained CLASPIN that will be published as part of a separate study with EMDB accession code EMD-13494. Cryo-EM density is coloured pink and the CLASPIN model is coloured green. **B** CLASPIN residue R298 contacts TIMELESS residue Y474. **C** CLASPIN residues 305-309 at site 1 fit the cryo-EM density well, with side-chain density present at both high (left) and low (right) thresholds. **D** Selected residues at CLASPIN site 2. Clear side-chain density is present for CLASPIN residues F353 and L531 interacting with a hydrophobic patch formed by TIMELESS residues F578 and F582 (left), H538 and A539 (middle) and W536 (right).

Thank you for submitting your final revised manuscript for our consideration. I am pleased to inform you that we have now accepted it for publication in The EMBO Journal.

EMBO PRESS

YOU MUST COMPLETE ALL CELLS WITH A PINK BACKGROUND ↓

PLEASE NOTE THAT THIS CHECKLIST WILL BE PUBLISHED ALONGSIDE YOUR PAPER

Corresponding Author Name: Joseph Yeeles
Journal Submitted to: EMBO
Manuscript Number: EMBOJ-2021-108819

Reporting Checklist For Life Sciences Articles (Rev. June 2017)

This checklist is used to ensure good reporting standards and to improve the reproducibility of published results. These guidelines are consistent with the Principles and Guidelines for Reporting Preclinical Research issued by the NIH in 2014. Please follow the journal's authorship guidelines in preparing your manuscript.

A- Figures
1. Data
The data shown in figures should satisfy the following conditions:

- ➔ the data were obtained and processed according to the field's best practice and are presented to reflect the results of the experiments in an accurate and unbiased manner.
- ➔ figure panels include only data points, measurements or observations that can be compared to each other in a scientifically meaningful way.
- ➔ graphs include clearly labeled error bars for independent experiments and sample sizes. Unless justified, error bars should not be shown for technical replicates.
- ➔ if n< 5, the individual data points from each experiment should be plotted and any statistical test employed should be justified
- ➔ Source Data should be included to report the data underlying graphs. Please follow the guidelines set out in the author ship guidelines on Data Presentation.

2. Captions

Each figure caption should contain the following information, for each panel where they are relevant:

- ➔ a specification of the experimental system investigated (eg cell line, species name).
- ➔ the assay(s) and method(s) used to carry out the reported observations and measurements
- ➔ an explicit mention of the biological and chemical entity(ies) that are being measured.
- ➔ an explicit mention of the biological and chemical entity(ies) that are altered/varied/perturbed in a controlled manner.

- ➔ the exact sample size (n) for each experimental group/condition, given as a number, not a range;
- ➔ a description of the sample collection allowing the reader to understand whether the samples represent technical or biological replicates (including how many animals, litters, cultures, etc.).
- ➔ a statement of how many times the experiment shown was independently replicated in the laboratory.
- ➔ definitions of statistical methods and measures:
  - common tests, such as t-test (please specify whether paired vs. unpaired), simple χ^2 tests, Wilcoxon and Mann-Whitney tests, can be unambiguously identified by name only, but more complex techniques should be described in the methods section;
  - are tests one-sided or two-sided?
  - are there adjustments for multiple comparisons?
  - exact statistical test results, e.g., P values = x but not P values < x;
  - definition of 'center values' as median or average;
  - definition of error bars as s.d. or s.e.m.

Any descriptions too long for the figure legend should be included in the methods section and/or with the source data.

In the pink boxes below, please ensure that the answers to the following questions are reported in the manuscript itself. Every question should be answered. If the question is not relevant to your research, please write NA (non applicable). We encourage you to include a specific subsection in the methods section for statistics, reagents, animal models and human subjects.

USEFUL LINKS FOR COMPLETING THIS FORM

http://www.antibodypedia.com
http://1degreebio.org
http://www.equator-network.org/reporting-guidelines/improving-bioscience-research-repor
http://grants.nih.gov/grants/olaw/olaw.htm
http://www.mrc.ac.uk/Ourresearch/Ethicsresearchguidance/Useofanimals/index.htm
http://ClinicalTrials.gov
http://www.consort-statement.org
http://www.consort-statement.org/checklists/view/32-consort/66-title
http://www.equator-network.org/reporting-guidelines/reporting-recommendations-for-tumo
http://datadryad.org
http://figshare.com
http://www.ncbi.nlm.nih.gov/gap
http://www.ebi.ac.uk/ega
http://biomodels.net/
http://biomodels.net/miriam/
http://jjj.biochem.sun.ac.za
https://osp.od.nih.gov/biosafety-biosecurity-and-emerging-biotechnology/
http://www.selectagents.gov/

B- Statistics and general methods

Please fill out these boxes ↓ (Do not worry if you cannot see all your text once you press return)

1.a. How was the sample size chosen to ensure adequate power to detect a pre-specified effect size?	N/A
1.b. For animal studies, include a statement about sample size estimate even if no statistical methods were used.	N/A
2. Describe inclusion/exclusion criteria if samples or animals were excluded from the analysis. Were the criteria pre-established?	N/A
3. Were any steps taken to minimize the effects of subjective bias when allocating animals/samples to treatment (e.g. randomization procedure)? If yes, please describe.	N/A
For animal studies, include a statement about randomization even if no randomization was used.	N/A
4.a. Were any steps taken to minimize the effects of subjective bias during group allocation or/and when assessing results (e.g. blinding of the investigator)? If yes please describe.	N/A
4.b. For animal studies, include a statement about blinding even if no blinding was done	N/A
5. For every figure, are statistical tests justified as appropriate?	N/A
Do the data meet the assumptions of the tests (e.g., normal distribution)? Describe any methods used to assess it.	N/A
Is there an estimate of variation within each group of data?	N/A

Is the variance similar between the groups that are being statistically compared?	N/A

C- Reagents

6. To show that antibodies were profiled for use in the system under study (assay and species), provide a citation, catalog number and/or clone number, supplementary information or reference to an antibody validation profile. e.g., Antibodypedia (see link list at top right), 1DegreeBio (see link list at top right).	N/A
7. Identify the source of cell lines and report if they were recently authenticated (e.g., by STR profiling) and tested for mycoplasma contamination.	N/A

* for all hyperlinks, please see the table at the top right of the document

D- Animal Models

8. Report species, strain, gender, age of animals and genetic modification status where applicable. Please detail housing and husbandry conditions and the source of animals.	N/A
9. For experiments involving live vertebrates, include a statement of compliance with ethical regulations and identify the committee(s) approving the experiments.	N/A
10. We recommend consulting the ARRIVE guidelines (see link list at top right) (PLoS Biol. 8(6), e1000412, 2010) to ensure that other relevant aspects of animal studies are adequately reported. See author guidelines, under 'Reporting Guidelines'. See also: NIH (see link list at top right) and MRC (see link list at top right) recommendations. Please confirm compliance.	N/A

E- Human Subjects

11. Identify the committee(s) approving the study protocol.	N/A
12. Include a statement confirming that informed consent was obtained from all subjects and that the experiments conformed to the principles set out in the WMA Declaration of Helsinki and the Department of Health and Human Services Belmont Report.	N/A
13. For publication of patient photos, include a statement confirming that consent to publish was obtained.	N/A
14. Report any restrictions on the availability (and/or on the use) of human data or samples.	N/A
15. Report the clinical trial registration number (at ClinicalTrials.gov or equivalent), where applicable.	N/A
16. For phase II and III randomized controlled trials, please refer to the CONSORT flow diagram (see link list at top right) and submit the CONSORT checklist (see link list at top right) with your submission. See author guidelines, under 'Reporting Guidelines'. Please confirm you have submitted this list.	N/A
17. For tumor marker prognostic studies, we recommend that you follow the REMARK reporting guidelines (see link list at top right). See author guidelines, under 'Reporting Guidelines'. Please confirm you have followed these guidelines.	N/A

F- Data Accessibility

18: Provide a "Data Availability" section at the end of the Materials & Methods, listing the accession codes for data generated in this study and deposited in a public database (e.g. RNA-Seq data: Gene Expression Omnibus GSE39462, Proteomics data: PRIDE PXD000208 etc.) Please refer to our author guidelines for 'Data Deposition'.<br><br>Data deposition in a public repository is mandatory for:<br>a. Protein, DNA and RNA sequences<br>b. Macromolecular structures<br>c. Crystallographic data for small molecules<br>d. Functional genomics data<br>e. Proteomics and molecular interactions	Cryo-EM density maps of the human replisome used in model building have been deposited in the Electron Microscopy Data Bank (EMDB) under the following accession numbers: EMD-13375 (full complex, consensus refinement), EMD-13377 (multi-body refinement, Pol e/CDC45/GINS), EMD-13376 (multi-body refinement, AND-1/CDC45/GINS), EMD-13384 (minus CLASPIN, consensus refinement). Atomic coordinates have been deposited in the Protein Data Bank (PDB) with the accession number PDB: 7PFO for the complete core human replisome.
19. Deposition is strongly recommended for any datasets that are central and integral to the study; please consider the journal's data policy. If no structured public repository exists for a given data type, we encourage the provision of datasets in the manuscript as a Supplementary Document (see author guidelines under 'Expanded View' or in unstructured repositories such as Dryad (see link list at top right) or Figshare (see link list at top right).	See section 18, cryo-EM maps have been deposited in the EMDB and atomic models in the PDB.
20. Access to human clinical and genomic datasets should be provided with as few restrictions as possible while respecting ethical obligations to the patients and relevant medical and legal issues. If practically possible and compatible with the individual consent agreement used in the study, such data should be deposited in one of the major public access-controlled repositories such as dbGAP (see link list at top right) or EGA (see link list at top right).	N/A
21. Computational models that are central and integral to a study should be shared without restrictions and provided in a machine-readable form. The relevant accession numbers or links should be provided. When possible, standardized format (SBML, CellML) should be used instead of scripts (e.g. MATLAB). Authors are strongly encouraged to follow the MIRIAM guidelines (see link list at top right) and deposit their model in a public database such as Biomodels (see link list at top right) or JWS Online (see link list at top right). If computer source code is provided with the paper, it should be deposited in a public repository or included in supplementary information.	N/A

G- Dual use research of concern

22. Could your study fall under dual use research restrictions? Please check biosecurity documents (see link list at top right) and list of select agents and toxins (APHIS/CDC) (see link list at top right). According to our biosecurity guidelines, provide a statement only if it could.	N/A
